# Gender and Accuracy in Decoding Affect Cues: A Meta-Analysis

**DOI:** 10.3390/jintelligence13030038

**Published:** 2025-03-18

**Authors:** Judith A. Hall, Sarah D. Gunnery, Katja Schlegel

**Affiliations:** 1 Department of Psychology, Northeastern University, Boston, MA 02115, USA; 2 Department of Psychology, New England College, Henniker, NH 03242, USA; sgunnery@nec.edu; 3 Institute of Psychology, University of Bern, 3012 Bern, Switzerland; katja.schlegel@unibe.ch; 4 Institute of Psychology, Czech Academy of Sciences, 602 00 Brno, Czech Republic

**Keywords:** gender, emotion recognition, decoding, cues, affect

## Abstract

Gender differences in understanding the meanings of affect cues, often labeled emotion recognition, have been studied for over a century. Past reviews of the literature have concluded that girls and women score higher than boys and men on tests of accuracy in decoding affect cues, which are most often tested in the cue modalities of face, body, and content-free voice. The present meta-analysis updates knowledge on this topic by including many more studies (1188 effect sizes in 1011 studies; total *N* = 837,637) and examining a wide range of moderators such as health status of sample, international location, cue channels of the test, and other sample and test characteristics. Indeed, the gender difference favoring girls and women still exists, and evidence for publication bias was weak. The difference is not large (*r* = 0.12, *d* = 0.24), but it is extremely consistent across many moderators, which, even when significant, show minor differences. Health status was the only moderator to produce groups without a significant gender difference.

## 1. Introduction

Societal and scientific understandings of gender, and categorizations based on gender, are ever shifting. Yet, similarities and differences according to gender will always be studied and debated. Continued study of gender differences is performed not only to achieve a thorough picture, but also to weigh the importance of any differences that are revealed, ponder the origins of these differences, and contribute to understanding the construction of gender more broadly. A long-running research tradition on psychological gender differences concerns accuracy in interpreting affect cues, broadly defined. Early on, and to this day, this endeavor has mainly taken the form of asking people to assign “basic” emotion labels to facial expressions, although other cue modalities and many other expressive states are also studied. Researchers have been looking at gender differences in decoding affect cues for 100 years and from the start have theorized about origins and consequences, as did Gates (1923): “Perhaps the superior ‘social tact’ attributed to women is due to better ability to interpret the emotional expressions of others” (p. 455).

As we review below, the consensus in previous meta-analyses is that girls and women are more accurate at judging the meanings of expressive cues than boys and men are (e.g., Thompson and Voyer 2014), a difference that laypeople are aware of, as captured in their beliefs about the difference (Briton and Hall 1995). This gender difference stands in contrast to overall intelligence, where gender differences are negligible (Halpern and LaMay 2000; Giofrè et al. 2022; Longman et al. 2007; Roivainen 2011), but both historically and stereotypically, it has been thought that men have higher mathematical (Cvencek et al. 2011) and spatial intelligence (Halpern et al. 2011), while women have higher verbal and emotional intelligence (Petrides et al. 2004). The actual differences are typically small, vary by cognitive domain, and can be highly task-dependent (Giofrè et al. 2022; Hyde et al. 1990; Hyde and Linn 1988). For instance, studies using the Wechsler Intelligence Scales for Children (WISC) and the Wechsler Adult Intelligence Scale (WAIS-III) report negligible gender differences in fluid reasoning and working memory (Giofrè et al. 2022; Longman et al. 2007; Roivainen 2011), but a small female advantage in processing speed (*d* = 0.31−0.37 (Giofrè et al. 2022; Longman et al. 2007)). In visual–spatial processing and perceptual organization, these studies found a small male advantage (*d* = 0.12−0.21 (Giofrè et al. 2022; Longman et al. 2007)), while in another meta-analysis, Maeda and Yoon (2013) found a larger male advantage in 3D mental rotation, as measured by the Purdue Spatial Visualization Tests (Hedges’ *g* = 0.57). Regarding verbal abilities, evidence suggests a small male advantage in verbal and reading comprehension (*d* = 0.14−0.29 (Giofrè et al. 2022; Longman et al. 2007; Petersen 2018)), whereas women tend to outperform men in other verbal performance tasks such as writing (*d* = 0.45 (Petersen 2018)). The finding that gender differences in other areas of intelligence can be highly task-dependent calls for a nuanced and detailed investigation of the longstanding gender difference in decoding affective states.

The ability to accurately judge affective cues is well established as a valuable ability and is correlated with adaptive qualities (Schmid Mast and Hall 2018). Although accurate affect perception can be misused or overused (Schlegel 2020), the vast weight of evidence suggests that being accurate at judging affect cues is an asset both for individuals and for interpersonal interactions. Examples include negotiation success (Elfenbein et al. 2007), favorable peer ratings (Funder and Harris 1986), success as a music teacher (Kurkul 2007), success as a salesperson (Byron et al. 2007), better performance ratings by supervisors and satisfaction ratings by subordinates in one’s company (Byron 2007), less aggression in children (Acland et al. 2023), and healthy personality traits and social competencies (Hall et al. 2009; Schlegel et al. 2019).

These findings align with the conceptualization of affect decoding accuracy as a fundamental component of emotional intelligence (EI) across various ability-based models (e.g., Elfenbein and MacCann 2017; Fiori et al. 2022; Joseph and Newman 2010; Mayer et al. 2016). Specifically, affect decoding accuracy is often considered a prerequisite for understanding the causes and consequences of emotional situations and for effectively managing them (Mayer et al. 2016). For example, the cascading model of EI posits that accurate affect perception predicts job performance through improved emotional understanding and management (Joseph and Newman 2010). Like affect decoding accuracy, the broader construct of ability EI is positively linked with workplace performance (Schlegel et al. 2024), daily emotional experiences (MacCann et al. 2020), and romantic relationship satisfaction (Jardine et al. 2022).

Studies suggest that affect decoding skill can be improved through training (e.g., Schlegel et al. 2017), which may affect interpersonal behaviors. For instance, in one study, participants who underwent an emotion recognition training task (versus a control task) were more egalitarian, more positive in affect, and less dominant in a later negotiation exercise (Schlegel 2021). The question of who has an edge in this skill could matter, therefore, in many domains of social life. Women and girls’ edge, even if not large in magnitude, could matter for them and the people around them.

At the outset, we acknowledge the lack of nuance in our treatment of the term “gender”. Virtually all the available studies operationally defined gender (or, as an alternate term, sex) using a binary distinction typically obtained from participants’ self-reports of binary gender identity. The number of individuals with gender identities outside of this prescribed binary cannot be known, and as a result, we resort to the binary male–female distinction mandated by the field’s historical treatment of gender. While base rates point to the great majority of participants being cisgender boys or men and girls or women (Herman et al. 2022), still a small amount of uncertainty remains that could, hypothetically, contribute to variance between studies. The current meta-analysis includes both children and adults so we will use the term females to refer to people who self-reported being a woman, girl, or female and males to refer to people who self-reported being a man, boy, or male, while recognizing the limitations of the nomenclature (American Psychological Association 2022).

We describe the present meta-analysis as being about “affect” rather than “emotion” because we wish to remain agnostic on the fraught question of what, exactly, an “emotion” is and therefore we avoided drawing strict or arbitrary lines. Therefore, we consider many varieties of affective states (spontaneously expressed or posed for the researcher), not only the short list of discrete emotions that are most often referred to as “basic” or universal (Ekman et al. 1987). Although many studies in our database drew on a prototypical list (anger, fear, surprise, sadness, happiness, disgust), we expanded the domain of potentially informative expressive states to include, for example, target persons’ ratings of their feelings on a negativity–positivity scale (Zaki et al. 2008), the situationally defined affective scenes in the Profile of Nonverbal Sensitivity test (e.g., is the target person talking to a lost child or criticizing someone for being late (Rosenthal et al. 1979)), the expressive states asked about in the Reading the Mind in the Eyes Test (e.g., is the person serious, ashamed, alarmed, or bewildered), combined scoring of targets’ self-described thoughts and feelings (Hodges and Kezer 2021), and other, sometimes extensive, lists of internal states (e.g., confusion, coyness, awe, adoration, anxiety (Monroy et al. 2022)).

The present meta-analysis covers only studies where multiple perceivers made judgments about recorded stimuli containing eyes, face, body/hands, postural, and/or voice cues. Studies of unique target–perceiver dyads are not included, for example, research employing the live dyadic version of the “empathic accuracy” paradigm (Ickes et al. 1990) in which participants infer the dyadic partner’s thoughts and feelings right after their interaction. Dyadic studies are not considered here because of the often-noted sender–receiver confound that presents a challenge to isolating perceiver-based accuracy (a problem first discussed by (Alkire et al. 1968)). Thus, the present database includes only studies that showed one or more recorded target persons to a group of perceivers.

### Past Meta-Analyses

We are aware of seven, only minimally overlapping, meta-analyses of males’ and females’ accuracy in decoding recorded cues, six of which covered exclusively the judgment of affect cues (Hall 1978; Hall et al. 2016; Kirkland et al. 2013; McClure 2000; Rosenthal et al. 1979; Thompson and Voyer 2014) while one included judgments of more heterogeneous qualities such as personality traits (Hall 1984). The overview shown in Table 1 conveys a consensus on female advantage, expressed as the point biserial (Pearson) correlation, which can, for effects in this range, be doubled to yield the Cohen’s *d* index that describes a difference in terms of standard deviation units. The table is only a rough guide, however, as the reviews differed in search methods and terms, years covered, population characteristics (e.g., age, nationality), cue channels, stimulus content, and specific testing instruments (see Appendix A for description of commonly used tests).

The current meta-analysis differs from, and builds on, previous meta-analyses in a number of ways. The largest (Thompson and Voyer 2014) included only basic emotions, included results for single emotions (not just totals), combined accuracy with response latency, and was limited to nonclinical samples. It also relied almost exclusively on published results. The current meta-analysis not only updates the previous reviews by about 10 years, it also more than doubles the number of effect sizes and includes a large number of unpublished findings. In addition, it assesses an important moderator not previously included in a meta-analysis: perceivers’ health status.

Table 1 lists the moderators included in each of the prior meta-analyses; we describe the results of these past analyses in the Results section where relevant. The moderators assessed in the current work include document year, age of perceivers, age and gender of stimulus persons (targets), ethnicity of perceivers and targets, country/region of data gathering, physical and mental health status of perceivers, gender of first author, name of specific test, number of test items, cue modalities, whether the test was static versus dynamic, and whether the cues were posed or not. All of these variables are described in the Method section and, in more detail, in Supplementary Materials File S3. The data for the present meta-analysis can be found in Supplementary Materials Files S4 and S5.

## 2. Method

### 2.1. Definition of Key Concepts

#### 2.1.1. Gender

Gender was operationally defined as in the literature under review, namely in terms of study participants’ self-description using terms such as male or female or, for children, it could be a label provided by an adult.

#### 2.1.2. Accuracy

Accuracy was defined as correct judgment of a target person’s affective states (excluding physical pain except when pain was embedded with many other states) conveyed via face, hands, body, voice (with or without potentially diagnostic verbal content), or combinations of these. The great majority of tasks included some number of “basic” emotions (fear, surprise, happiness, disgust, anger, sadness).

Accuracy was most often measured in terms of the number or percentage of correct answers on the test items. Other scoring metrics included a correlation (over time within an interaction or over targets) between the perceiver’s rating of affect and the target’s self-rated affect, and coders’ scoring of the match between the perceiver’s guess of the target’s thoughts/feelings and the target’s self-report of the same.

#### 2.1.3. Test

The term “test” refers to a task consisting of recorded stimuli that perceivers judged, and which could be scored for accuracy based on a researcher-defined criterion. The criterion in this literature was most often a state that the target person deliberately expressed, but criteria could also be targets’ self-reported state (for example, after recounting an emotional experience), consensus of observers, or the nature of stimuli to which the target persons were exposed, which were intended to produce spontaneous affective expressions (for example, pictures eliciting pleasant feelings). A test did not have to be a formally developed instrument with demonstrated psychometric qualities or construct validation; although many named tests were used, there were also many studies where the test was designed uniquely for the study.

We used only “total” scores for a given test. A study in which two different tests were given would produce a gender effect for each test; a study where the stimuli originated from one test but were separately scored and reported (e.g., face items separately scored from voice items) was considered to produce two total scores.

### 2.2. Search

The term “source” refers to a given document, and the term “study” refers to an independent group of perceivers within a source. A given source may contain more than one study.

All systematic searches were conducted on PsycInfo. Because interpersonal accuracy does not have standard nomenclature, an assortment of search terms was required (emotion recognition; nonverbal sensitivity; empathic accuracy; decoding nonverbal cues; nonverbal decoding; decoding of nonverbal; decoding affective). Very large searches (the biggest by far being “emotion recognition”) used the additional filter “quantitative study” when that was available (starting in 2003), and “empirical study” before that. Over 20 individual tests were searched by name (see Supplementary Materials File S1 for list).

Over 700 emails were sent to authors of eligible sources published from 2015 and later that came up in the systematic searches, requesting gender-difference results that had not been included in the publication. Additional sources were accumulated by serendipity and by searching the bibliographies of published meta-analyses. Unpublished data were solicited on the member distribution lists (listserves) of the Society for Personality and Social Psychology, International Society for Research on Emotion, and the German Psychological Society.

Because of the great variety in terminology used by authors of studies on affective accuracy, we cannot claim to have located all potentially eligible studies. However, great care was taken to avoid bias in selecting studies for examination. To this end, we never tracked down sources cited in authors’ introduction or discussion sections because these could be biased by those authors’ own biases and the nature of their findings (such sources would of course be considered if located via one of the other, unbiased methods listed above). Similarly, we did not solicit results directly from specific researchers (except to solicit results from works retrieved in our systematic searches, as described above), because of the chance of bias in whom we would ask, who would respond, and what they would choose to send or not send. Finally, all exclusion decisions were made without knowledge of study outcomes.

Formal searches had no beginning date of publication and went to the end of 2022 except for the “emotion recognition” search, which ended in mid-2022. Dates extending into 2023 were allowed for sources found via serendipity and by call for unpublished results. Potentially eligible sources could be a published article, chapter, or book, or an unpublished thesis, dissertation, or other unpublished dataset, and had to be in English or translatable by us. We included studies from previous meta-analyses on gender differences in accuracy if they met present inclusion criteria, but we calculated our own effect sizes unless we could not do so, in which case we used the effect sizes reported in the earlier meta-analysis.

### 2.3. Inclusion Criteria for Perceivers

Perceiver groups met these requirements: (1) at least 15 male and 15 female perceivers; (2) minimum age of 8 years for all perceivers in a study, a cutoff that ensures the children’s task capabilities (Wang et al. 2024); (3) a fairly continuous range of perceiver ages (if there were distinct and discontinuous perceiver age groups, these were considered separate studies if possible, and if not, the study was excluded); (4) perceivers were nonclinical or clinically diagnosed, the latter meaning the group was clinically diagnosed with a psychopathology, medical condition, or other developmental or incurred disability; (5) all nationalities, cultures, and ethnicities of perceivers and targets were included; (6) in experimental studies, results across conditions were included if the study’s experimental manipulation was not designed to change the gender difference; (7) perceivers did not represent extremes on another variable (e.g., a sample containing only people very high and very low on extraversion would be excluded).

### 2.4. Inclusion Criteria for Test Characteristics

Included tests had the following features: (1) the stimuli were human beings or human-looking digital avatars; (2) stimuli were face (whole face or eye region), body/hands, posture, and/or vocal cues (vocal cues could be unaltered speech/speech sounds, or speech whose verbal intelligibility was masked via bandpass filtering, random scrambling of speech segments, foreign or nonsense language, or sentences with emotionally neutral or ambiguous content spoken to convey different affects); (3) stimuli were affect as described in the Introduction (also, not considered were judgments of characteristics that often have affective correlates but which are not directly about affect such as truth versus lie, status/dominance, personality, intelligence, age, nationality, religion, or relationship status among targets); (4) the test had at least two stimuli. Response formats included multiple choice, ratings made numerically or with a manual slider, and free response.

### 2.5. Exclusions Not Mentioned Above

Other grounds for exclusion were the following: (1) reaction time (response latency) instead of accuracy as we defined it; (2) matching tasks (for example, judgments of whether two faces showed the same emotion); (3) stimuli that were shown upside down or sideways; (4) test context designed to impede or promote accuracy (e.g., experimenter created distracting, biasing, priming, or confusing cues in the environment before or while measuring accuracy); (5) no clear correct answer (e.g., the top and bottom of the face were mismatched on affective state); (6) point-light studies where the stimuli are illuminated dots on key body joints in an otherwise black field, isolating perception of body movement; (7) clinical and nonclinical groups were combined.

### 2.6. Reliability of Search Procedure

Search decision reliability was based on the completed years 2022, 2021, 2020, 2010, and 2009 for the search term “emotion recognition”. We used a random 10% of each year’s retrieved sources, resulting in 73 studies. The two coders (J.A.H. and S.D.G.) agreed on whether the study was eligible in 70 of the studies, for a rate of 96% agreement. This reliability was deemed acceptable with the discrepancies (e.g., was the task measuring accuracy or norming a database without an accuracy criterion) being due to judgment rather than coding errors. After discussion between coders, these three studies were excluded.

### 2.7. Flow Chart of Screening

**Figure 1:**
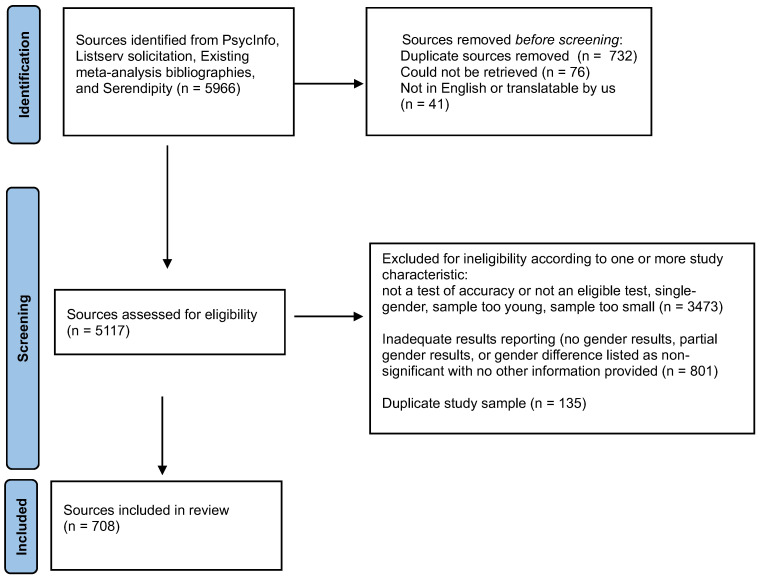
PRISMA study search and eligibility screening process (Page et al., 2021).

The total number of screened sources was 5966. Of these, 708 produced one or more effect sizes (in the source or on request) and these comprised the database analyzed in the present article. (See Supplementary Materials File S2 for citations to the included sources.) The Preferred Reporting Items for Systematic Reviews and Meta-Analysis (PRISMA) flow chart (Figure 1) illustrates why 5258 sources were excluded. Of note, 141 sources were not used because they provided only the statement that the gender effects were “nonsignificant” or presented only partial gender results for the accuracy test (e.g., for only some of the emotions included in the test). Although sometimes meta-analysts impute an effect size of zero for “nonsignificant” results, we did not do that because of the strong a priori likelihood that unknown effect sizes favored females. Therefore, studies for which “nonsignificant” was the only information available were omitted from analysis. Studies reporting only partial results were omitted because these would likely have been the only significant comparisons and therefore overestimates of the test’s overall gender result.

### 2.8. Database Characteristics

#### 2.8.1. Study Characteristics

Table 2 describes the 1011 independent studies. To summarize, most studies came from PsycInfo searches, with the search term “emotion recognition” yielding the most usable studies followed by one of the specific tests (Reading the Mind in the Eyes Test, or RMET (Baron-Cohen et al. 2001)). Of the studies located in PsycInfo, approximately equal proportions of their results were in the published source versus sent on request. Document dates spanned from the 1930s but both the mean and median year fell within the last decade of located sources. The grand total of perceivers exceeded 800,000. A few extremely large samples pulled up the mean sample size; a more realistic view comes from the median sample size of 110.

#### 2.8.2. Perceiver Characteristics

Summarizing Table 2, studies were roughly split between younger and older adults, with a generous proportion of community members. Slightly more than one-third of studies were in the USA and slightly fewer than one-third were in non-Anglophone Europe, with the other groups spread across the world. Four-fifths were nonclinical samples, with the largest subgroup of clinical samples having psychiatric diagnoses. Two-thirds of the groups were White or majority White in the studies where this was reported.

#### 2.8.3. Test Characteristics

Description of testing instruments (Table 3) is based on results associated with the 1188 effect sizes. When assigning codes to different tests, all versions of a named test were given the same test code as long as they used items from the original stimulus set, meaning that a given test code can include administrations that varied in number and nature of items, alteration in stimuli (e.g., morphed from the original still photograph to produce either static stimuli of different intensities or the illusion of motion), and exposure duration. Because the number of different testing instruments was great, Table 3 names only tests that were used in 20 or more effect sizes. Clearly, the RMET led with nearly a third of all results; the Pictures of Facial Affect (POFA (Ekman and Friesen 1976)) came in a far second. Targets in the tests were mainly both male and female, adult, and White (when known). Cue channel was mainly whole face, with eyes only coming in second; most stimuli were static (faces that were morphed from photographs, sometimes made to give the illusion of movement, were coded as static), and most were posed.

### 2.9. Reliability of Study Coding and Effect Size Coding

Reliability was assessed for study coding and effect size extraction. Two coders (J.A.H. and S.D.G.) double coded effect sizes for 5% (*n* = 37) of the samples included from the years 2022, 2021, 2020, 2010, and 2009. Reliability was 95%. Most disagreements were due to rounding differences, which were corrected between coders.

### 2.10. Effect Size Coding and Statistical Analysis

The effect size used in the present research was the point biserial (Pearson) correlation between gender and accuracy, coded such that a positive sign indicated higher performance by females and a negative sign indicated higher performance by males. When authors did not report this correlation directly, we calculated the effect size from means and standard deviations or from *t*-tests. A small number of effects were available only from analyses that were adjusted by their original authors for covariates either in multiple regression (and reported as standardized regression coefficients) or were adjusted for other variables in a multifactorial analysis of variance. These adjusted effects were retained in the analysis due to their small number and their negligible overall effect (see further discussion of adjusted effects in later sections). All analyses were based on the Fisher-*z* (*rz*) normalization (for correlations of 0.24 and smaller, the *r* and *rz* are the same).

Overall effect size was determined with a multilevel meta-analysis (MLM) accounting for the nesting of effect sizes within studies (samples) and incorporating the precision of the effect size estimates (i.e., the sampling variances of the Fisher z-transformed effect sizes) using the metafor package in R (Viechtbauer 2010). This model is equivalent to a weighted random-effects model (Hall and Miller 2025). Restricted Maximum Likelihood (REML) estimation was used to fit the model.

Moderator analyses were conducted by adding each categorical or continuous moderator separately to the basic model of the overall effect. For categorical moderators, the effect size per subgroup or category was obtained by excluding the intercept; differences in effect size between categories were obtained by setting a reference category as the intercept and comparing it to each of the other categories. For all moderators except “specific test”, categories with at least 10 effect sizes were analyzed. Due to the large number of specific tests or test categories (21) that were used at least 10 times, only tests/categories with at least 20 effect sizes were included in that moderator analysis. The omnibus test for the effect of each categorical moderator as a whole (indexed by the test statistic QM) was obtained from the model including the intercept, and results (effect size estimates per subgroup or category) are reported as correlations.

## 3. Results

### 3.1. Overall Gender Difference

**Figure 2:**
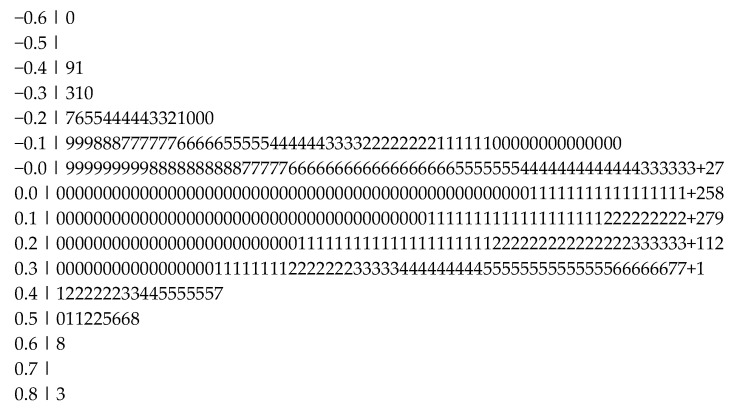
Stem-and-leaf plot for the 1188 effect sizes (*r*).

For all 1188 gender effect sizes, the unweighted mean *rz* was 0.12 (*SD* = 0.15), equivalent to a Cohen’s *d* of 0.24. The distribution was symmetrical, with a median also of 0.12. Skewness and kurtosis were well within the normal range (see also the stem-and-leaf plot in Figure 2). The range of effects (*r*) was −0.60 to 0.83; removing three positive and three negative values that were ±3 *SD* from the mean also yielded a mean of 0.12. Because of their symmetrical effect, these six effects were left in the database. Eighty-four percent of all non-zero effects favored females in direction (956/1137), a figure in line with previous, much smaller, meta-analytic reviews. The overall effect size obtained with the weighted random-effects MLM was *r* = 0.12, the same as in the unweighted analysis, with a 95% CI of [0.11; 0.13]. Between-study variance τ^2^ was 0.01, and significant heterogeneity was detected among the effect sizes (Q(*df* = 1187) = 325.74, *p* < .001).

### 3.2. Sample Characteristics (Level 2 Moderators)

#### 3.2.1. Participant Health Status

Baron-Cohen et al. (2015) investigated whether gender differences on the RMET varied between individuals with autism spectrum disorder versus nonclinical individuals. Based on large samples of adults, those authors found essentially no gender difference for participants with autism (*r* = −0.02) and a substantial difference favoring females for neurotypical participants (*r* = 0.24).

To assess whether effect sizes differed by participant health status in the present database, the seven health status categories that each included more than 10 effect sizes (nonclinical, cognitively impaired, physical illness or condition, neurodevelopmental disorders, psychosis, affective disorder, and other mental/behavioral diagnoses) were added as a moderator variable to the weighted random-effects MLM described in a previous section.

Health status significantly moderated effect sizes (QM(*df* = 6) = 45.22, *p* < .001). As displayed in Table 4, four groups (nonclinical, physical illness or condition, psychosis, and other mental/behavioral diagnoses) showed a statistically significant gender difference favoring females, whereas there was no significant gender difference in samples with cognitive impairments as well as affective or neurodevelopmental disorders. When comparing each clinical group to the nonclinical samples (which was the largest category), the gender difference was significantly smaller among patients with cognitive impairments (*r* difference = −0.09, *p* = .003), psychosis (*r* difference = −0.09, *p* < .001), and affective disorders (*r* difference = −0.08, *p* = .005).

Given the heterogeneity of gender differences among the different health status groups and the possibility that test features and other sample characteristics may be confounded with participant health status, all following moderator analyses were run twice, once without and once with participant health status as a covariate. In all analyses, moderator variables were added to the original weighted random-effects MLM described earlier.

#### 3.2.2. Study Location

Kirkland et al.’s (2013) meta-analysis found no significant difference on the RMET for results from the UK versus other countries. Some individual sources in our database reported location results without performing formal tests. Merten (2005) administered a facial expression task in 12 countries and the USA, finding 92% of the countries to show female directional advantage. Greenberg et al. (2022) administered the RMET in 56 countries plus the USA, finding directional female advantage in 98% of them. Finally, the Profile of Nonverbal Sensitivity (PONS, full 220-item version) was administered to high school and/or adult nonclinical samples in 10 non-USA countries, two mixed international groups, and the USA for a total of 112 studies (shown in Figure 7.1 of Rosenthal et al. (1979)). Among these studies, females outscored males directionally in 86% of studies (excluding one with *r* = 0.00). (Few of these 112 effect sizes were included in the present meta-analysis because sample sizes were not reported in Rosenthal et al. (1979)’s Figure 7.1, meaning we could not determine which samples had enough males and females to meet our inclusion criterion.) Thus, the literature shows near universality for the direction of the gender difference.

In the present database, nine countries/regions that each had at least 10 effect sizes were compared (eight countries/regions and the code for diverse countries) and are shown in Table 4. Location did not significantly moderate effect size (QM(*df* = 8) = 13.21, *p* = .105). Post-hoc comparisons with the USA as the reference category (chosen as it had the highest overall estimate of 0.13 and was the largest category) revealed that only studies from the UK and Ireland statistically differed from the USA, showing a slightly smaller effect size (*r* difference = −0.04; *p* = .006). A potential reason may be that some of the specific tests that yielded larger effect sizes (see below), such as the PONS and the GERT, had been predominantly used in the USA and were not present among the UK and Ireland studies. The results remained virtually unchanged when adding participant health status as a control variable (see Table 4). Overall, it can be concluded that the female advantage is present without wide variation across the nine locations.

#### 3.2.3. Participant Age

Neither Hall (1978), McClure (2000), nor Thompson and Voyer (2014) found a significant linear effect of participant age on the gender difference (in McClure’s case, for child and adolescent studies beyond infancy), although differences in the age ranges and other features within each of these meta-analyses make comparing and interpreting these findings difficult. Thompson and Voyer (2014) found a curvilinear effect, such that adolescents and young adults showed a bigger gender effect than children and older adults. Although not a meta-analysis, Greenberg et al.’s (2022) very large study of the RMET in 57 countries showed a fairly stable gender difference across a wide range of ages.

In the present dataset spanning mean ages of 8 to 87, linear and curvilinear effects of sample age as a moderator were assessed by including a linear and a quadratic term (based on mean-centered sample age) into the weighted random-effects MLM. At the mean sample age of 30.54 years, *r* was 0.11 (*p* < .001; 95% CI [0.100; 121]). The linear term was significant though modest (*β* = −0.001; *p* < .001; 95% CI [−0.002; −0.001]), indicating that with each additional year of age, the gender difference effect size decreased by 0.001. The quadratic term was not significant, suggesting no evidence of a curvilinear relationship between sample age and gender differences. These results remained virtually unchanged when adding participant health status as a control variable (see Table 4).

Because the quadratic analysis does not map directly onto the four age groups we had coded, we also looked at that contrast. When effect sizes were grouped by mean age into four groups (8–12, 13–17, 18–27, 28 or older/mix of 18–27 and older), a moderator analysis was significant (QM(*df* = 3) = 50.87, *p* < .001); see Table 4 for the effect size per group. When “8–12” was used as the reference group in pairwise comparisons, effect sizes for the “13–17” (*r* difference = 0.09, *p* < .001) and “18–27” (*r* difference = 0.05, *p* < .001) categories were significantly higher, while the effect size for the “28 or older/mix of 18–27 and older” category (*r* difference = 0.01, *p* = .613) was not significantly different from the “8–12” category. The values and group differences remained virtually unchanged when participant health status was controlled (see Table 4). These findings support the curvilinear trend found by Thompson and Voyer (2014).

#### 3.2.4. Participant Race

Participant race was coded based on the majority representation within the sample. Specifically, if 60% or more of the participants belonged to a particular racial group, the entire sample was coded as belonging to that category. If no single racial group comprised more than 60% of the sample, race was coded as mixed. Racial composition was only known for 471 (39.6%) of the effect sizes. Only the codes White, East and Southeast Asia, Black in USA, and mixed had more than 10 studies and these were assessed as a moderator of effect size. Sample race moderated the gender difference (QM(*df* = 3) = 9.90, *p* = .020), although all four groups still show a significant effect favoring females (see Table 4). Studies coded as “mixed” yielded lower effects compared to studies with predominantly White participants (*r* difference = −0.05, *p* = .005). This result remained unchanged when controlling for sample health status (*r* difference = −0.04, *p* = .019; see also Table 4).

#### 3.2.5. First Author Gender

Eagly and Carli (1981) analyzed the effect sizes from Hall’s (1978) meta-analysis, finding that a higher proportion of male authors was associated with a smaller gender difference in accuracy. This question was examined in Hall’s (1984) and McClure’s (2000) meta-analyses and there was a nonsignificant, opposite-direction result in both.

In the present meta-analysis, first author gender could be coded based on the first name for 1164 (98%) of the effect sizes; 483 (41.5%) of authors were male and 681 (58.5%) were female. The test of moderation was not significant (QM(*df* = 1) = 2.57, *p* = .109; see Table 4). When controlling for sample health status, effect sizes were significantly higher for female compared to male first authors, but with a very small effect (*r* difference = 0.02, *p* = .048).

#### 3.2.6. Year

Hall’s (1978) meta-analysis found a correlation of 0.28 (*p* < .10) between the gender-difference effect size and publication year (ranging from 1923 to 1978). In Thompson and Voyer’s (2014) meta-analysis, where the years ranged from 1929 to 2012, there was no significant relation for year. In the present dataset, the weighted random-effects MLM with source year as a moderator showed that at the mean publication year of 2013, *r* was 0.12 (*p* < .001; 95% CI [0.111; 0.127]) and the year trend was significant, *β* = −0.002 (95% CI [−0.002; −0.001]); with each additional year, *r* decreased by 0.002, which can be considered very small.

In the present database, later source year was significantly associated with many variables: female first authorship, older age of participants, sample having a clinical diagnosis, fewer items on the test, and more static stimuli, as well as results being provided by authors upon our request (by design, these were all from 2015 or later). Also, different tests were used in different years. To account for these possible confoundings, the weighted random-effects MLM was repeated with sample age and number of items (both median-centered) as well as first author gender, dynamism, participant health status, test name (where tests with 20 or fewer effect sizes were summarized into one category), and publication status as control variables. The *β* for source year remained similar (*β* = −0.003, *p* = .004).

### 3.3. Test Characteristics (Level 1 Moderators)

#### 3.3.1. Number of Items

The number of items in the test ranged from 4 to 399. Due to its skewed distribution, the number of items was centered around the median. The test of moderation was significant (QM(*df* = 1) = 9.96, *p* = .002), showing that at the median of 36 items, the gender difference was *r* = 0.12 (*p* < .001). With each additional item, *r* increased by 0.0002 (95% CI [0.0001; 0.0004]), which can be considered a very small effect.

#### 3.3.2. Cue Channels

Hall (1978) and Thompson and Voyer (2014) found bigger gender effects for tests that combined cue channels. Comparisons between visual and auditory cues were inconsistent: the gender difference was bigger for visual than auditory cues in both Hall (1978) and Hall (1984) but not in Thompson and Voyer (2014), and in Hall et al. (2016), auditory cues showed a bigger effect than visual cues.

Table 5 presents gender differences for all cue channels in the present database for which at least 10 effect sizes were available (face, eyes, body and hands, masked voice, video with unmasked voice, video with masked voice, and multichannel total). Masked voice refers to stimuli in which words are not present (only vocal sounds), or words that are present but cannot be understood or are not relevant to the affect being judged. The multichannel total code was given when the researcher scored accuracy for two or more cue channels and then combined them into a total score—for example, face items and separate voice items combined into a total. Cue channel did not significantly moderate the gender difference (QM(*df* = 6) = 7.00, *p* = .321), but post-hoc tests revealed that compared to the reference category with the highest effect size (multichannel total: *r* = 0.15), tests including only the face and only the eyes yielded slightly smaller effects (face: *r* difference = −0.03, *p* = .033; eyes: *r* difference = −0.04, *p* = .030). Given that multichannel totals had notably more test items (*M* = 126) than did single channels (*M* = 48), we repeated the analysis controlling for the number of items. The results remained largely unchanged, with tests including multiple cue channels showing a slightly larger effect than tests including only the face (*r* difference = −0.04, *p* = .021), only the eyes (*r* difference = −0.04, *p* = .033), or tests with video and unmasked voice (*r* difference = −0.06, *p* = .029). Effect sizes for all cue channels also remained similar when controlling for participant health status (see Table 5). Overall, the gender difference was relatively consistent across cue channels although the multichannel total was the largest effect, similar to the previous meta-analyses alluded to above.^1^

#### 3.3.3. Specific Tests

Codes for named tests refer to the stimulus set, which may be subject to many variations in practice. For example, the test developers may offer shorter and longer versions, and individual investigators may edit or alter the stimuli (for example, by morphing) or choose the number of stimuli they wish to use. As an extreme example of the latter, PONS stimuli can be administered as a 20-item single-cue test (e.g., face only) or as the full 220-item test that involves single channels (face, body, or voice) as well as many combinations (such as full person plus voice). Indeed, some of what we call tests were not developed and validated as such, but only as stimulus sets with verified expressions for several affective states. Examples are the Japanese and Caucasian Facial Expressions of Emotion (JACFEE (Biehl et al. 1997)) and the POFA collection (Ekman and Friesen 1976), both of which contain prototypical basic emotional expressions from which researchers then choose which stimuli to use and how they might alter them for their own purposes. Therefore, any conclusions about tests are only approximate because of wide variation in specific applications and conclusions are, also, not independent of the preceding analyses of cue channels because tests differ in what cues they present.

Table 5 shows results for eight named tests that each had at least 20 effect sizes, as well as for the categories “combination of two or more tests” and “other tests” (which includes all tests that were used fewer than 20 times and custom-made tests for single studies). One can see from Appendix A that most of the tests used posed facial expressions of basic emotions. Several tests are more complex, involving video and/or multiple cue channels, for example, the GERT and the PONS.

Effect sizes differed by test (QM(*df* = 9) = 31.46, *p* < .001), but were significantly above zero for all test categories (see Table 5). When comparing each test against the reference category “other tests” (which was the largest category and close to the overall effect size), the Diagnostic Analysis of Nonverbal Accuracy (DANVA (Nowicki and Duke 1994)) adult faces test, GERT, and PONS showed a significantly larger effect size (DANVA: *r* difference = 0.04, *p* = .046; GERT: *r* difference = 0.05, *p* = .009; PONS: *r* difference = .05, *p* = .005). Conversely, the Penn ER40 test had a smaller effect size compared to the “other tests” category (*r* difference = −0.04, *p* = .014). When controlling for participant health status, effect sizes remained similar (see Table 5). Compared to the “other tests” category, effect sizes for GERT, PONS, and ER40 were still significantly higher or lower, respectively, but for DANVA, the difference was not significant anymore. Instead, the POFA now yielded a significantly larger gender difference (*r* difference = 0.03, *p* = .024). Thus, gender differences are sometimes larger when stimuli are more complex. Overall, however, the differences in effect size between tests are relatively small.

#### 3.3.4. Stimulus Presentation Mode

For stimuli containing visual cues, we coded whether stimuli were presented in a static or dynamic format. Audio-only tests were not included in this analysis because they are dynamic by definition. Most effect sizes were based on static stimuli (*k* = 881 or 74.2% of non-missing values), while 210 (17.7%) were based on dynamic stimuli. Presentation mode significantly moderated the gender difference (QM(*df* = 1) = 4.46, *p* = .034), with dynamic stimuli yielding larger effects than static stimuli (*r* difference = 0.02, *p* = .032, see Table 5). The difference between static and dynamic stimuli remained significant when controlling for participant health status (*r* difference = 0.02, *p* = .046). These results are in line with the finding that the GERT and PONS tests—two tests using dynamic stimuli—yielded some of the highest average effects.

#### 3.3.5. Stimulus Creation Mode

The distinction between posed and spontaneous target behavior was found to be nonsignificant in Thompson and Voyer’s (2014) meta-analysis. In the present database, information on stimulus creation mode was available for 806 effect sizes (67.8%). Of these, 87.2% were derived from posed expressions, deliberately enacted for the purpose of stimulus creation, while 12.8% were based on spontaneous behavior, recorded under relatively unconstrained conditions, such as during the recounting of an emotional experience. Creation mode did not significantly moderate effect size (QM(*df* = 1) = 0.21, *p* = .649). This result did not change when controlling for participant health status (*r* difference = −0.01, *p* = .289).

#### 3.3.6. Target Gender

Hall (1978) found no effect for target gender (consistent with cited within-study analyses), while Thompson and Voyer (2014) found that the gender difference was largest for male targets. In the present database, most effect sizes were for tests that included both female and male targets (*k* = 1001, 83.3%), while 90 effect sizes (7.6%) were based on female targets only and 26 effect sizes (2.2%) were based on male targets only. The test of moderation was not significant (QM(*df* = 2) = 4.50, *p* = .105). Likewise, when controlling for participant health status, the two categories “male” and “female” did not differ from the reference category “female and male” (male: *r* difference = −0.03, *p* = .278; female: *r* difference = 0.01, *p* = .305; see Table 5).

#### 3.3.7. Target Age

Target age was not a significant moderator in either the Hall (1978), McClure (2000), or Thompson and Voyer (2014) meta-analyses. In the present database, most effect sizes were obtained with tests featuring adult targets (*k* = 1132, 95.3%), while some studies included children (*k* = 26, 2.2%) or both children and adults (*k* = 11, 1%). Target age did not significantly moderate effect size (QM(*df* = 2) = 1.56, *p* = .458). When controlling for participant health status, compared to adult targets, the other two groups still did not show a significant difference (child: *r* difference = 0.02, *p* = .311; child and adult: *r* difference = 0.03, *p* = .575; see Table 5).

#### 3.3.8. Target Race/Ethnicity

Most effect sizes were obtained with White targets (*k* = 767; 64.6%), while 33 effect sizes were based on tests with East Asian targets (2.8%), and 174 effects came from tests with targets of different racial/ethnic groups (14.6%). Fewer than 10 studies included only Black, Latinx, South Asian, or Middle Eastern targets; and for 205 effect sizes (17.3%), target ethnicity was unknown. Target ethnicity for the three categories with ten or more studies moderated effect size (QM(*df* = 2) = 7.15, *p* = .028), with tests including targets of different racial/ethnic groups yielding smaller effects compared to tests with only White targets (*r* difference = −0.02, *p* = .008; see Table 5), and East Asian targets not showing a significant difference. This result remained unchanged when controlling for participant health status (*r* difference for mix of ethnicities compared to only White targets = −0.02, *p* = .017).

#### 3.3.9. Authors’ Analysis Model

Most often, effect sizes were based on means and standard deviations or *t*-tests (*k* = 1104; 92.9% of all effect sizes), meaning study authors had not statistically controlled for any other factors. However, in 67 instances (5.6%), the effect size came from a multi-way ANOVA or another analysis that included covariates, an approach that could produce a larger effect size due to the reduction in otherwise unexplained variance, or it could produce a smaller effect size if, in fact, controlling for covariates removed the impact of confounders. Authors’ analysis model was a significant moderator (QM(*df* = 1) = 33.21, *p* < .001), with effects resulting from analyses including covariates being larger (*r* difference = 0.09, *p* < .001; see Table 5), also when controlling for participant health status (*r* difference = 0.08, *p* < .001). If the adjusted effect sizes are removed, the overall effect (estimated with the weighted random-effects MLM) fell only a trivial amount, from *r* = 0.12 to *r* = 0.11 (95% CI [0.10; 0.12]).

### 3.4. Publication Bias

#### 3.4.1. Publication Status

Looking at the publication status of sources provides one window into the possibility of publication bias. Neither McClure’s (2000) nor Kirkland et al.’s (2013) meta-analyses found a significant difference between published and unpublished studies.

In a first step, we assessed whether the results we found in published articles differed from master’s theses/dissertations, unpublished studies, chapters/books, and effect sizes sent on request to Kirkland et al. (2013). The omnibus test (QM(*df* = 4) = 3.58, *p* = .465) as well as the post-hoc comparisons with “published” as the reference category were not significant. In a second step, we compared effects we retrieved directly from studies published in 2015 or later (*k* = 257) with effects sent by authors upon our request for those same years (*k* = 583). The published effects in this comparison yielded higher effect sizes than the effects obtained upon request (*r* = 0.12 vs. *r* = 0.10; *r* difference = −0.02; *p* = .018), but the difference was very small. Table 4 shows the meta-analytic effect sizes for all publication status categories regardless of year.

#### 3.4.2. Other Publication Bias Procedures

Additionally, we employed three procedures recommended by Rodgers and Pustejovsky (2021) to assess selective reporting. As described below, selective reporting could have occurred both in published and unpublished studies; we therefore conducted these analyses with the whole database (*k* = 1188). Firstly, using a step function selection model (Viechtbauer 2010), we examined whether effect sizes with *p* values greater than the common threshold of *p* > .05 were less likely to be present in the dataset, which could suggest selective reporting in the sense that researchers omitted nonsignificant results within primary studies or when deciding what results to send to us. Given that selection models cannot currently account for nested data structures, this analysis was conducted on a dataset where effect sizes were aggregated within the 1011 studies, following the recommendations of Rodgers and Pustejovsky (2021). We first fitted a weighted random-effects model, which assumes that studies are equally likely to be present in the dataset regardless of their *p* values, and then compared it to the selection model, which estimates separate probabilities for predefined *p* value ranges (here, *p* ≤ 0.05, .05 < *p* ≤ .10, and .10 < *p* ≤ .20, as outlined in (Viechtbauer 2010)). The likelihood ratio test for the selection model parameters was not significant (LRT(*df* = 3) = 6.79, *p* = .079), indicating that the selection model did not provide a significantly better fit than the weighted random-effects model and thus not strongly supportive of selective reporting or publication bias. However, the results showed that studies with .05 < *p* ≤ .10 were slightly less likely (0.80 times) to be present in our dataset compared to those with *p* ≤ .05; studies with .10 < *p* ≤ .20 were 0.75 times as likely; and studies with *p* > .20 were 0.76 times as likely to be present. A more conservative test, using a *p* value of .025 as the step in the selection model (known as the “three-parameter selection model” (3PSM (Pustejovsky and Rodgers 2019)), was significant (LRT(*df* = 1) = 4.94, *p* = .026), indicating that effect sizes with a *p* value > .025 were 0.79 times as likely to be included as those with a *p* value ≤ .025. In both selection models, the estimated true average effect size was *r* = 0.11, which is nearly identical to the overall effect size obtained with and without MLM (see above).

Secondly, we used the Egger MLMA (Mixed-effects Linear Model Analysis) approach in the metafor R package, which is an adaptation of the traditional Egger’s test to the nested structure of the present dataset. While selection models such as the 3PSM described above assess whether studies with less significant *p* values are underrepresented in the database, the Egger MLMA examines whether smaller or less precise studies produced larger effects (“small study effects”) by testing whether effect sizes are linked to their standard errors. In our database, small study effects may, for example, have occurred if studies with a low *N* were more likely to be published (or sent to us by authors) if effects were large. The analysis yielded no significant relationship between the standard errors and the corresponding effect sizes (moderator test result: QM(*df* = 1) = 0.06, *p* = .812), suggesting that smaller, less precise studies did not contribute disproportionately to larger effect sizes. Across the entire dataset, the correlation between sample size and effect size was *r*(1186) = −0.01 (0.00 for clinical samples, −0.02 for nonclinical samples), which speaks not only to a lack of bias but also to the remarkable consistency of the gender effects.

Thirdly, we applied the Egger Sandwich method, which extends the traditional Egger’s test by using Robust Variance Estimation (RVE) to compute adjusted standard errors that are robust to the nested structure of the dataset. This was performed using the “robust” function in metafor for using cluster-robust inference (variance–covariance estimator CR1). The results similarly indicated no significant relationship between effect sizes and their standard errors (*F*(*df*1 = 1, *df*2 = 1009) = 0.06, *p* = 0.807), further supporting the absence of small study effects.

Taken together, our analyses showed some evidence for publication bias and/or selective reporting, in that effect sizes sent by authors upon our request were slightly smaller than published results for the same years, and effect sizes with *p* values exceeding the common significance threshold were slightly underrepresented in the database. These effects were not strong and their detection was, of course, enabled by the large size of the dataset. We conclude that evidence for publication bias is weak.

## 4. Discussion

This meta-analysis of gender differences in decoding affect cues from recorded stimuli builds on several earlier meta-analyses that addressed the same question with much smaller and earlier databases as well as varying inclusion criteria and analysis models. We were able to confirm previous conclusions that women and girls are more accurate than men and boys. The overall effect size was *r* = 0.12 (*d* = 0.24), with 84% of all non-zero differences favoring females. This effect size is smaller than in some of the previous meta-analyses (Table 1), but it is not directly comparable to any of them because of methodological differences including tests, years, age ranges, and other variables. Nevertheless, the overall conclusion is the same.

With any effect size finding, an obvious question is “compared to what”? Effect sizes can hardly ever have meaning in isolation from their network of other correlates. For any given gender difference, this network extends in two directions: other correlates of gender, and other correlates of the behavior showing the gender difference. Hall (2006) posed both of these questions with regard to gender differences in accurate cue decoding, based on a compilation of relevant effects from the literature. For other correlates of gender, across social personality and cognitive variables compiled in meta-analyses, Hall found the median gender difference to be *r* = 0.11, nearly identical to Richard et al.’s (2003) review of only social psychological variables found in meta-analyses (*r* = 0.12), and to Zell et al.’s (2015) analysis of meta-analyses of gender differences in multiple domains (*r* = 0.10). These values are similar in magnitude to the gender differences found with intelligence test batteries as described in the Introduction (e.g., Giofrè et al. 2022; Longman et al. 2007, Cohen’s d values between 0.20 and 0.30 corresponding to correlations between 0.10 and 0.15). Thus, the gender difference in affect cue decoding is much like the gender difference for other single psychological traits, although gender differences for broader combinations of traits (e.g., clusters of personality traits more typical of women or men) tend to be larger (e.g., Del Giudice et al. 2012; Eagly and Revelle 2022). For the second “compared to what?” question regarding the magnitude of other correlates of cue decoding (besides gender), Hall (2006) located 112 individual studies and calculated a median correlation of 0.18.

This exercise in answering the “compared to what” question gives context in which to appraise the present gender effect of *r* = 0.12 (*d* = 0.24). We conclude that the gender effect we obtained is much like that for other psychological correlates of gender and somewhat smaller than other correlates of cue decoding accuracy. A similar advantage for females is also usually found for other components of ability emotional intelligence besides accurate affect decoding, in particular, emotion understanding and the ability to manage other people’s emotions (Hampel et al. 2024; Schlegel and Mortillaro 2019).

The gender effect in the present study did not vary significantly across nine countries/regions of the world as well as across several study characteristics (author gender, cue channels, posed versus spontaneously elicited expressions, target gender, target age). Even when moderators were statistically significant (which could happen, in part, due to the statistical power afforded by the large database), the differences were often very small, attesting to the impressive consistency of the gender effect.

Some of the moderators we examined had been included in previous meta-analyses, such as perceiver age, target age and gender, and cue modality. While the present moderator results were similar to some of those found in previous meta-analyses, some were not (and previous meta-analyses’ moderator results did not always agree with each other). Such discrepancies are hard to interpret because previous meta-analyses were much smaller than the present one and, again, were different methodologically from each other and from the present meta-analysis in a number of ways.

This is the first meta-analysis on the topic to compare clinical and nonclinical samples. We found nonsignificant gender differences (although directionally still favoring females) for neurodevelopmental disorders, cognitive impairments, and affective disorders; for psychosis, although the gender difference was significant because of the larger number of studies, the effect was very similar in size to the just-named groups (around *r* = 0.04). Baron-Cohen et al. (2015) foreshadowed these findings in a study that compared the gender difference on the RMET between nonclinical participants and those with a diagnosis on the autism spectrum, finding that the nonclinical sample showed a notable gender effect while the autism sample showed none.

In our data, the clinical group with an effect size most similar to that of the nonclinical group was people with physical conditions, suggesting that it is psychological disorder that suppresses a gender difference, not disability or illness in general and not something unique to autism. Many studies show that performance on emotion recognition tasks is depressed in psychologically diagnosed groups (Cotter et al. 2018), but scoring lower does not in itself have implications for the gender difference. Instead, it is possible that having a psychological disorder impairs performance for women more than for men, as indeed was the case in Baron-Cohen et al. (2015). According to Geary’s (2021) evolutionary model, one explanation might be that women’s advantage in affect decoding arises from their stronger reliance on condition-dependent neural and cognitive systems, which evolved under greater evolutionary pressures to navigate complex social dynamics. Such systems are particularly sensitive to disruption under suboptimal physiological conditions, such as hormonal imbalances, neuroinflammation, or reduced neural efficiency associated with psychological disorders. Consequently, psychological disorders may impair women’s performance more significantly than men’s, narrowing the gender difference in decoding accuracy. Because we found clinical status to moderate effect sizes, we calculated all other moderator analyses both controlling for health status and not controlling for it. With few exceptions, controlling for health status did not alter the general pattern of moderator effects.

Age showed linearly declining gender effects (older samples’ effects being smaller than younger samples’). Although a traditional polynomial quadratic contrast was not significant, a contrast specifying specific age groups did show a significant quadratic trend, with the gender difference being larger in the age range of 18 to 27 than in younger or older groups. This is similar to a result in the Thompson and Voyer (2014) meta-analysis.

Samples that were over 60% White showed a bigger gender difference than samples for which no group had representation over 60%. Caution is needed when interpreting this finding, as we often lacked specific information on the racial make-up of these groups and most were from the United States. More studies investigating the role of race, ethnicity, and culture on accuracy in judging affective states is needed, but the lack of significant differences between primarily White samples and samples that were primarily Asian or Black indicates we do not have a strong reason to expect differences due to race, ethnicity, or culture.

Year was a significant moderator, with later years showing very slightly smaller differences even controlling for possible confounds. Unfortunately, it is difficult to interpret year as a moderator because we do not know if males are getting better or females are getting worse (or both at once). Such an analysis would be important; ideally, one would hope that males are getting better rather than that females are getting worse. However, such an analysis would need to be based on an identical test that is administered to comparable samples across a sufficient number of years to make a strong inference about the impact of year (versus other confounding factors) on males’ and females’ separate accuracy trajectories. In the present database, these conditions could not be met. For instance, three of the eight tests that have been used at least 20 times (see Table 5)—TASIT, ER40, and GERT—have only been implemented since 2010 or later. Even for the older tests like JACFEE, DANVA, and Ekman Faces (POFA), most effect sizes in the database originate from the last 10 to 15 years. Furthermore, these tests have been administered in various forms and versions, making direct comparisons challenging. For example, although the PONS has been used (albeit with decreasing frequency) since 1979, it has been administered in different modalities—sometimes only audio, sometimes only face or body—and also in different short versions. Moving forward, in addition to consistently using established tests over a longer time period, we hope future authors will report their descriptive gender results so that male and female trajectories can be tracked over time.

Although, overall, there was not a significant moderating effect of cue channels, there emerged interesting patterns that intersect with results for specific tests. Tests of single cues, mainly face only and eyes only, had smaller effects than tests that involved more cue channels or were more complex in other ways. Among cue channels, the multichannel cue total had the largest effect. Among specific tests, the Awareness of Social Inference Test-Emotion Evaluation Test (TASIT-EET (McDonald et al. 2006)) and GERT had relatively large effects and both of these show videos that simultaneously present multiple cue channels. Most versions of the PONS, which also had a relatively large effect, are video with either simultaneously presented multiple cue channels or multiple single channels that are totaled. The category for two or more tests combined, which included combinations of different cues and also combinations of the same single-cue tests that varied in various ways, also had a relatively large effect. Finding that the POFA—a set of prototypical facial expressions—also had a relatively large effect may seem inconsistent with this pattern. However, in many studies the POFA stimuli were subjected to morphing and other manipulations that added a measure of complexity. Another possible exception to a trend for more complex stimuli to have somewhat larger effects is the DANVA adult faces test, which had a relatively large effect while being a single-channel test. However, the DANVA expressions are not prototypical and they vary in intensity, making for a more complex task. Although these interpretations are speculative, we believe there is some evidence that tests with more subtle and/or complex stimuli are more likely to produce a larger gender effect. This fits with some findings in the literature (Hoffmann et al. 2010; Sasson et al. 2010).

Finally, the small number of studies that controlled statistically for other sources of variance had a gender effect that was twice as big as that for uncontrolled studies. Finding that reducing random error increases effect size is not a surprising result from a statistical standpoint, but it raises the question of which estimate one should “take home”. There is (and was, in our database) no specific covariate adjustment that one can say yields the most “correct” estimate because different studies controlled for different variables. On the other hand, an unadjusted estimate does not necessarily capture a more pure or truer estimate because different samples will differ from each other in many ways. Importantly, some researchers may proactively reduce measurement error by holding population (e.g., sample age range) and situational variables (e.g., testing circumstances) constant, which could increase the effect size even in the absence of post-hoc covariate adjustments. Thus, the difference between unadjusted and adjusted effects is more a matter of degree than of kind. There is no consensus on how covariate-adjusted effects should be handled in meta-analysis (Aloe et al. 2016). Certainly, most meta-analyses likely do not take account of this methodological difference. While the adjusted effects were bigger than unadjusted effects, due to their small number they had only a very minor effect on the overall effect (reducing it from *r* = 0.12 to *r* = 0.11). For this reason, we retained the adjusted effects in the database. Including this as a moderator is instructive and would be a good addition to standard meta-analytic practice.

Turning to analyses of publication bias, several procedures were applied that suggested some, but not strong, evidence of overall publication bias. For instance, effect sizes from studies that were sent by authors upon request were slightly smaller than effect sizes from published studies during the same time period, although they still favored females. It is hard to interpret this because we do not know if there were biases in what authors chose to send.

### Origins of the Gender Difference

The uniformity in our findings that females are more accurate in decoding affect cues than males reinforces previous findings, but is not helpful in disentangling why the advantage exists. Like most gender differences, the origin of this specific gender difference is likely sociocultural in nature. In the following section, we will mention four different hypotheses for the existence of these gender differences. It should be noted that while these hypotheses are distinct, they are not mutually exclusive and overlap significantly.

The evolutionary hypothesis holds that evolution creates inborn mechanisms that determine, or predispose, females to excel in decoding affect cues, adaptations that might optimize mate selection or the survival of offspring (Hampson et al. 2021). For example, Haselton and Buss (2000) hypothesize that since women are more at risk in mating situations because they are responsible for carrying and caring for young children, they have evolved to be more accurate in reading the intentions of potential mates. Also, in line with evolutionary theories, Geary (2019) proposed that female–female competition, particularly through relational aggression involving gossip and reputation management, may have driven the evolution of women’s heightened sensitivity to nonverbal cues and facial expressions, enabling them to better navigate social dynamics and defend against such aggression.

There are arguments that gender differences are learned behaviors. The power hypothesis holds that females acquire skills that are adaptive for their lower status in relationships and society in general (LaFrance and Henley 1994). Under the power hypothesis, women would learn to be better decoders of affective states in order to be more successful in their lower status position.

The social roles hypothesis holds that the social roles that women typically and historically find themselves in (wife–mother versus breadwinner, in helping–facilitating occupations versus leadership occupations, etc.) establish expectations that are conveyed to females starting very early in life (Eagly and Wood 1999). The power and social roles hypotheses can be linked through the observation that women’s social roles are, typically, lower power roles, and through the possibility that given the history, gender stereotypes and expectations may linger even when overt power differentials are weakened or even eliminated. The social roles hypothesis also has its links to biology, insofar as biology (pregnancy and lactation, physical size) has historically contributed to social structures and roles (Wood and Eagly 2002). Within social role theory, this skill could have been developed in women’s roles as mothers who had to care for young children who were not yet verbal. Developing better skills for understanding the needs of their preverbal children could be beneficial in ensuring the survival of their offspring, and once these skills were developed, they were modeled as important to girls who would become mothers in adulthood.

Finally, the motivation hypothesis holds that males and females have essentially the same skill repertoires and that the performance differences emerge only because women know they should be good at cue reading and men know they are not expected to be—therefore, when tested, the characteristic gender difference emerges because one gender tries harder, or less hard, than the other (Ickes et al. 2000). However, women actually possess more knowledge about nonverbal communication’s cues and usages than men do (e.g., Ogawa and Hall 2022), undermining the hypothesis that accuracy is the result of in-the-moment effort applied or withdrawn.

All of the above listed hypotheses are intertwined in one way or another. Expectations embodied in differential motivation-in-the-moment might influence test scores, but those expectations may have arisen from social roles that produce a lifetime of motivation to be emotionally responsive and aware; social roles may arise from power imbalance and, yet farther back, from biological/evolutionary factors. Even a biological predisposition to be interpersonally alert could not develop into specific skills without immersion in cultural norms and socialization (i.e., learning). While research on all of these avenues is valuable, we do not believe a final, or at least a simple, explanatory framework will or can emerge. As society continues to make advances towards gender equality, the power dynamics based on gender will shift, and if there is a continued leveling of roles, we might expect to see the gender difference get smaller. While we saw a small trend towards the gender differences shrinking over time in the current analysis, this relationship was small, and we are unable to draw inferences from it that will provide more concrete answers to what the origins of the differences are.

## 5. Limitations

Despite the large size of the present database, we know that many relevant results must have been missed, considering that this topic has been investigated for nearly a century and that it is pursued with highly varying nomenclature across a number of disciplines, in journals not tracked by PsycInfo. Nevertheless, we strongly believe that our procedures ensured as unbiased a database as possible.

It is a shortcoming of the effect-size approach that it obscures the mean values of the groups being compared—in our case, the mean performances of males and females. This is especially obvious when looking at changes in effect sizes over time, but it applies to all moderator analyses. Within-study examinations, where methodological features can be well controlled and the means for men versus women can be directly compared as a function of other study features (e.g., mental health status, specific tests used, age), provide an additional window into how males’ and females’ performance varies with circumstances. Another limitation is that with the present dataset, it was not possible to examine whether gender differences vary by specific affects. While women outperform men overall, the meta-analysis of Thompson and Voyer (2014) found a bigger gender difference for negative than positive emotions. Some studies suggest men may be better at recognizing anger and aggression, and women at fear and sadness—patterns possibly shaped by evolutionary roles in threat detection and caregiving (Kret and De Gelder 2012). However, Thompson and Voyer (2014) did not find that pattern.

As was noted in the Introduction, how people talk about gender and the acceptance around diverse gender identities has shifted greatly in the last decade. How psychologists are measuring gender has also changed as they are allowing people to self-report outside of a gender binary. The present meta-analysis could deal only with a traditional gender binary. Adding additional gender categories and measuring different aspects of the perceiver gender roles as moderators could be potential avenues for disambiguating when females are better at decoding affective states then males. While the current study is limited by the way past researchers have measured gender, we hope that the continued robust, though modest advantage of females in decoding affective states points to gender being an important variable to quantify and analyze when investigating this skill.

Although the present study does not address outcomes of this difference, an important question for future research is whether accuracy in judging affect cues impacts outcomes (e.g., job performance, relationships) differently for girls/women and boys/men. Maybe this skill is more strongly correlated with outcomes for women whereas men can “get away with” not being as accurate and still achieve the same outcomes whenever those outcomes depend on the judgments of other people (e.g., supervisors, friends, voters). If this is the case, it may be a mechanism that maintains the gender difference in accuracy (women benefit more from being accurate, so they might practice that skill more).

## 6. Conclusions

Girls and women outscore boys and men on tests of judging affect cues. Even though significant moderators were uncovered, in every subgroup of the various moderators, females outscored males, even in clinical groups where the effects were very small. Thus, the best generalization to be taken from this work is that the gender effect in decoding affect cues is remarkably robust across time, locations, age groups, and several test characteristics and is markedly bigger in better-controlled comparisons.

## Figures and Tables

**Table 1:** Summary of meta-analyses of gender effects in decoding recorded audiovisual cues.

Study	*M* Effect Size ^a^ (Number of Effects)	Percentage Showing Female Advantage ^b^	Instruments	Moderators
Hall (1978)	0.20 (46)	84%	Assorted	Cue channels, perceiver age, publication year, sample size, target age, target gender
Rosenthal et al. (1979)	0.20 (133)	80%	PONS ^c^	None
Hall (1984)	0.25 (18)	81%	Assorted	Cue channels, first author gender
McClure (2000)	0.09 (60)	n/r	Assorted	First author gender, instrument or measurement technique, perceiver age, publication status, target age
Kirkland et al. (2013)	0.09 (42)	n/r	RMET ^d^	Language of test, perceiver country, publication status, researcher group
Thompson and Voyer (2014)	0.14 (404)	n/r	Assorted	Cue channels, emotion, emotion type, instrument, measurement, target age, target gender, perceiver age, presentation method, posed/spontaneous, publication year
Hall et al. (2016)	0.22 (37)	92%	Assorted	Cue channels

Note: n/r = not reported. For McClure (2000), only child and adolescent studies are included (no infant studies). ^a^ Effect size is the point biserial (Pearson) correlation. Positive values mean females scored higher; negative values mean males scored higher. ^b^ Direction of the gender difference, irrespective of *p* value. ^c^ Profile of Nonverbal Sensitivity (Rosenthal et al. 1979). ^d^ Reading the Mind in the Eyes Test (Baron-Cohen et al. 2001).

**Table 2:** Study descriptions.

Variable	Description
**Study moderator variables**	
Participant health	Nonclinical (83.6%); clinical: psychosis (6.4%); physical illness/disability/injury (2.6%); cognitive impairment (2.4%); affective disorders (2.2%); neurodevelopmental disorders (1.4%); other (1.5%)
Location (*k* = 1003)	United States (36.9%); non-Anglophone Europe (29.1%); United Kingdom and Ireland (9.4%); East and Southeast Asia (5.9%); Australia and New Zealand (5.8%); diverse countries (4.5%); Canada (2.8%); Central and South America and Mexico (2.7%); Middle East (1.6%); other locations (1.4%)
Participant age (*k* = 888)	*M* = 30.55 (*SD* = 14.87), *Md* = 27, range = 8–87
Participant age group (*k* = 1007)	8–12 (6.4%); 13–17 (5.8%); 18–27 (43.1%); 28 and up or combination of this and preceding category (44.8%)
Participant race (*k* = 376)	White (62.8%); East or Southeast Asian (16.2%); mix of two or more of races (<60% of a named group: 16.0%); African-American (2.7%); other (2.4%)
First author gender (*k* = 991)	Male (43.7%); female (56.3%)
Year of publication	*M* = 2014, *Md* = 2018, range = 1931–2023
**Other descriptives**	
Study origin	PsycInfo search (85.9%); bibliographies (9.5%); serendipity and unpublished from listserves (4.7%)
Search terms	Emotion recognition (46.2%); Reading the Mind in the Eyes Test (25.4%); other tests and categories (28.4%)
Type of source	Results were in article, chapter, or book (47.4%); results were from article and sent by its author on request (46.9%); thesis or dissertation, meta-analysis, or unpublished from listserves (5.8%)
*N* of male participants (*k* = 989)	*M* = 399.15, *Md* = 48, range = 15–142,694
*N* of female participants (*k* = 989)	*M* = 442.85, *Md* = 61, range = 15–148,923
Total *N* of participants	*M* = 828.52, *Md* = 110, range = 30–291,617, grand total *N* = 837,637

Note: The number of studies (*k*) is 1011 unless indicated. See Supplementary Materials File S3 for detailed descriptions of health classifications.

**Table 3:** Test characteristics.

Variable	Description
Number of items (*k* = 1114)	*M* = 50.98 (SD = 51.95), *Md* = 36, range = 4–399
Cue channel (*k* = 1187)	Face only (43.2%); eyes only (31.8%); voice only (content masked or no verbal content) (7.0%); full video, masked voice (6.0%); full video, unmasked voice (5.5%); multichannel total (multiple separately tested cue channels combined in total score) (5.2%); other (1.3%)
Test (*k* = 1187)	Reading the Mind in the Eyes (RMET, 30.5%); Pictures of Facial Affect (POFA, 9.3%); Penn Emotion Recognition Test (ER40, 4.5%); Profile of Nonverbal Sensitivity (PONS, 4.3%); Diagnostic Analysis of Nonverbal Accuracy-Adult Faces (DANVA-AF, 3.8%); Geneva Emotion Recognition Test (GERT, 2.9%); combination of two or more tests (2.9%); Japanese and Caucasian Facial Expressions of Emotion (JACFEE, 2.0%); The Awareness of Social Inference Test (TASIT, 1.9%); others (37.9%)
Stimulus presentation mode (*k* = 1091)	Static or photographs morphed to simulate movement (80.8%); film or video (19.2%) [item not coded for voice-only tests]
Stimulus creation mode (*k* = 806)	Posed (87.2%); spontaneous (12.8%)
Target gender (*k* = 1117)	Male (2.3%); female (8.1%); both (89.6%)
Target age (*k* = 1169)	Children (2.2%); adults (96.8%); both (0.9%)
Target race/ethnicity (*k* = 983)	White (78.0%); mixture (<80% of any named group, 17.7%); East Asian (3.4%); other (0.9%)

Note: *k* (number of results) is 1188 unless indicated. See Appendix A for list of commonly used tests.

**Table 4:** Effect size by Level 2 moderators (sample and study characteristics).

Moderator	*k*	*r*	*SE*	CI.LB	CI.UB	*r* Controlling for Health Status
**Participant health status ^a^**						
Nonclinical	984	0.13 ***	0.004	0.121	0.137	
Cognitively impaired	27	0.04	0.031	−0.026	0.098	
Physical illness or condition	32	0.11 ***	0.026	0.056	0.158	
Neurodevelopmental disorder	16	0.06	0.038	−0.017	0.133	
Psychosis	86	0.04 *	0.016	0.008	0.073	
Affective disorder	25	0.04	0.029	−0.012	0.103	
Other mental/behavioral diagnoses	12	0.08 *	0.040	0.003	0.159	
**Study location ^b^**						
USA	462	0.13 ***	0.006	0.120	0.145	0.14 ***
Non-Anglophone Europe	325	0.12 ***	0.008	0.102	0.132	0.13 ***
Australia and New Zealand	68	0.12 ***	0.018	0.085	0.157	0.14 ***
Diverse countries	54	0.11 ***	0.017	0.081	0.147	0.14 ***
UK and Ireland	106	0.09 ***	0.014	0.064	0.118	0.10 ***
East and Southeast Asia	71	0.10 ***	0.017	0.068	0.133	0.12 ***
Canada	33	0.09 ***	0.025	0.040	0.138	0.09 ***
Central and South America, Mexico	28	0.09 ***	0.023	0.045	0.136	0.09 ***
Middle East	17	0.11 **	0.034	0.041	0.174	0.12 ***
**Participant mean age**						
8–12 years	77	0.09 ***	0.015	0.058	0.116	0.09 ***
13–17 years	67	0.18 ***	0.015	0.151	0.209	0.19 ***
18–27 years	516	0.14 ***	0.006	0.128	0.152	0.14 ***
>28 years or mix of 18–27 and >28	524	0.10 ***	0.006	0.084	0.106	0.11 ***
**Sample race ^c^**						
White	297	0.13 ***	0.008	0.118	0.148	0.14 ***
East and Southeast Asian	79	0.10 ***	0.016	0.069	0.131	0.11 ***
African American in USA	12	0.10 *	0.045	0.010	0.187	0.12 **
Mixture (no ethnic group compromised >60% of sample)	73	0.08 ***	0.015	0.054	0.114	0.09 ***
**First author gender**						
Male	483	0.11 ***	0.006	0.100	0.124	0.12 ***
Female	681	0.12 ***	0.006	0.114	0.135	0.14 ***
**Publication status**						
Published article	514	0.13 ***	0.006	0.123	0.145	0.14 ***
Result sent by author on our request (only from 2015 onward)	584	0.10 ***	0.006	0.085	0.109	0.11 ***
Master’s thesis or dissertation	42	0.14 ***	0.021	0.100	0.184	0.15 ***
Unpublished	20	0.15 ***	0.036	0.075	0.215	0.15 ***
Chapter or book	15	0.19 ***	0.034	0.119	0.252	0.18 ***
Effect size sent on request of Kirkland et al. (2013)	13	0.09 *	0.038	0.011	0.160	0.09 *

Note: *k* = number of effect sizes. *r* = Fisher-*z* normalization of the Pearson correlation coefficient. Positive values mean females scored higher; negative values mean males scored higher. CI.LB = 95% confidence interval, lower bound; CI.UB = 95% confidence interval, upper bound. For results of continuous Level 2 moderators (sample age and document date), see main text. ^a^ See Supplementary Materials File S3 for list of health conditions in each category. ^b^ Only countries/regions with more than 20 effect sizes were included in the moderator analysis. ^c^ Only ethnic groups that comprised 60% or more of participants in 10 or more effect sizes were included in the analysis. * *p* < .05 ** *p* < .01 *** *p* < .001.

**Table 5:** Effect size by Level 1 moderators (test characteristics).

Moderator	*k*	*r*	*SE*	CI.LB	CI.UB	*r* Controlling for Health Status
**Cue channel ^a^**						
Face	513	0.12 ***	0.006	0.105	0.127	0.16 ***
Masked voice	81	0.13 ***	0.014	0.105	0.158	0.13 ***
Body and hands	14	0.10 ***	0.030	0.041	0.159	0.14 ***
Full video with unmasked voice	65	0.12 ***	0.016	0.085	0.148	0.11 ***
Eyes	378	0.12 ***	0.006	0.102	0.127	0.12 ***
Full video with masked voice	71	0.13 ***	0.013	0.105	0.156	0.12 ***
Multichannel total	62	0.15 ***	0.015	0.120	0.180	0.14 ***
**Specific tests ^a^**						
Reading the Mind in the Eyes (RMET)	362	0.11 ***	0.006	0.099	0.124	0.12 ***
The Awareness of Social Inference Test (TASIT)	23	0.14 ***	0.023	0.093	0.182	0.15 ***
Diagnostic Analysis of Nonverbal Accuracy (DANVA), adult faces	45	0.15 ***	0.017	0.115	0.180	0.15 ***
ER40 (from Penn Computerized Neurocognitive Battery)	54	0.07 ***	0.016	0.041	0.101	0.09 ***
Pictures of Facial Affect (POFA), includes Brief Affect Recognition Task (BART)	110	0.14 ***	0.013	0.113	0.165	0.16 ***
Geneva Emotion Recognition Test (GERT)	34	0.16 ***	0.018	0.126	0.198	0.17 ***
Combination of two or more tests	34	0.15 ***	0.019	0.108	0.184	0.16 ***
Japanese and Caucasian Facial Expressions of Emotion (JACFEE)	24	0.09 **	0.028	0.034	0.144	0.10 ***
PONS	51	0.16 ***	0.018	0.130	0.200	0.17 ***
Other tests	451	0.12 ***	0.006	0.104	0.128	0.13 ***
**Stimulus presentation mode**						
Static	881	0.12 ***	0.004	0.107	0.124	0.13 ***
Dynamic	210	0.14 ***	0.009	0.118	0.152	0.14 ***
**Stimulus creation mode**						
Spontaneous	103	0.12 ***	0.005	0.112	0.133	0.14 ***
Posed	703	0.12 ***	0.013	0.091	0.141	0.12 ***
**Target gender**						
Male only	26	0.08 **	0.024	0.031	0.124	0.10 ***
Female only	90	0.13 ***	0.014	0.108	0.161	0.14 ***
Male and female	1001	0.12 ***	0.004	0.108	0.125	0.13 ***
**Target age**						
Child	26	0.14 ***	0.025	0.093	0.193	0.15 ***
Adult	1132	0.12 ***	0.004	0.109	0.125	0.13 ***
Child and adult	11	0.15 **	0.047	0.060	0.243	0.15 ***
**Target race/ethnicity**						
White	767	0.12 ***	0.004	0.113	0.130	0.13 ***
East Asian	33	0.12 ***	0.021	0.078	0.159	0.13 ***
Multiple ethnicities	174	0.10 ***	0.009	0.078	0.113	0.11 ***
**Authors’ analysis model**						
No covariates	1104	0.11 ***	0.004	0.105	0.121	0.12 ***
With covariates	67	0.21 ***	0.016	0.176	0.237	0.21 ***

Note: *k* = number of effect sizes. *r* = Fisher-*z* normalization of the Pearson correlation coefficient. Positive values mean females scored higher; negative values mean males scored higher. CI.LB = 95% confidence interval, lower bound; CI.UB = 95% confidence interval, upper bound. ^a^ Only cue channels and specific tests with 20 or more effect sizes were analyzed. For specific tests, all tests and stimulus sets that were used fewer than 20 times are subsumed in the “other tests” category. See Appendix A for description of instruments and citations. ** *p* < .01 *** *p* < .001.

## Note

1 Because comparison of cue channels is complicated when many different tests are involved, we performed analysis of results for one test (PONS) that has single- and multiple-cue modalities, using published data from the test’s development monograph (norm group of *N* = 480; Table 3.3 in Rosenthal et al. 1979). The 20 face-only items and the 40 voice-only items showed much smaller effects than did the full 220-item test—*r*s of 0.12, 0.10, and 0.24, respectively. The full test has face, body, full figure, and two methods of masking the verbal content of the voice items, all singly presented or in combination. However, body-only (20 items) showed a big gender effect (*d* = 0.24). So, for that large sample, the single channels did not necessarily show a smaller gender difference than combined channels. Also, Table 7.1 in Rosenthal et al. (1979), which summarizes 23 samples of participants, showed the same mixed picture, where the female advantage was not consistently bigger for combined-cue channels than for single-cue channels. We also looked at results for the PONS test in the current meta-analytic database. There are 51 PONS results, 16 involving single channels (face only, body only, or voice) and 35 involving multiple channels. The difference in the gender effect between single- and multiple-cue channels is significant, *F*(1, 49) = 5.02, *p* = .03 (*M r* for single channels = 0.10, *M r* for multiple channels = 0.18). However, for these 51 studies, the correlation between effect size and the number of items was 0.39, *p* < .004. When the number of items is controlled for, the difference disappears (*F* < 1, *p* = .52). For the PONS, therefore, the issue of whether single channels produce smaller effects than multiple channels is ambiguous.

## Supplementary Materials

The following supporting information can be downloaded at https://www.mdpi.com/article/10.3390/jintelligence13030038/s1, File S1. Tests Searched by Name; File S2. Citations for Sources; File S3. Codebook; Files S4 and S5. Coded Studies (in SPSS and Excel, respectively).

## Appendix A

**Table A1:** Description of tests used in 20 or more studies.

Test	Stimulus	Cue	Cue
	Creation Mode	Channels	Content
Pictures of Facial Affect (POFA; (Ekman and Friesen 1976))	Posed	Face	Basic emotions (usually 6)
Diagnostic Analysis of Nonverbal Accuracy-Adult Faces (DANVA; (Nowicki and Duke 1994))	Posed	Face	4 basic emotions
Japanese and Caucasian Facial Expressions of Emotion (JACFEE; (Biehl et al. 1997))	Posed	Face	7 basic emotions
Penn Emotion Identification Test (ER40; (Gur et al. 2012))	Posed	Face	4 basic emotions
Reading the Mind in the Eyes Test (RMET; (Baron-Cohen et al. 2001))	Unknown	Eye region	36 affective states
Profile of Nonverbal Sensitivity (PONS; (Rosenthal et al. 1979))	Posed	Video with face, body, and masked voice (alone and in all combinations)	20 affective scenarios
The Awareness of Social Inference Test-Emotion Evaluation Test (TASIT-EET; (McDonald et al. 2006))	Posed	Full video with masked voice	6 basic emotions
Geneva Emotion Recognition Test (GERT; (Schlegel et al. 2014))	Posed	Full video with masked voice	14 emotions

Note: Specific studies could vary details of test design and application. Stimulus sets are sometimes called by different names (e.g., POFA photographs called FEEST or Mini-SEA).
