# Supplementary material for: Gender and Accuracy in Decoding Affect Cues: A Meta-Analysis
Source: J Intell. 2025 Mar 18;13(3):38. doi: 10.3390/jintelligence13030038 (PMC11943105; doi:10.3390/jintelligence13030038)
Supplement: Supplementary file 1 [file jintelligence-13-00038-s001.zip › S1 Tests searched by name for gender and affect meta-analysis.pdf]

S1: Tests searched by name for "Gender and Accuracy in Decoding Affect Cues: A Meta-Analysis"

(All versions of these tests were included)

Profile of Nonverbal Sensitivity (PONS; Rosenthal et al., 1979)
Diagnostic Assessment of Nonverbal Accuracy (DANVA; Nowicki & Duke, 1994; Pitterman & Nowicki, 2004)
METT/SETT (Ekman, 2003)
Japanese and Caucasian Facial Expressions of Emotion (JACFEE; Biehl & Matsumoto, 1997)
Japanese and Caucasian Brief Affect Recognition Test (JACBART; Matsumoto et al., 2000)
Geneva Emotion Recognition Test (GERT; Schlegel et al., 2014)
Multimodal Emotion Recognition Test (MERT; Bänziger et al., 2014)
Reading the Mind in the Eyes Test (RMET; Baron-Cohen et al., 2001)
Reading the Mind in the Voice test (Golan et al., 2007)
Reading the Mind in Films test (Golan et al., 2006)
Radboud Faces Database (Langner et al., 2010)
Amsterdam Dynamic Facial Expression Set (ADFES; van der Schalk et al., 2011)
Karolinska Directed Emotional Faces (Calvo & Lundqvist, 2008)
NimStim (Tottenham et al., 2009)
Bell-Lysaker Emotion Recognition Task (BLERT; Bryson et al., 1997)
Montreal Set of Facial Displays of Emotion (MSFDE; Beaupré & Hess, 2005)
Chinese Facial Affective Picture System (Gong et al., 2011)
Korea University Facial Expression Collection (KUFEC; Kim et al., 2017)
The Awareness of Social Inference Test (TASIT-EET Part 1, Emotion Recognition; McDonald et al. 2003)
Pictures of Facial Affect (POFA; Ekman & Friesen, 1976)
Facial Expressions of Emotions—Stimuli and Test (FEEST; Young et al., 2002, based on POFA)
Mini-SEA (Part 1, Emotion Recognition, Bertoux et al., 2012, based on POFA)
Communication of Affect Receiving Ability Test (CARAT; Buck, 1976)
Penn Emotion Recognition Task (ER-40; Kohler et al., 2003)

Citations for tests:

Bänziger, T., Grandjean, D., & Scherer, K. R. (2009). Emotion recognition from expressions in face, voice, and body: The Multimodal Emotion Recognition Test (MERT). *Emotion, 9*(5), 691–704.

Baron-Cohen, S., Wheelwright, S., Hill, J., Raste, Y., & Plumb, I. (2001). The "Reading the mind in the eyes" Test revised version: A study with normal adults, and adults with Asperger syndrome or high-functioning autism. *Journal of Child Psychology and Psychiatry, 42*(2), 241–251.

Beaupré, M. G., & Hess, U. (2005). Cross-cultural emotion recognition among Canadian ethnic groups. *Journal of Cross-Cultural Psychology, 36*(3), 355–370.

Bertoux, M., Delavest, M., de Souza, L. C., Funkiewiez, A., Lépine, J.-P., Fossati, P., Dubois, B., & Sarazin, M. (2012). Social Cognition and Emotional Assessment differentiates

frontotemporal dementia from depression. *Journal of Neurology, Neurosurgery & Psychiatry, 83*(4), 411–416.

Biehl, M., Matsumoto, D., Ekman, P., Hearn, V., Heider, K., Kudoh, T., & Ton, V. (1997). Matsumoto and Ekman's Japanese and Caucasian Facial Expressions of Emotion (JACFEE): Reliability data and cross-national differences. *Journal of Nonverbal Behavior, 21*(1), 3–21.

Bryson, G., Bell, M., & Lysaker, P. (1997). Affect recognition in schizophrenia: A function of global impairment or a specific cognitive deficit. *Psychiatry Research, 71*(2), 105–113.

Buck, R. (1976). A test of nonverbal receiving ability: Preliminary studies. *Human Communication Research, 2*(2), 162–171.

Calvo, M. G., & Lundqvist, D. (2008). Facial expressions of emotion (KDEF): Identification under different display-duration conditions. *Behavior Research Methods, 40*(1), 109-115.

Ekman, P. (2003). SETT, Subtle Expression Training Tool; METT, Micro Expression Training Tool. Mozgo Media.

Ekman, P., & Friesen, W. (1976). Pictures of facial affect. Consulting Psychologists Press.

Golan, O., Baron-Cohen, S., Hill, J. J., & Golan, Y. (2006). The "Reading the Mind in Films" task: Complex emotion in adults with and without autism spectrum conditions. *Social Neuroscience, 1*(2), 111-123.

Golan, O., Baron-Cohen, S., Hill, J. J., & Rutherford, M. D. (2007). The 'Reading the Mind in the Voice' Test-Revised: A study of complex emotion recognition in adults with and without autism spectrum conditions. *Journal of Autism and Developmental Disorders, 37*(6), 1096-1106.

Gong, X., Huang, Y.-X., Wang, Y., & Luo, Y.-J. (2011). Revision of the Chinese Facial Affective Picture System. *Chinese Mental Health Journal, 25*(1), 40–46.

Kim, S.-M., Kwon, Y.-J., Jung, S.-Y., Kim, M.-J., Cho, Y. S., Kim, H. T., Nam, K.-C., Kim, H., Choi, K.-H., & Choi, J.-S. (2017). Development of the Korean facial emotion stimuli: Korea University Facial Expression Collection 2nd Edition. *Frontiers in Psychology, 8,* Article 769.

Kohler, C. G., Turner, T. H., Bilker, W. B., Brensinger, C. M., Siegel, S. J., Kanes, S. J., Gur, R. E., & Gur, R. C. (2003). Facial emotion recognition in schizophrenia: Intensity effects and error pattern. *American Journal of Psychiatry, 160*(10), 1768–1774.

Langner, O., Dotsch, R., Bijlstra, G., Wigboldus, D. H. J., Hawk, S. T., & van Knippenberg, A. (2010). Presentation and validation of the Radboud Faces Database. *Cognition and Emotion, 24*(8), 1377-1388.

Matsumoto, D., LeRoux, J., Wilson-Cohn, C., Raroque, J., Kooken, K., Ekman, P., Yrizarry, N., Loewinger, S., Uchida, H., Yee, A., Amo, L., & Goh, A. (2000). A new test to measure emotion recognition ability: Matsumoto and Ekman's Japanese and Caucasian Brief Affect Recognition Test (JACBART). *Journal of Nonverbal Behavior, 24*(3), 179-209.

Nowicki, S., & Duke, M. P. (1994). Individual differences in the nonverbal communication of affect: The Diagnostic Analysis of Nonverbal Accuracy Scale. *Journal of Nonverbal Behavior, 18*(1), 9–35.

Pitterman, H., & Nowicki, S., Jr. (2004). A Test of the Ability to Identify Emotion in Human Standing and Sitting Postures: The Diagnostic Analysis of Nonverbal Accuracy-2 Posture Test (DANVA2-POS). *Genetic, Social, and General Psychology Monographs, 130*(2), 146–162.

Rosenthal, R., Hall, J. A., DiMatteo, M. R. Rogers, P. L., & Archer, D. (1979). *Sensitivity to nonverbal cues: The PONS test.* Johns Hopkins University Press.
Schlegel, K., Grandjean, D., & Scherer, K. R. (2014). Introducing the Geneva Emotion Recognition Test: An example of Rasch-based test development. *Psychological Assessment, 26*(2), 666–672.
Tottenham, N., Tanaka, J. W., Leon, A. C., McCarry, T., Nurse, M., Hare, T. A., Marcus, D. J., Westerlund, A., Casey, B. J., & Nelson, C. (2009). The NimStim set of facial expressions: Judgments from untrained research participants. *Psychiatry Research, 168*(3), 242–249.
van der Schalk, J., Hawk, S. T., Fischer, A. H., & Doosje, B. (2011). Moving faces, looking places: Validation of the Amsterdam Dynamic Facial Expression Set (ADFES). *Emotion, 11*(4), 907–920.
Young, A., Perrett, D., Calder, A., Sprengelmeyer, R., & Ekman, P. (2002). Facial expressions of emotions: Stimuli and tests (FEEST). Thames Valley Test.
