# Supplementary material for: Gender and Accuracy in Decoding Affect Cues: A Meta-Analysis
Source: J Intell. 2025 Mar 18;13(3):38. doi: 10.3390/jintelligence13030038 (PMC11943105; doi:10.3390/jintelligence13030038)
Supplement: Supplementary file 1 [file jintelligence-13-00038-s001.zip › S2 Citations of sources in the gender and affect meta-analysis.pdf]

S2 Citations of sources providing quantitative data for "Gender and Accuracy in Decoding Affect Cues: A Meta-Analysis"

Numbers preceding the citation are internally used ID numbers.

8001 aan het Rot, M., & Hogenelst, K (2014). The influence of affective empathy and autism spectrum traits on empathic accuracy. *PLoS ONE 9*(6): e98436.

652 Abbruzzese, L., Magnani, N., Robertson, I. H., & Mancuso, M. (2019). Age and gender differences in emotion recognition. *Frontiers in Psychology, 10,* Article 2371.

7115 Abrams, R. F. (1999). Social anxiety, social evaluative threat, and the interpretation of nonverbal displays of emotion. *Dissertation Abstracts International: Section B: The Sciences and Engineering, 59*(11-B), 6058.

6086 Adams, A. G., Henry, J. D., Molenberghs, P., Robinson, G. A., Nott, Z., & von Hippel, W. (2020). The relationship between social cognitive difficulties in the acute stages of stroke and later functional outcomes. *Social Neuroscience, 15*(2), 158–169.

6252 Akgül, Ö., Küçükçoban, O., Binbay, T., Bora, E., Alptekin, K., & Akdede, B. B. (2017). Do clinical features relate to theory of mind, empathy and 2D:4D in schizophrenia? *Psychiatry and Clinical Psychopharmacology, 27*(4), 380–385.

5522 Alaerts, K., Nackaerts, E., Meyns, P., Swinnen, S. P., & Wenderoth, N. (2011). Action and emotion recognition from point light displays: An investigation of gender differences. *PLoS ONE, 6*(6), Article e20989.

7595 Alfimova, M. V., Abramova, L. I., Barhatova, A. I., Yumatova, P. E., Lyachenko, G. L., Golimbet, V. E. (2009). Facial affect recognition deficit as a marker of genetic vulnerability to schizophrenia. *Spanish Journal of Psychology 12*, 46–55.

6670 Ali, F., & Chamorro-Premuzic, T. (2010). Investigating Theory of Mind deficits in nonclinical psychopathy and Machiavellianism. *Personality and Individual Differences, 49*(3), 169–174.

755 Alkozei, A., Smith, R., Demers, L. A., Weber, M., Berryhill, S. M., & Killgore, W. D. S. (2019). Increases in emotional intelligence after an online training program are associated with better decision-making on the Iowa gambling task. *Psychological Reports, 122*(3), 853–879.

1818 Allott, K. A., Rice, S., Bartholomeusz, C. F., Klier, C., Schlögelhofer, M., Schäfer, M. R., & Amminger, G. P. (2015). Emotion recognition in unaffected first-degree relatives of individuals with first-episode schizophrenia. *Schizophrenia Research, 161*(2-3), 322–328.

99 Altmann, T., & Roth, M. (2021). The risk of empathy: Longitudinal associations between empathy and burnout. *Psychology & Health, 36*(12), 1441–1460.

549 Alvi, T., Kouros, C. D., Lee, J., Fulford, D., & Tabak, B. A. (2020). Social anxiety is negatively associated with theory of mind and empathic accuracy. *Journal of Abnormal Psychology, 129*(1), 108–113.

223 Amorim, M., Anikin, A., Mendes, A. J., Lima, C. F., Kotz, S. A., & Pinheiro, A. P. (2021). Changes in vocal emotion recognition across the life span. *Emotion, 21*(2), 315–325.

5533 Anderson, I. M., Shippen, C., Juhasz, G., Chase, D., Thomas, E., Downey, D., Toth, Z. G., Lloyd-Williams, K., Elliott, R., & Deakin, J. F. W. (2011). State-dependent alteration in face emotion recognition in depression. *The British Journal of Psychiatry, 198*(4), 302–308.

1539 Andric, S., Maric, N. P., Knezevic, G., Mihaljevic, M., Mirjanic, T., Velthorst, E., & van Os, J. (2016). Neuroticism and facial emotion recognition in healthy adults. *Early Intervention in Psychiatry, 10*(2), 160–164.

1406 Andric, S., Maric, N. P., Mihaljevic, M., Mirjanic, T., & van Os, J. (2016). Familial covariation of facial emotion recognition and IQ in schizophrenia. *Psychiatry Research, 246,* 52–57.

6419 Aradhye, C., Vonk, J., & Arida, D. (2015). Adults' responsiveness to children's facial expressions. *Journal of Experimental Child Psychology, 135,* 56–71.

5719 Asla, N., de Paúl, J., & Pérez-Albéniz, A. (2011). Emotion recognition in fathers and mothers at high-risk for child physical abuse. *Child Abuse & Neglect, 35*(9), 712–721.

5575 Atkins, D., Uskul, A. K., & Cooper, N. R. (2016). Culture shapes empathic responses to physical and social pain. *Emotion, 16*(5), 587–601.

7597 Axelrod, L. (1982). Social perception in learning disabled adolescents. *Journal of Learning Disabilities, 15*, 610-613.

771 Aydın, O., Balıkçı, K., Çökmüş, F. P., & Ünal Aydın, P. (2019). The evaluation of metacognitive beliefs and emotion recognition in panic disorder and generalized anxiety disorder: Effects on symptoms and comparison with healthy control. *Nordic Journal of Psychiatry, 73*(4-5), 293–301.

7379 Babchuk, W. A., Hames, R. B., & Thompson, R. A. (1985). Sex differences in the recognition of infant facial expressions of emotion: The primary caretaker hypothesis. *Ethology & Sociobiology, 6*(2), 89–101.
6427 Bączkowski, B. M., & Cierpiałkowska, L. (2015). Mentalization within close relationships: The role of specific attachment style. *Polish Psychological Bulletin, 46*(2), 285–299.
7503 Bailey, P. E., Henry, J. D., & Von Hippel, W. (2008). Empathy and social functioning in late adulthood. *Aging & Mental Health, 12*(4), 499–503.
6224 Baksh, R. A., Abrahams, S., Auyeung, B., & MacPherson, S. E. (2018). The Edinburgh Social Cognition Test (ESCoT): Examining the effects of age on a new measure of theory of mind and social norm understanding. *PLoS ONE, 13*(4), Article e0195818.
204 Balter, L. J. T., Raymond, J. E., Aldred, S., Higgs, S., & Bosch, J. A. (2021). Age, BMI, and inflammation: Associations with emotion recognition. *Physiology & Behavior, 232,* Article 113324.
7330 Banks, S. H. (2008). Curiosity and narcissism as predictors of empathic ability. *Dissertation Abstracts International: Section B: The Sciences and Engineering, 68*(10-B), 6950.
5502 Bänziger, T., Scherer, K. R., Hall, J. A., & Rosenthal, R. (2011). Introducing the MiniPONS: A short multichannel version of the Profile of Nonverbal Sensitivity (PONS). *Journal of Nonverbal Behavior, 35*(3), 189–204.
6428 Baribeau, D. A., Doyle-Thomas, K. A. R., Dupuis, A., Iaboni, A., Crosbie, J., McGinn, H., Arnold, P. D., Brian, J., Kushki, A., Nicolson, R., Schachar, R. J., Soreni, N., Szatmari, P., & Anagnostou, E. (2015). Examining and comparing social perception abilities across childhood-onset neurodevelopmental disorders. *Journal of the American Academy of Child & Adolescent Psychiatry, 54*(6), 479–486.
6411 Baron-Cohen, S., Bowen, D. C., Holt, R. J., Allison, C., Auyeung, B., Lombardo, M. V., Smith, P., & Lai, M.-C. (2015). The “Reading the Mind in the Eyes” test: Complete absence of typical sex difference in ~400 men and women with autism. *PLoS ONE, 10*(8), Article e0136521.
8013 Baron-Cohen, S., & Hammer, J. (1997). Is autism an extreme form of the “male brain”? *Advances in Infancy Research, 11*, 193-217.
8009 Baron-Cohen, S., Jolliffe, T., Mortimore, C., & Robertson, M. (1997). Another advanced test of theory of mind: Evidence from very high functioning adults with autism or Asperger Syndrome. *Child Psychology & Psychiatry & Allied Disciplines, 38*(7), 813–822.
6725 Baron-Cohen, S., Wheelwright, S., Hill, J., Raste, Y., & Plumb, I. (2001). The "Reading the Mind in the Eyes" Test revised version: A study with normal adults, and adults with Asperger syndrome or high-functioning autism. *Journal of Child Psychology and Psychiatry, 42*(2), 241–251.
275 Basile, A., Toplak, M. E., & Andrade, B. F. (2021). Using metacognitive methods to examine emotion recognition in children with ADHD. *Journal of Attention Disorders, 25*(2), 245–257.
409 Basner, M., Hermosillo, E., Nasrini, J., Saxena, S., Dinges, D. F., Moore, T. M., & Gur, R. C. (2020). Cognition test battery: Adjusting for practice and stimulus set effects for varying administration intervals in high performing individuals. *Journal of Clinical and Experimental Neuropsychology, 42*(5), 516–529.
6147 Belardinelli, M. O., Huenefeldt, T., Maffi, S., Squitieri, F., & Migliore, S. (2019). Effects of stimulus-related variables on mental states recognition in Huntington’s disease. *International Journal of Neuroscience, 129*(6), 563–572.
7523 Beldoch, M. Sensitivity to expression of emotional meaning in three modes of communication. In J. R. Davitz (Ed.), *The communication of emotional meaning*. McGraw-Hill, 1964.
6087 Bellosta-Batalla, M., Blanco-Gandía, M. C., Rodríguez-Arias, M., Cebolla, A., Pérez-Blasco, J., & Moya-Albiol, L. (2020). Increased salivary oxytocin and empathy in students of clinical and health psychology after a mindfulness and compassion-based intervention. *Mindfulness, 11*(4), 1006–1017.
407 Benau, E. M., Wiatrowski, R., & Timko, C. A. (2020). Difficulties in emotion regulation, alexithymia, and social phobia are associated with disordered eating in male and female undergraduate athletes. *Frontiers in Psychology, 11,* Article 1646.
7001 Berenbaum, H., & Prince, J. D. (1994). Alexithymia and the interpretation of emotion-relevant information. *Cognition and Emotion, 8*(3), 231–244.
6228 Berenson, K. R., Dochat, C., Martin, C. G., Yang, X., Rafaeli, E., & Downey, G. (2018). Identification of mental states and interpersonal functioning in borderline personality disorder. *Personality Disorders: Theory, Research, and Treatment, 9*(2), 172–181.
5954 Berg, S. K., Bedwell, J. S., Dvorak, R. D., & Tone, E. B. (2021). Higher social anxiety severity predicts better cognitive empathy performance in women but not men. *Psychological Reports, 124*(6), 2549–2566.
9010 Berney, A., Bourquin, C., Carrard, V., Berney, S., Schlegel, K., Gaume, J., Gholam, M., Bart, P.-A., Preisig, M., Wac, K., & Mast, M. S. (2022). Unpublished data, Lausanne University Hospital.

1790 Bertoux, M., de Souza, L. C., Sarazin, M., Funkiewiez, A., Dubois, B., & Hornberger, M. (2015). How preserved is emotion recognition in Alzheimer disease compared with behavioral variant frontotemporal dementia? *Alzheimer Disease and Associated Disorders, 29*(2), 154–157.
321 Bertrams, A., & Schlegel, K. (2020). Speeded reasoning moderates the inverse relationship between autistic traits and emotion recognition. *Autism, 24*(8), 2304–2309.
6917 Besel, L. D. S., & Yuille, J. C. (2010). Individual differences in empathy: The role of facial expression recognition. *Personality and Individual Differences, 49*(2), 107–112.
1517 Bhatara, A., Laukka, P., Boll-Avetisyan, N., Granjon, L., Elfenbein, H. A., & Bänziger, T. (2016). Second language ability and emotional prosody perception. *PLoS ONE, 11*(6), Article e0156855.
5843 Biehl, M., Matsumoto, D., Ekman, P., Hearn, V., Heider, K., Kudoh, T., & Ton, V. (1997). Matsumoto and Ekman's Japanese and Caucasian Facial Expressions of Emotion (JACFEE): Reliability data and cross-national differences. *Journal of Nonverbal Behavior, 21*(1), 3–21.
7226 Bjornsdottir, R. T., Alaei, R., & Rule, N. O. (2017). The perceptive proletarian: Subjective social class predicts interpersonal accuracy. *Journal of Nonverbal Behavior, 41*(2), 185–201.
6352 Bjornsdottir, R. T., & Rule, N. O. (2016). On the relationship between acculturation and intercultural understanding: Insight from the Reading the Mind in the Eyes test. *International Journal of Intercultural Relations, 52,* 39–48.
6140 Black, J. E. (2019). An IRT analysis of the Reading the Mind in the Eyes Test. *Journal of Personality Assessment, 101*(4), 425–433.
6394 Black, J., & Barnes, J. L. (2015). Fiction and social cognition: The effect of viewing award-winning television dramas on theory of mind. *Psychology of Aesthetics, Creativity, and the Arts, 9*(4), 423–429.
6072 Black, J. E., & Barnes, J. L. (2020). Recognition as a measure of television exposure: Multiple measures and their relationship to theory of mind. *Psychology of Popular Media, 9*(3), 287–296.
5966 Black, J. E., & Barnes, J. L. (2021). Does writing promote social cognition? The role of fictionality and social content. *Psychology of Aesthetics, Creativity, and the Arts.* Advance online publication.
5544 Blanch-Hartigan, D. (2011). Measuring providers' verbal and nonverbal emotion recognition ability: Reliability and validity of the Patient Emotion Cue Test (PECT). *Patient Education and Counseling, 82*(3), 370–376.
6679 Blanchard, A., & Lyons, M. (2010). An investigation into the relationship between digit length ratio (2D: 4D) and psychopathy. *The British Journal of Forensic Practice, 12*(2), 23–31.
7148 Blanck, P. D., Rosenthal, R., Snodgrass, S. E., DePaulo, B. M., & Zuckerman, M. (1981). Sex differences in eavesdropping on nonverbal cues: Developmental changes. *Journal of Personality and Social Psychology, 41*(2), 391–396.
66 Blickle, G., Kranefeld, I., Wihler, A., Kückelhaus, B. P., & Menges, J. I. (2022). It works without words: A nonlinguistic ability test of perceiving emotions with job-related consequences. *European Journal of Psychological Assessment, 38*(3), 210–223.
2543 Blickle, G., Momm, T. S., Kramer, J., Mierke, J., Liu, Y., & Ferris, G. R. (2009). Construct and criterion-related validation of a measure of emotional reasoning skills: A two-study investigation. *International Journal of Selection and Assessment, 17*(1), 101–118.
5933 Blose, B. A., & Schenkel, L. S. (2022). Theory of mind and alexithymia in deaf and hard-of-hearing young adults. *Journal of Deaf Studies and Deaf Education, 27*(2), 179–192.
5565 Bommer, W. H., Pesta, B. J., & Storrud-Barnes, S. F. (2011). Nonverbal emotion recognition and performance: Differences matter differently. *Journal of Managerial Psychology, 26*(1), 28–41.
655 Bone, J. K., Lewis, G., Button, K. S., Duffy, L., Harmer, C. J., Munafò, M. R., Penton-Voak, I. S., Wiles, N. J., & Lewis, G. (2019). Variation in recognition of happy and sad facial expressions and self-reported depressive symptom severity: A prospective cohort study. *Journal of Affective Disorders, 257,* 461–469.
7383 Bonebright, T. L., Thompson, J. L., & Leger, D. W. (1996). Gender stereotypes in the expression and perception of vocal affect. *Sex Roles: A Journal of Research, 34*(5-6), 429–445.
6412 Bormann, D., & Greitemeyer, T. (2015). Immersed in virtual worlds and minds: Effects of in-game storytelling on immersion, need satisfaction, and affective theory of mind. *Social Psychological and Personality Science, 6*(6), 646–652.
6389 Bosgelmez, S., Yildiz, M., Yazici, E., Inan, E., Turgut, C., Karabulut, U., Kircali, A., Tas, H. I., Yakisir, S. S., Cakir, U., & Sungur, M. Z. (2015). Reliability and validity of the Turkish version of cognitive assessment interview (CAI-TR). *Klinik Psikofarmakoloji Bülteni / Bulletin of Clinical Psychopharmacology, 25*(4), 365–380.

857 Bosnjak Kuharic, D., Makaric, P., Kekin, I., Lukacevic Lovrencic, I., Savic, A., Ostojic, D., Silic, A., Brecic, P., Bajic, Z., & Rojnic Kuzman, M. (2019). Differences in facial emotional recognition between patients with the first-episode psychosis, multi-episode schizophrenia, and healthy controls. *Journal of the International Neuropsychological Society, 25*(2), 165–173.

1083 Bossi, F., Gallucci, M., & Ricciardelli, P. (2018). How social exclusion modulates social information processing: A behavioural dissociation between facial expressions and gaze direction. *PLoS ONE, 13*(4), Article e0195100.

878 Brand, S., Schilling, R., Ludyga, S., Colledge, F., Sadeghi Bahmani, D., Holsboer-Trachsler, E., Pühse, U., & Gerber, M. (2019). Further evidence of the zero-association between symptoms of insomnia and facial emotion recognition—results from a sample of adults in their late 30s. *Frontiers in Psychiatry, 9,* Article 754.

6619 Bremser, J. A., & Gallup, G. G., Jr. (2012). From one extreme to the other: Negative evaluation anxiety and disordered eating as candidates for the extreme female brain. *Evolutionary Psychology, 10*(3), 457–486.

1105 Brislin, S. J., Yancey, J. R., Perkins, E. R., Palumbo, I. M., Drislane, L. E., Salekin, R. T., Fanti, K. A., Kimonis, E. R., Frick, P. J., Blair, R. J. R., & Patrick, C. J. (2018). Callousness and affective face processing in adults: Behavioral and brain-potential indicators. *Personality Disorders: Theory, Research, and Treatment, 9*(2), 122–132.

5934 Bronstein, M. V., Everaert, J., Kummerfeld, E., Haynos, A. F., & Vinogradov, S. (2022). Biased and inflexible interpretations of ambiguous social situations: Associations with eating disorder symptoms and socioemotional functioning. *International Journal of Eating Disorders, 55*(4), 518–529.

6482 Brosnan, M., Hollinworth, M., Antoniadou, K., & Lewton, M. (2014). Is Empathizing intuitive and Systemizing deliberative? *Personality and Individual Differences, 66,* 39–43.

7216 Brown, C. L., Lwi, S. J., Goodkind, M. S., Rankin, K. P., Merrilees, J., Miller, B. L., & Levenson, R. W. (2018). Empathic accuracy deficits in patients with neurodegenerative disease: Association with caregiver depression. *The American Journal of Geriatric Psychiatry, 26*(4), 484–493.

6149 Brown, M. I., Ratajska, A., Hughes, S. L., Fishman, J. B., Huerta, E., & Chabris, C. F. (2019). The social shapes test: A new measure of social intelligence, mentalizing, and theory of mind. *Personality and Individual Differences, 143,* 107–117.

7385 Brunori, P., Ladavas, E., & Ricci Bitti, P. E. (1979). Differential aspects in the recognition of facial expression of emotions. *Italian Journal of Psychology, 6*(3), 265–272.

7588 Buchman, J. S. (1973). Nonverbal communication of emotions among New York City cultural groups. Doctoral dissertation, Columbia University. *Dissertation Abstracts International, 34*, 406–407.

6132 Buchweitz, A., de Azeredo, L. A., Sanvicente-Vieira, B., Metsavaht Cará, V., Bianchini Esper, N., Soder, R. B., de Costa, J. C., Portuguez, M. W., Franco, A. R., & Grassi-Oliveira, R. (2019). Violence and Latin-American preadolescents: A study of social brain function and cortisol levels. *Developmental Science, 22*(5), Article e12799.

7443 Buck, R. (1976). A test of nonverbal receiving ability: Preliminary studies. *Human Communication Research, 2*(2), 162–171.

1577 Burkhouse, K. L., Woody, M. L., Owens, M., McGeary, J. E., Knopik, V. S., & Gibb, B. E. (2016). Sensitivity in detecting facial displays of emotion: Impact of maternal depression and oxytocin receptor genotype. *Cognition and Emotion, 30*(2), 275–287.

1264 Buunk, A. M., Spikman, J. M., Veenstra, W. S., van Laar, P. J., Metzemaekers, J. D. M., van Dijk, J. M. C., Meiners, L. C., & Groen, R. J. M. (2017). Social cognition impairments after aneurysmal subarachnoid haemorrhage: Associations with deficits in interpersonal behaviour, apathy, and impaired self-awareness. *Neuropsychologia, 103,* 131–139.

7562 Byron, K. (2007). Male and female managers' ability to 'read' emotions: Relationships with supervisor's performance ratings and subordinates satisfaction ratings. *Journal of Occupational and Organizational Psychology, 80*(4), 713–733.

2625 Byron, K., Terranova, S., & Nowicki, S., Jr. (2007). Nonverbal emotion recognition and salespersons: Linking ability to perceived and actual success. *Journal of Applied Social Psychology, 37*(11), 2600–2619.

6141 Caldú, X., Ottino-González, J., Sánchez-Garre, C., Hernan, I., Tor, E., Sender-Palacios, M.-J., Dreher, J.-C., Garolera, M., & Jurado, M. Á. (2019). Effect of the catechol- *o* -methyltransferase val ^{158}met polymorphism on theory of mind in obesity. *European Eating Disorders Review, 27*(4), 401–409.

7572 Calvi, J. L. (2011). The relationship between self-report and behavioral measures of empathy. Master’s thesis, University of North Texas, Denton, TX.

1409 Calvo, M. G., Avero, P., Fernández-Martín, A., & Recio, G. (2016). Recognition thresholds for static and dynamic emotional faces. *Emotion, 16*(8), 1186–1200.

6012 Camodeca, A., Walcott, K., Hosack, A., & Todd, K. Q. (2021). Preliminary evidence for the Emotion Word Fluency Test as a unique semantic fluency measure. *Psychological Assessment, 33*(2), 195–200.
1909 Campbell, A., Ruffman, T., Murray, J. E., & Glue, P. (2014). Oxytocin improves emotion recognition for older males. *Neurobiology of Aging, 35*(10), 2246–2248.
5916 Canal, P., Bischetti, L., Bertini, C., Ricci, I., Lecce, S., & Bambini, V. (2022). N400 differences between physical and mental metaphors: The role of Theories of Mind. *Brain and Cognition, 161,* 1–13.
7390 Carlson, R., & Levy, N. (1973). Studies of Jungian typology: I. Memory, social perception, and social action. *Journal of Personality, 41*(4), 559–576.
8003 Carrard, V., Bourquin, C., Berney, S., Schlegel, K., Gaume, J., Bart, P-A., Preisig, M., Schmid Mast, M., & Berney, A. (2022). The relationship between medical students' empathy, mental health, and burnout: A cross-sectional study. *Medical Teacher, 44*(12), 1392-1399.
7579 Carroll, J. M., & Yung, C. K. (2006). Sex and discipline differences in empathising, systemising and autistic symptomatology: Evidence from a student population. *Journal of Autism and Developmental Disorders, 36*(7), 949–957.
9007 Carter, J. D., & Hall, J. A. (2006). Unpublished data, Northeastern University.
7120 Carton, E. E. (1996). Children's sociometric status: Sex differences in the correlates of peer acceptance and rejection. *Dissertation Abstracts International: Section B: The Sciences and Engineering, 57*(4-B), 2903.
679 Cary, J., LaFrance, J., Costello, S., & Bovier, E. R. (2019). Olfactory identification, odor hedonics, and atypical behaviors. *Psi Chi Journal of Psychological Research, 24*(3), 184–193.
36 Cassidy, B. S., Wiley, R. W., Sim, M., & Hugenberg, K. (2022). Decoding complex emotions and humanization show related face processing effects. *Emotion, 22*(2), 362–373.
6083 Castano, E., Martingano, A. J., & Perconti, P. (2020). The effect of exposure to fiction on attributional complexity, egocentric bias and accuracy in social perception. *PLoS ONE, 15*(5), Article e0233378.
1696 Castro, V. L., & Boone, R. T. (2015). Sensitivity to spatiotemporal percepts predicts the perception of emotion. *Journal of Nonverbal Behavior, 39*(3), 215–240.
681 Castro, V. L., & Isaacowitz, D. M. (2019). The same with age: Evidence for age-related similarities in interpersonal accuracy. *Journal of Experimental Psychology: General, 148*(9), 1517–1537.
5971 Catalino, L. I., & Boulton, A. J. (2021). The psychometric properties of the Prioritizing Positivity Scale. *Journal of Personality Assessment, 103*(5), 705–715.
277 Cavieres, A., Maldonado, R., Bland, A., & Elliott, R. (2021). Relationship between gender and performance on emotion perception tasks in a Latino population. *International Journal of Psychological Research, 14*(1), 106–114.
7505 Chakrabarti, B., Dudbridge, E., Kent, L., Wheelwright, S., Hill-Cawthorne, G., Allison, C., Banerjee-Basu, S., & Baron-Cohen, S. (2009). Genes related to sex steroids, neural growth, and social-emotional behavior are associated with autistic traits, empathy, and Asperger syndrome. *Autism Research, 2*(3), 157–177.
6045 Chander, R. J., Grainger, S. A., Crawford, J. D., Mather, K. A., Numbers, K., Cleary, R., Kochan, N. A., Brodaty, H., Henry, J. D., & Sachdev, P. S. (2020). Development of a short-form version of the Reading the Mind in the Eyes Test for assessing theory of mind in older adults. *International Journal of Geriatric Psychiatry, 35*(11), 1322–1330.
552 Charernboon, T. (2020). Different subdomains of negative symptoms in clinically stable patients with schizophrenia: Determining the nature of their relationships with emotion recognition, theory of mind and neurocognition. *Cogent Psychology, 7*(1), Article 1849892.
186 Chen, W., McDonald, S., Wearne, T., & Grisham, J. (2021). Investigating associations between hoarding symptoms and affective and cognitive empathy. *British Journal of Clinical Psychology, 60*(2), 177–193.
6203 Cherry, M. G., Taylor, P. J., Brown, S. L., & Sellwood, W. (2018). Attachment, mentalisation and expressed emotion in carers of people with long-term mental health difficulties. *BMC Psychiatry, 18,* Article 257.
6234 Chiu, C.-D., & Yeh, Y.-Y. (2018). In your shoes or mine? Shifting from other to self perspective is vital for emotional empathy. *Emotion, 18*(1), 39–45.
1717 Chiu, I., Gfrörer, R. I., Piguet, O., Berres, M., Monsch, A. U., & Sollberger, M. (2015). "Now I see it, now I don't": Determining threshold levels of facial emotion recognition for use in patient populations. *Journal of the International Neuropsychological Society, 21*(7), 568–572.
1712 Chung, J. M., & Robins, R. W. (2015). Exploring cultural differences in the recognition of the self-conscious emotions. *PLoS ONE, 10*(8), Article e0136411.
5706 Ciucci, E., Baroncelli, A., & Nowicki, S. (2014). Emotion perception accuracy and bias in face-to-face versus cyberbullying. *The Journal of Genetic Psychology: Research and Theory on Human Development, 175*(5), 382–400.

5961 Clausi, S., Olivito, G., Siciliano, L., Lupo, M., Laghi, F., Baiocco, R., & Leggio, M. (2021). The cerebellum is linked to theory of mind alterations in autism. A direct clinical and MRI comparison between individuals with autism and cerebellar neurodegenerative pathologies. *Autism Research, 14*(11), 2300–2313.
254 Colasante, T., Jambon, M., Gao, X., & Malti, T. (2021). A process model linking physiological arousal and fear recognition to aggression via guilt in middle childhood. *Development and Psychopathology, 33*(1), 109–121.
7392 Coleman, J. C. (1949). Facial expressions of emotion. *Psychological Monographs: General and Applied, 63*(1), i–36.
821 Colonnello, V., Russo, P. M., & Mattarozzi, K. (2019). First impression misleads emotion recognition. *Frontiers in Psychology, 10,* Article 527.
798 Connolly, H. L., Lefevre, C. E., Young, A. W., & Lewis, G. J. (2019). Sex differences in emotion recognition: Evidence for a small overall female superiority on facial disgust. *Emotion, 19*(3), 455–464.
6663 Cook, C. M., & Saucier, D. M. (2010). Mental rotation, targeting ability and Baron-Cohen's Empathizing–Systemizing theory of sex differences. *Personality and Individual Differences, 49*(7), 712–716.
343 Cordaro, D. T., Sun, R., Kamble, S., Hodder, N., Monroy, M., Cowen, A., Bai, Y., & Keltner, D. (2020). The recognition of 18 facial-bodily expressions across nine cultures. *Emotion, 20*(7), 1292–1300.
6621 Cotler, T. B. (2012). The relationship among attachment patterns, mentalization, and empathy. *Dissertation Abstracts International: Section B: The Sciences and Engineering, 73*(5-B), 3259.
6161 Crenshaw, A. O., Leo, K., & Baucom, B. R. W. (2019). The effect of stress on empathic accuracy in romantic couples. *Journal of Family Psychology, 33*(3), 327–337.
5911 Cross, L., Piovesan, A., & Atherton, G. (2022). Autistic people outperform neurotypicals in a cartoon version of the Reading the Mind in the Eyes. *Autism Research, 15*(9), 1603–1608.
207 Cruz, B. F., de Campos-Carli, S. M., de Oliveira, A. M., de Brito, C. B., Garcia, Z. M., do Nascimento Arifa, R. D., de Souza, D. d. G., Teixeira, A. L., & Salgado, J. V. (2021). Investigating potential associations between neurocognition/social cognition and oxidative stress in schizophrenia. *Psychiatry Research, 298,* Article 113832.
174 Cuve, H. C., Castiello, S., Shiferaw, B., Ichijo, E., Catmur, C., & Bird, G. (2021). Alexithymia atypical spatiotemporal dynamics of eye gaze in autism. *Cognition, 212,* Article 104710.
6870 Czernecki, V., Benchetrit, E., Houot, M., Pineau, F., Mangone, G., Corvol, J.-C., Vidailhet, M., & Levy, R. (2021). Social cognitive impairment in early Parkinson's disease: A novel "mild impairment"? *Parkinsonism & Related Disorders, 85,* 117–121.
1001 da Costa, H. P., Vrabel, J. K., Zeigler-Hill, V., & Vonk, J. (2018). DSM-5 pathological personality traits are associated with the ability to understand the emotional states of others. *Journal of Research in Personality, 75,* 1–11.
6169 Dalkner, N., Bengesser, S. A., Birner, A., Fellendorf, F. T., Hamm, C., Platzer, M., Pilz, R., Queissner, R., Rieger, A., Weber, B., Kapfhammer, H. P., Weiss, E. M., & Reininghaus, E. Z. (2019). The relationship between "Eyes Reading" ability and verbal memory in bipolar disorder. *Psychiatry Research, 273,* 42–51.
7622 da Motta, C., Carvalho, C. B., Castilho, P., & Pato, M. T. (2021). Assessment of neurocognitive function and social cognition with computerized batteries: Psychometric properties of the Portuguese PennCNB in healthy controls. *Current Psychology: A Journal for Diverse Perspectives on Diverse Psychological Issues, 40*(10), 4851–4862.
7450 da Motta, C., Castilho, P., Pato, M. T., & Barreto Carvalho, C. (2022). Rasch model analysis of the Situational Test of Emotional Understanding—Brief in a large Portuguese sample. *Current Psychology: A Journal for Diverse Perspectives on Diverse Psychological Issues, 41*(6), 3853–3864.
5672 Danaher, H., Allott, K., Killackey, E., Hester, R., & Cotton, S. (2018). An examination of sex differences in neurocognition and social cognition in first-episode psychosis. *Psychiatry Research, 259,* 36–43.
360 Dark, F., Scott, J. G., Baker, A., Parker, S., Gordon, A., Newman, E., Gore-Jones, V., Lim, C. C. W., Jones, L., & Penn, D. L. (2020). Randomized controlled trial of social cognition and interaction training compared to befriending group. *British Journal of Clinical Psychology, 59*(3), 384–402.
799 Darke, H., Cropper, S. J., & Carter, O. (2019). A novel dynamic morphed stimuli set to assess sensitivity to identity and emotion attributes in faces. *Frontiers in Psychology, 10,* Article 757.
1108 Davidson, C. A., Lesser, R., Parente, L. T., & Fiszdon, J. M. (2018). Psychometrics of social cognitive measures for psychosis treatment research. *Schizophrenia Research, 193,* 51–57.
5930 De Mulder, H. N. M., Hakemulder, F., Klaassen, F., Junge, C. M. M., Hoijtink, H., & van Berkum, J. J. A. (2022). Figuring out what they feel: Exposure to eudaimonic narrative fiction is related to mentalizing ability. *Psychology of Aesthetics, Creativity, and the Arts, 16*(2), 242–258.

6253 De Mulder, H. N. M., Hakemulder, F., van den Berghe, R., Klaassen, F., & van Berkum, J. J. A. (2017). Effects of exposure to literary narrative fiction: From book smart to street smart? *Scientific Study of Literature, 7*(1), 129–169.
116 de Gracia, M. R. L., de Rosnay, M., Hawes, D. J., & Perez, M. V. T. (2021). Differences in emotion knowledge among Filipino deaf children, adolescents, and young adults. *Personality and Individual Differences, 180,* Article 110967.
6498 Dehning, S., Reiß, E., Krause, D., Gasperi, S., Meyer, S., Dargel, S., Müller, N., & Siebeck, M. (2014). Empathy in high-tech and high-touch medicine. *Patient Education and Counseling, 95*(2), 259–264.
2695 Delancey, C. L. (2006). What is in a face? hedonic tone judgments and behavioral inclinations in response to emerging emotional facial expressions. *Dissertation Abstracts International: Section B: The Sciences and Engineering, 67*(5-B), 2874.
6446 Demers, L. A., & Koven, N. S. (2015). The relation of alexithymic traits to affective theory of mind. *The American Journal of Psychology, 128*(1), 31–42.
703 De Panfilis, C., Antonucci, C., Meehan, K. B., Cain, N. M., Soliani, A., Marchesi, C., Clarkin, J. F., & Sambataro, F. (2019). Facial emotion recognition and social-cognitive correlates of narcissistic features. *Journal of Personality Disorders, 33*(4), 433–449.
5039 Demenescu, L. R., Mathiak, K. A., & Mathiak, K. (2014). Age- and gender-related variations of emotion recognition in pseudowords and faces. *Experimental Aging Research, 40*(2), 187–207.
594 Dentakos, S., Saoud, W., Ackerman, R., & Toplak, M. E. (2019). Does domain matter? Monitoring accuracy across domains. *Metacognition and Learning, 14*(3), 413–436.
6418 Derks, J., Van Scheppingen, M. A., Lee, N. C., & Krabbendam, L. (2015). Trust and mindreading in adolescents: The moderating role of social value orientation. *Frontiers in Psychology, 6,* Article 965.
922 de Souza, L. C., Bertoux, M., de Faria, Â. R. V., Corgosinho, L. T. S., Prado, A. C. d. A., Barbosa, I. G., Caramelli, P., Colosimo, E., & Teixeira, A. L. (2018). The effects of gender, age, schooling, and cultural background on the identification of facial emotions: A transcultural study. *International Psychogeriatrics, 30*(12), 1861–1870.
53 Deveney, C. M., Chavez, G., & Mejia, L. (2022). Trait irritability in adults is unrelated to face emotion identification. *Personality and Individual Differences, 185,* Article 111290.
732 Deveney, C. M., Stoddard, J., Evans, R. L., Chavez, G., Harney, M., & Wulff, R. A. (2019). On defining irritability and its relationship to affective traits and social interpretations. *Personality and Individual Differences, 144,* 61–67.
888 Dewaele, J.-M., Lorette, P., & Petrides, K. V. (2019). The effects of linguistic proficiency, trait emotional intelligence and in-group advantage on emotion recognition by British and American English L1 users. In J. L. Mackenzie & L. Alba-Juez (Eds.), *Emotion in discourse* (pp. 279–299). John Benjamins Publishing Company.
985 Dewangan, R. L., & Singh, P. (2018). Premorbid adjustment in predicting symptom severity and social cognitive deficits in schizophrenia. *East Asian Archives of Psychiatry, 28*(3), 75–79.
279 Dietze, P., & Knowles, E. D. (2021). Social class predicts emotion perception and perspective-taking performance in adults. *Personality and Social Psychology Bulletin, 47*(1), 42–56.
830 Di Girolamo, M., Giromini, L., Winters, C. L., Serie, C. M. B., & de Ruiter, C. (2019). The Questionnaire of Cognitive and Affective Empathy: A comparison between paper-and-pencil versus online formats in Italian samples. *Journal of Personality Assessment, 101*(2), 159–170.
7524 Dimitrovsky, L. The ability to identify the emotional meaning of vocal expressions at successive age levels. In J. R. Davitz (Ed.), *The communication of emotional meaning*. McGraw-Hill, 1964.
6949 Dimitrovsky, L., Spector, H., & Levy-Shiff, R. (2000). Stimulus gender and emotional difficulty level: Their effect on recognition of facial expressions of affect in children with and without LD. *Journal of Learning Disabilities, 33*(5), 410–416.
391 Di Tella, M., Miti, F., Ardito, R. B., & Adenzato, M. (2020). Social cognition and sex: Are men and women really different? *Personality and Individual Differences, 162,* Article 110045.
6103 Dodell-Feder, D., Ressler, K. J., & Germine, L. T. (2020). Social cognition or social class and culture? On the interpretation of differences in social cognitive performance. *Psychological Medicine, 50*(1), 133–145.
280 Dodich, A., Crespi, C., Santi, G. C., Luzzi, S., Ranaldi, V., Iannaccone, S., Marcone, A., Zamboni, M., Cappa, S. F., & Cerami, C. (2021). Diagnostic accuracy of affective social tasks in the clinical classification between the behavioral variant of frontotemporal dementia and other neurodegenerative disease. *Journal of Alzheimer's Disease, 80*(4), 1401–1411.

595 Donaldson, P. H., Kirkovski, M., Rinehart, N. J., & Enticott, P. G. (2019). A double-blind HD-tDCS/EEG study examining right temporoparietal junction involvement in facial emotion processing. *Social Neuroscience, 14*(6), 681–696.
1667 Donhauser, P. W., Rösch, A. G., & Schultheiss, O. C. (2015). The implicit need for power predicts recognition speed for dynamic changes in facial expressions of emotion. *Motivation and Emotion, 39*(5), 714–721.
6318 Đorđević, J., Živanović, M., Pavlović, A., Mihajlović, G., Karličić, I. S., & Pavlović, D. (2017). Psychometric evaluation and validation of the Serbian version of "Reading the Mind in the Eyes" test. *Psihologija, 50*(4), 483–502.
5898 Dorris, L., Young, D., Barlow, J., Byrne, K., & Hoyle, R. (2022). Cognitive empathy across the lifespan. *Developmental Medicine & Child Neurology, 64*(12), 1524–1531.
7403 Dusenbury, D., & Knower, F. H. (1938). Experimental studies of the symbolism of action and voice. I. A study of the specificity of meaning in facial expression. *Quarterly Journal of Speech, 24,* 424–435.
7404 Dusenbury, D, & Knower, F. H. (1939). Experimental studies of the symbolism of action and voice-II: A study of the specificity of meaning in abstract tonal symbols. *Quarterly Journal of Speech, 25*(1), 67-75.
7199 Eckland, N. S., & English, T. (2019). Trait-level emotion regulation and emotional awareness predictors of empathic accuracy. *Motivation and Emotion, 43*(3), 461–470.
7181 Eckland, N. S., Huang, A. B., & Berenbaum, H. (2020). Empathic accuracy: Associations with prosocial behavior and self-insecurity. *Emotion, 20*(7), 1306–1310.
7208 Eckland, N. S., Leyro, T. M., Mendes, W. B., & Thompson, R. J. (2018). A multi-method investigation of the association between emotional clarity and empathy. *Emotion, 18*(5), 638–645.
6067 Eddy, C. M., Hansen, P. C., & Pavlova, M. A. (2020). Predictors of performance on the Reading the Mind in the Eyes Test. *PLoS ONE, 15*(7), Article e0235529.
7408 Elfenbein, H. A., & Ambady, N. (2003). When familiarity breeds accuracy: Cultural exposure and facial emotion recognition. *Journal of Personality and Social Psychology, 85*(2), 276–290.
2622 Elfenbein, H. A., Foo, M. D., White, J., Tan, H. H., & Aik, V. C. (2007). Reading your counterpart: The benefit of emotion recognition accuracy for effectiveness in negotiation. *Journal of Nonverbal Behavior, 31*(4), 205–223.
7050 Elfenbein, H. A., Mandal, M. K., Ambady, N., Harizuka, S., & Kumar, S. (2002). Cross-cultural patterns in emotion recognition: Highlighting design and analytical techniques. *Emotion, 2*(1), 75–84.
5917 Eramudugolla, R., Huynh, K., Zhou, S., Amos, J. G., & Anstey, K. J. (2022). Social cognition and social functioning in MCI and dementia in an epidemiological sample. *Journal of the International Neuropsychological Society, 28*(7), 661–672.
859 Etchepare, A., Roux, S., Destaillats, J.-M., Cady, F., Fontanier, D., Couhet, G., & Prouteau, A. (2019). What are the specificities of social cognition in schizophrenia? A cluster-analytic study comparing schizophrenia with the general population. *Psychiatry Research, 272,* 369–379.
478 Evans, T. R., Hughes, D. J., & Steptoe-Warren, G. (2020). A conceptual replication of emotional intelligence as a second-stratum factor of intelligence. *Emotion, 20*(3), 507–512.
6365 Ewing, D., Zeigler-Hill, V., & Vonk, J. (2016). Spitefulness and deficits in the social–perceptual and social–cognitive components of Theory of Mind. *Personality and Individual Differences, 91,* 7–13.
5680 Eyal, T., & Epley, N. (2017). Exaggerating accessible differences: When gender stereotypes overestimate actual group differences. *Personality and Social Psychology Bulletin, 43*(9), 1323–1336.
5671 Eyal, T., Steffel, M., & Epley, N. (2018). Perspective mistaking: Accurately understanding the mind of another requires getting perspective, not taking perspective. *Journal of Personality and Social Psychology, 114*(4), 547–571.
890 Faber, A., & Walter, F. (2019). Power and emotion recognition: The moderating role of work stress. In N. M. Ashkanasy, W. J. Zerbe, & C. E. J. Härtel (Eds.), *Emotions and leadership* (pp. 3–20). Emerald Publishing.
6170 Fahlgren, M. K., Puhalla, A. A., Sorgi, K. M., & McCloskey, M. S. (2019). Emotion processing in intermittent explosive disorder. *Psychiatry Research, 273,* 544–550.
9005 Falkenberg, L., Hagelstein, T., Hecht, T., Heinzelmann, E., Kalteis, S., KÃ¶lling, E., Kulla, K., Langerbeck, L., Manns, S., Spielmann, M., Springer, U., StÃ¤blein, S., Yumusak, M., & Kappes, C. (2023). Unpublished data, University of Hildesheim.
5020 Fallis, E. E., Rehman, U. S., & Purdon, C. (2014). Perceptions of partner sexual satisfaction in heterosexual committed relationships. *Archives of Sexual Behavior, 43*(3), 541–550.
130 Farhoumandi, N., Mollaey, S., Heysieattalab, S., Zarean, M., & Eyvazpour, R. (2021). Facial emotion recognition predicts alexithymia using machine learning. *Computational Intelligence and Neuroscience, 2021,* Article 2053795.

5909 Fekete, Z., Vass, E., Balajth, R., Tana, Ü., Nagy, A. C., Domján, N., Égerházi, A., & Kuritárné, I. S. (2022). Regrouping scalets: Psychometric properties of the theory of mind picture stories task in a schizophrenic sample. *Neuropsychological Rehabilitation, 32*(9), 2227–2247.
1024 Felisberti, F. M. (2018). Long-lasting effects of family-related factors on adults' ability to recognise brief facial expressions of emotion. *The Quarterly Journal of Experimental Psychology, 71*(7), 1512–1525.
7506 Ferguson, F. J., & Austin, E. J. (2010). Associations of trait and ability emotional intelligence with performance on Theory of Mind tasks in an adult sample. *Personality and Individual Differences, 49*(5), 414–418.
556 Fernández-Modamio, M., Gil-Sanz, D., Arrieta-Rodríguez, M., de Tojeiro-Roce, J. G., Bengochea-Seco, R., & González-Fraile, E. (2020). Emotion recognition in patients with schizophrenia: The role of sex. *Psicothema, 32*(2), 197–203.
268 Fernández-Sotos, P., García, A. S., Vicente-Querol, M. A., Lahera, G., Rodriguez-Jimenez, R., & Fernández-Caballero, A. (2021). Validation of dynamic virtual faces for facial affect recognition. *PLoS ONE, 16*(1), Article e0246001
6044 Ferrer, I., Alacreu-Crespo, A., Salvador, A., Genty, C., Dubois, J., Sénèque, M., Courtet, P., & Olié, E. (2020). I cannot read your eye expression: Suicide attempters have difficulties in interpreting complex social emotions. *Frontiers in Psychiatry, 11,* Article 543889.
8000 Ferrer-Quintero, Green, M. F., Horan, W. P., Penn, D. L., Kern, R. S., & Lee, J. (2021) The effect of sex on social cognition and functioning in schizophrenia. *npj Schizophrenia, 7*:57.
7580 Fiori, M., & Antonakis, J. (2011). The ability model of emotional intelligence: Searching for valid measures. *Personality and Individual Differences, 50*(3), 329–334.
1348 Fisher, M., Nahum, M., Howard, E., Rowlands, A., Brandrett, B., Kermott, A., Woolley, J., & Vinogradov, S. (2017). Supplementing intensive targeted computerized cognitive training with social cognitive exercises for people with schizophrenia: An interim report. *Psychiatric Rehabilitation Journal, 40*(1), 21–32.
448 Fittipaldi, S., Abrevaya, S., de la Fuente, A., Pascariello, G. O., Hesse, E., Birba, A., Salamone, P., Hildebrandt, M., Martí, S. A., Pautassi, R. M., Huepe, D., Martorell, M. M., Yoris, A., Roca, M., García, A. M., Sedeño, L., & Ibáñez, A. (2020). A multidimensional and multi-feature framework for cardiac interoception. *NeuroImage, 212,* Article 116677.
6091 Floyd, K., & Woo, N. T. (2020). Loneliness and social monitoring: A conceptual replication of Knowles et al. *Personal Relationships, 27*(1), 209–223.
6257 Fossati, A., Somma, A., Krueger, R. F., Markon, K. E., & Borroni, S. (2017). On the relationships between *DSM-5* dysfunctional personality traits and social cognition deficits: A study in a sample of consecutively admitted Italian psychotherapy patients. *Clinical Psychology & Psychotherapy, 24*(6), 1421–1434.
7409 Francis, P. L., Lombardo, J. P., & Simon, L. J. (1987). Relation of sex and sex role to adults' ability to decode infants' emotions. *Perceptual and Motor Skills, 65*(2), 595–600.
5732 Franklin, R. G., Jr., & Adams, R. B., Jr. (2010). What makes a face memorable? The relationship between face memory and emotional state reasoning. *Personality and Individual Differences, 49*(1), 8–12.
1833 Franklin, R. G., Jr., Stevenson, M. T., Ambady, N., & Adams, R. B., Jr. (2015). Cross-cultural reading the mind in the eyes and its consequences for international relations. In J. E. Warnick & D. Landis (Eds.), *Neuroscience in intercultural contexts* (pp. 117–141). Springer Science + Business Media.
7516 Frijda, N. H. (1953). The understanding of facial expression of emotion. *Acta Psychologica, 9*, 294- 362.
625 Frøyhaug, M., Andersson, S., Andreassen, O. A., Ueland, T., & Vaskinn, A. (2019). Theory of mind in schizophrenia and bipolar disorder: Psychometric properties of the Norwegian version of the Hinting Task. *Cognitive Neuropsychiatry, 24*(6), 454–469.
7563 Funder, D. C., & Harris, M. J. (1986). On the several facets of personality assessment: The case of social acuity. *Journal of Personality, 54*(3), 528–550.
226 Fundora, C., Cruz, M., Barone, K., Penn, D. L., Jarskog, l. F., Pinkham, A. E., & Harvey, P. D. (2021). Lifetime employment in schizophrenia: Correlates of developing long term unemployment after being employed before. *Cognitive Neuropsychiatry, 26*(2), 95–106.
38 Fusar-Poli, L., Pries, L.-K., van Os, J., Erzin, G., Delespaul, P., Kenis, G., Luykx, J. J., Lin, B. D., Richards, A. L., Akdede, B., Binbay, T., Altınyazar, V., Yalınçetin, B., Gümüş-Akay, G., Cihan, B., Soygür, H., Ulaş, H., Cankurtaran, E. Ş., Kaymak, S. U., . . . Genetic Risk and Outcome of Psychosis (GROUP) Investigators. (2022). Examining facial emotion recognition as an intermediate phenotype for psychosis: Findings from the EUGEI study. *Progress in Neuro-Psychopharmacology & Biological Psychiatry, 113,* Article 110440.
5753 Galinsky, A. D., Magee, J. C., Inesi, M. E., & Gruenfeld, D. H. (2006). Power and perspectives not taken. *Psychological Science, 17*(12), 1068–1074.

7589 Gallois, C., & Callan, V. J. (1986). Decoding emotional messages: Influence of ethnicity, sex, message type, and channel. *Journal of Personality and Social Psychology, 51*, 755–762.
227 Gamsakhurdashvili, D., Antov, M. I., Lübke, K. T., Pause, B. M., & Stockhorst, U. (2021). The role of olfaction and sex-hormone status in empathy-related measures. *Physiology & Behavior, 230,* Article 113289.
835 García-Casal, J. A., Martínez-Abad, F., Cid-Bartolomé, T., Smith, S. J., Llano-Ordóñez, K., Perea-Bartolomé, M. V., Goñi-Imizcoz, M., Soto-Pérez, F., & Franco-Martín, M. (2019). Usability study and pilot validation of a computer-based emotion recognition test for older adults with Alzheimer's disease and amnestic mild cognitive impairment. *Aging & Mental Health, 23*(3), 365–375.
6765 Gentzler, A. L., DeLong, K. L., & Smart, R. (2020). Theater majors compared with nonmajors: Investigating temperament and emotion beliefs, awareness, regulation, and perception. *Psychology of Aesthetics, Creativity, and the Arts, 14*(3), 301–312.
7157 Genzer, S., Ong, D. C., Zaki, J., & Perry, A. (2022). Mu rhythm suppression over sensorimotor regions is associated with greater empathic accuracy. *Social Cognitive and Affective Neuroscience, 17*(9), 788–801.
6426 Germine, L., Dunn, E. C., McLaughlin, K. A., & Smoller, J. W. (2015). Childhood adversity is associated with adult theory of mind and social affiliation, but not face processing. *PLoS ONE, 10*(6), Article e0129612.
7364 Gesn, P. R. (1998). The development of meaning contexts for empathic accuracy: Channel and sequence effects. *Dissertation Abstracts International: Section B: The Sciences and Engineering, 58*(9-B), 5189.
3 Giacomucci, G., Galdo, G., Polito, C., Berti, V., Padiglioni, S., Mazzeo, S., Chiaro, E., De Cristofaro, M. T., Bagnoli, S., Nacmias, B., Sorbi, S., & Bessi, V. (2022). Unravelling neural correlates of empathy deficits in subjective cognitive decline, mild cognitive impairment and Alzheimer’s disease. *Behavioural Brain Research, 428,* 1–12.
7573 Gilbert, P., Catarino, F., Sousa, J., Ceresatto, L., Moore, R., & Basran, J. (2017). Measuring competitive self-focus perspective taking, submissive compassion and compassion goals. *Journal of Compassionate Health Care, 4*, 1–9.
5678 Gillen, C. T. A., Lee, Z., Salekin, K. L., Iselin, A.-M. R., Harrison, N. A., Clark, A. P., Colins, O. F., & Salekin, R. T. (2018). Psychopathic traits in adolescence: The importance of examining components in face processing, voice processing, and emotional skill. *Journal of Psychopathology and Behavioral Assessment, 40*(1), 50–59.
893 Giordano, M., Licea-Haquet, G., Navarrete, E., Valles-Capetillo, E., Lizcano-Cortés, F., Carrillo-Peña, A., & Zamora-Ursulo, A. (2019). Comparison between the Short Story Task and the Reading the Mind in the Eyes Test for evaluating Theory of Mind: A replication report. *Cogent Psychology, 6*(1), Article 1634326.
6484 Girli, A. (2014). Psychometric properties of the Turkish child and adult form of "Reading the Mind in the Eyes Test." *Psychology, 5*(11), 1321–1337.
7414 Gitter, A. G., Kozel, N. J., & Mostofsky, D. I. (1972). Perception of emotion: The role of race, sex, and presentation mode. *The Journal of Social Psychology, 88*(2), 213–222.
1835 Glahn, D. C., Williams, J. T., McKay, D. R., Knowles, E. E., Sprooten, E., Mathias, S. R., Curran, J. E., Kent, J. W., Jr., Carless, M. A., Göring, H. H. H., Dyer, T. D., Woolsey, M. D., Winkler, A. M., Olvera, R. L., Kochunov, P., Fox, P. T., Duggirala, R., Almasy, L., & Blangero, J. (2015). Discovering schizophrenia endophenotypes in randomly ascertained pedigrees. *Biological Psychiatry, 77*(1), 75–83.
5148 Goddard, E., & Treasure, J. (2013). Anxiety and social-emotional processing in eating disorders: Examination of family trios. *Cognitive Therapy and Research, 37*(5), 890–904.
7564 Goldstein, N. E., & Feldman, R. S. (1996). Knowledge of American Sign Language and the ability of hearing individuals to decode facial expressions of emotion. *Journal of Nonverbal Behavior, 20*(2), 111–122.
6701 Goldstein, T. R., Wu, K., & Winner, E. (2009). Actors are skilled in theory of mind but not empathy. *Imagination, Cognition and Personality, 29*(2), 115–133.
6474 Gong, P., Liu, J., Li, S., & Zhou, X. (2014). Dopamine beta-hydroxylase gene modulates individuals empathic ability. *Social Cognitive and Affective Neuroscience, 9*(9), 1341–1345.
6675 Gooding, D. C., Johnson, M., & Peterman, J. S. (2010). Schizotypy and altered digit ratios: A second look. *Psychiatry Research, 178*(1), 73–78.
1720 Goodkind, M. S., Sturm, V. E., Ascher, E. A., Shdo, S. M., Miller, B. L., Rankin, K. P., & Levenson, R. W. (2015). Emotion recognition in frontotemporal dementia and Alzheimer’s disease: A new film-based assessment. *Emotion, 15*(4), 416–427.
7367 Graham, T. M. (1997). The effects of feedback and frame on empathic accuracy and metaknowledge. *Dissertation Abstracts International: Section B: The Sciences and Engineering, 57*(10-B), 6649.
6199 Grainger, S. A., Henry, J. D., Naughtin, C. K., Comino, M. S., & Dux, P. E. (2018). Implicit false belief tracking is preserved in late adulthood. *The Quarterly Journal of Experimental Psychology, 71*(9), 1980–1987.

1116 Grainger, S. A., Henry, J. D., Steinvik, H. R., Vanman, E. J., Rendell, P. G., & Labuschagne, I. (2018). Intranasal oxytocin does not reduce age-related difficulties in social cognition. *Hormones and Behavior, 99,* 25–34.

209 Grainger, S. A., Mead, J. K., Vanman, E. J., & Henry, J. D. (2021). The relationship between testosterone and social cognition in younger and older adults. *Biological Psychology, 161,* Article 108072.

6025 Grainger, S. A., Rakunathan, V., Adams, A. G., Canty, A. L., & Henry, J. D. (2021). An assessment of age differences in theory of mind using the virtual assessment of mentalizing ability. *Aging, Neuropsychology, and Cognition, 28*(1), 97–107.

2046 Grant, P. M., & Beck, A. T. (2010). Asocial beliefs as predictors of asocial behavior in schizophrenia. *Psychiatry Research, 177*(1-2), 65–70.

9009 Greenberg, D. M., Warrier, V., Abu-Akeld, A., Allison, C., Gajosf, K. Z., Reinecke, K., Rentfrow, P. J., Radecki, M. A., & Baron-Cohen, S. (2023). Sex and age differences in "theory of mind" across 57 countries using the English version of the "Reading the Mind in the Eyes" Test. *PNAS, 120*(1), e2022385119.

6114 Greenway, T. S., Jin, J., Shepherd, A. M., & Schnitker, S. A. (2019). Beyond the liberal–conservative binary: Generosity, religion, and a latent profile analysis of moral foundations in a Christian sample. *American Behavioral Scientist, 63*(14), 1938–1964.

7507 Grisham, J. R., Henry, J. D., Williams, A. D., & Bailey, P. E. (2010). Socioemotional deficits associated with obsessive–compulsive symptomatology. *Psychiatry Research, 175*(3), 256–259.

203 Grundmann, F., Epstude, K., & Scheibe, S. (2021). Face masks reduce emotion-recognition accuracy and perceived closeness. *PLoS ONE, 16*(4), Article e0249792.

6432 Guariglia, P., Piccardi, L., Giaimo, F., Alaimo, S., Miccichè, G., & Antonucci, G. (2015). The eyes test is influenced more by artistic inclination and less by sex. *Frontiers in Human Neuroscience, 9,* Article 292.

5606 Guidetti, M. (1991). L'expression vocale des émotions: approche interculturelle et développementale [Vocal expression of emotions: A crosscultural and developmental approach]. *L'Année Psychologique, 91*(3), 383–396.

5996 Gülbetekin, E., Altun, E., Er, M. N., Fidancı, A., Keskin, P., & Steenken, D. (2021). Effects of right or left face stimulation on self and other perception in enfacement illusion. *Social Neuroscience, 16*(2), 189–205.

7574 Gummelt, H. (2015). Does gender role moderate the relationship between empathy and psychopathy? *Dissertation Abstracts International: Section B: The Sciences and Engineering, 76*(2-B(E)).

7808 Gur, R. C., Richard, J., Calkins, M. E., Chiavacci, R., Hansen, J. A., Bilker, W. B., Loughead, J., Connolly, J. J., Qiu, H., Mentch, F. D., Abou-Sleiman, P. M., Hakonarson, H., & Gur, R. E. (2012). Age group and sex differences in performance on a computerized neurocognitive battery in children age 8−21. *Neuropsychology, 26*(2), 251–265.

7834 Gur, R. C., Richard, J., Hughett, P., Calkins, M. E., Macy, L., Bilker, W. B., Brensinger, C., Gur, R. E. (2010). A cognitive neuroscience-based computerized battery for efficient measurement of individual differences: Standardization and initial construct validation. *Journal of Neuroscience Methods, 187*(2), 254-262.

1213 Guy, A., Lee, K., & Wolke, D. (2017). Differences in the early stages of social information processing for adolescents involved in bullying. *Aggressive Behavior, 43*(6), 578–587.

1508 Haas, B. W., Filkowski, M. M., Cochran, R. N., Denison, L., Ishak, A., Nishitani, S., & Smith, A. K. (2016). Epigenetic modification of OXT and human sociability. *PNAS Proceedings of the National Academy of Sciences of the United States of America, 113*(27), E3816–E3823.

18 Haining, K., Gajwani, R., Gross, J., Gumley, A. I., Ince, R. A. A., Lawrie, S. M., Schultze-Lutter, F., Schwannauer, M., & Uhlhaas, P. J. (2022). Characterising cognitive heterogeneity in individuals at clinical high-risk for psychosis: A cluster analysis with clinical and functional outcome prediction. *European Archives of Psychiatry and Clinical Neuroscience, 272*(3), 437–448.

509 Hajdúk, M., Klein, H. S., Bass, E. L., Springfield, C. R., & Pinkham, A. E. (2020). Implicit and explicit processing of bodily emotions in schizophrenia. *Cognitive Neuropsychiatry, 25*(2), 139–153.

5791 Hall, C. W., Gaul, L., & Kent, M. (1999). College students' perception of facial expressions. *Perceptual and Motor Skills, 89*(3, Pt 1), 763–770.

9008 Hall, J. A. (2018). Unpublished data, Northeastern University.

6992 Hall, J. A., Halberstadt, A. G., & O'Brien, C. E. (1997). "Subordination" and nonverbal sensitivity: A study and synthesis of findings based on trait measures. *Sex Roles: A Journal of Research, 37*(5-6), 295–317.

8005 Hall, J. A., & Matsumoto, D. (2004). Gender differences in judgments of multiple emotions from facial expressions. *Emotion, 4*(2), 201–206.

2511 Hall, J. A., Roter, D. L., Blanch, D. C., & Frankel, R. M. (2009). Nonverbal sensitivity in medical students: Implications for clinical interactions. *Journal of General Internal Medicine, 24*(11), 1217–1222.

5069 Hall, J. A., Ship, A. N., Ruben, M. A., Curtin, E. M., Roter, D. L., Clever, S. L., Smith, C. C., & Pounds, K. (2014). The Test of Accurate Perception of Patients' Affect (TAPPA): An ecologically valid tool for assessing interpersonal perception accuracy in clinicians. *Patient Education and Counseling, 94*(2), 218–223.

7420 Hall, J. K., Hutton, S. B., & Morgan, M. J. (2010). Sex differences in scanning faces: Does attention to the eyes explain female superiority in facial expression recognition? *Cognition and Emotion, 24*(4), 629–637.

6700 Hallerbäck, M. U., Lugnegård, T., Hjärthag, F., & Gillberg, C. (2009). The Reading the Mind in the Eyes Test: Test-retest reliability of a Swedish version. *Cognitive Neuropsychiatry, 14*(2), 127–143.

6275 Hamilton, J., Radlak, B., Morris, P. G., & Phillips, L. H. (2017). Theory of mind and executive functioning following stroke. *Archives of Clinical Neuropsychology, 32*(5), 507–518.

229 Hampson, E., Istasy, P., Owais, S., Chow, J. A., Howidi, B., & Ouellette, S. J. (2021). Sex differences in the recognition of children's emotional expressions: A test of the fitness threat hypothesis. *Evolutionary Psychological Science, 7*(1), 45–60.

6026 Hart, W., Breeden, C. J., & Kinrade, C. (2021). Re-conceptualizing Machiavellianism and social-cognitive skills: Machiavellianism blends deficient, proficient, and average social-cognitive skills. *Journal of Individual Differences, 42*(3), 140–147.

894 Hartling, C., Fan, Y., Weigand, A., Trilla, I., Gärtner, M., Bajbouj, M., Dziobek, I., & Grimm, S. (2019). Interaction of HPA axis genetics and early life stress shapes emotion recognition in healthy adults. *Psychoneuroendocrinology, 99,* 28–37.

685 Hasson-Ohayon, I., Mashiach-Eizenberg, M., Lavi-Rotenberg, A., Brüne, M., & Roe, D. (2019). Emotion identification among people with serious mental illnesses: The role of specific emotions and sex. *Psychiatry Research, 279,* 378–379.

6272 Hayward, E. O., & Homer, B. D. (2017). Reliability and validity of advanced theory-of-mind measures in middle childhood and adolescence. *British Journal of Developmental Psychology, 35*(3), 454–462.

5979 Healy, C. J., Lee, K. A., & D'Andrea, W. (2021). Using psychedelics with therapeutic intent is associated with lower shame and complex trauma symptoms in adults with histories of child maltreatment. *Chronic Stress, 5,* Article 24705470211029881.

1673 Hennion, S., Szurhaj, W., Duhamel, A., Lopes, R., Tyvaert, L., Derambure, P., & Delbeuck, X. (2015). Characterization and prediction of the recognition of emotional faces and emotional bursts in temporal lobe epilepsy. *Journal of Clinical and Experimental Neuropsychology, 37*(9), 931–945.

40 Henry, A., Lannoy, S., Chaunu, M.-P., Tourbah, A., & Montreuil, M. (2022). Social cognition and executive functioning in multiple sclerosis: A cluster-analytic approach. *Journal of Neuropsychology, 16*(1), 97–115.

231 Herr, N. R., & Meier, E. P. (2021). Accuracy for subtle facial emotional expressions among individuals with borderline personality disorder symptoms and diagnoses. *Personality Disorders: Theory, Research, and Treatment, 12*(2), 150–159.

8015 Hertenstein, M. J., Keltner, D., App, B., Bulleit, B. A., & Jaskolka, A. R. (2006). Touch communicates distinct emotions. *Emotion, 6*(3), 528–533.

7172 Hodges, S. D., & Kezer, M. (2021). It is hard to read minds without words: Cues to use to achieve empathic accuracy. *Journal of Intelligence, 9*(2), Article 27.

7003 Hodgins, H. S., & Koestner, R. (1993). The origins of nonverbal sensitivity. *Personality and Social Psychology Bulletin, 19*(4), 466–473.

2009 Hoffmann, H., Kessler, H., Eppel, T., Rukavina, S., & Traue, H. C. (2010). Expression intensity, gender and facial emotion recognition: Women recognize only subtle facial emotions better than men. *Acta Psychologica, 135*(3), 278–283.

7245 Hogenelst, K., Schoevers, R. A., Kema, I. P., Sweep, F. C. G. J., & Rot, M. (2016). Empathic accuracy and oxytocin after tryptophan depletion in adults at risk for depression. *Psychopharmacology, 233*(1), 111–120.

5698 Holbein, C. E., Lennon, J. M., Kolbuck, V. D., Zebracki, K., Roache, C. R., & Holmbeck, G. N. (2015). Observed differences in social behaviors exhibited in peer interactions between youth with spina bifida and their peers: Neuropsychological correlates. *Journal of Pediatric Psychology, 40*(3), 320–335.

1882 Holt, R. J., Chura, L. R., Lai, M.-C., Suckling, J., von dem Hagen, E., Calder, A. J., Bullmore, E. T., Baron-Cohen, S., & Spencer, M. D. (2014). 'Reading the Mind in the Eyes': An fMRI study of adolescents with autism and their siblings. *Psychological Medicine, 44*(15), 3215–3227.

1579 Honan, C. A., McDonald, S., Sufani, C., Hine, D. W., & Kumfor, F. (2016). The awareness of social inference test: Development of a shortened version for use in adults with acquired brain injury. *The Clinical Neuropsychologist, 30*(2), 243–264.

988 Honan, C. A., Skromanis, S., Johnson, E. G., & Palmer, M. A. (2018). Alcohol intoxication impairs recognition of fear and sadness in others and metacognitive awareness of emotion recognition ability. *Emotion, 18*(6), 842–854.
338 Hone, L. S. E., Scofield, J. E., Bartholow, B. D., & Geary, D. C. (2020). Frequency of recent binge drinking is associated with sex-specific cognitive deficits: Evidence for condition-dependent trait expression in humans. *Evolutionary Psychology, 18*(4), Article 1474704920954445.
197 Hua, A. Y., Wells, J. L., Brown, C. L., & Levenson, R. W. (2021). Emotional and cognitive empathy in caregivers of people with neurodegenerative disease: Relationships with caregiver mental health. *Clinical Psychological Science, 9*(3), 449–466.
1375 Huang, C. L.-C., & Hsiao, S. (2017). The functional significance of affect recognition, neurocognition, and clinical symptoms in schizophrenia. *PLoS ONE, 12*(1), Article e0170114.
862 Huang, Y.-L., Chen, S.-H., & Tseng, H.-H. (2019). Attachment avoidance and fearful prosodic emotion recognition predict depression maintenance. *Psychiatry Research, 272,* 649–654.
6029 Hudson, C. C. (2021). Behavioural manifestations and relational correlates of individual differences in theory of mind. *Dissertation Abstracts International: Section B: The Sciences and Engineering, 82*(11-B).
6074 Hudson, C. C., Shamblaw, A. L., Harkness, K. L., & Sabbagh, M. A. (2020). Valence in the Reading the Mind in the Eyes task. *Psychological Assessment, 32*(7), 623–634.
6553 Hünefeldt, T., Laghi, F., Ortu, F., & Belardinelli, M. O. (2013). The relationship between 'theory of mind' and attachment-related anxiety and avoidance in Italian adolescents. *Journal of Adolescence, 36*(3), 613–62.
7423 Hutchison, A. N., & Gerstein, L. H. (2012). What's in a face? Counseling trainees' ability to read emotions. *Training and Education in Professional Psychology, 6*(2), 100–112.
8017 Hutchison, A., Gerstein, L., & Kasai, M. (2018). A cross-cultural comparison of U.S. and Japanese trainees' emotion-recognition ability. *Japanese Psychological Research, 60*(2), 63–76.
6559 Ibanez, A., Huepe, D., Gempp, R., Gutiérrez, V., Rivera-Rei, A., & Toledo, M. I. (2013). Empathy, sex and fluid intelligence as predictors of theory of mind. *Personality and Individual Differences, 54*(5), 616–621.
1467 Ibrahim, I., Salah, H., El Sayed, H., Mansour, H., Eissa, A., Wood, J., Fathi, W., Tobar, S., Gur, R. C., Gur, R. E., Dickerson, F., Yolken, R. H, El Bahaey, W., & Nimgaonkar, V. (2016). Hepatitis C virus antibody titers associated with cognitive dysfunction in an asymptomatic community-based sample. *Journal of Clinical and Experimental Neuropsychology, 38*(8), 861–868.
628 Ibrahim, I., Tobar, S., Fathi, W., ElSayed, H., Yassein, A., Eissa, A., Elsheshtawy, E., Elboraei, H., Shahda, M., Elwasify, M., Ibrahim, A., Chen, K., Wood, J., Dickerson, F., Yolken, R. H., El Chennawi, F., Gur, R., Gur, R., El Bahaey, W., . . . Mansour, H. (2019). Randomized controlled trial of adjunctive Valproate for cognitive remediation in early course schizophrenia. *Journal of Psychiatric Research, 118,* 66–72.
8008 Ickes, W., Graham, T., Hancock, M., Gesn, P. R., & Mortimer, C. (1995). Empathic accuracy and metaknowledge. Unpublished data, University of Texas at Arlington. (reported in Graham, T., & Ickes, W. (1997). When women's intuition isn't greater than men's. In Ickes, W. (1997), *Empathic accuracy* (117-145). Guilford Press.)
6115 Ilzarbe, D., de la Serna, E., Baeza, I., Rosa, M., Puig, O., Calvo, A., Masias, M., Borras, R., Pariente, J. C., Castro-Fornieles, J., & Sugranyes, G. (2019). The relationship between performance in a theory of mind task and intrinsic functional connectivity in youth with early onset psychosis. *Developmental Cognitive Neuroscience, 40,* Article 100726.
5984 Isaksson, J., Neufeld, J., & Bölte, S. (2021). What's the link between theory of mind and other cognitive abilities – a co-twin control design of neurodevelopmental disorders. *Frontiers in Psychology, 12,* Article 575100.
7544 Isenhart, M. W. (1980). An investigation of the relationship of sex and sex role to the ability to decode nonverbal cues. *Human Communication Research, 6*, 309-18.
5935 Ishii, K., & Kanda, T. (2022). A brief acting experience fosters empathic concern. *Psihologijske Teme, 31*(1), 203–214.
5962 Ishii, T., & Watanabe, K. (2021). Caring about you: The motivational component of mentalizing, not the mental state attribution component, predicts religious belief in Japan. *Religion, Brain & Behavior, 11*(4), 361–370.
6096 Isernia, S., Sokolov, A. N., Fallgatter, A. J., & Pavlova, M. A. (2020). Untangling the ties between social cognition and body motion: Gender impact. *Frontiers in Psychology, 11,* Article 128.
629 Israelashvili, J., Oosterwijk, S., Sauter, D., & Fischer, A. (2019). Knowing me, knowing you: Emotion differentiation in oneself is associated with recognition of others' emotions. *Cognition and Emotion, 33*(7), 1461–1471.

364 Israelashvili, J., Sauter, D., & Fischer, A. (2020). Two facets of affective empathy: Concern and distress have opposite relationships to emotion recognition. *Cognition and Emotion, 34*(6), 1112–1122.
7519 Izard, C. E. (1971). *The face of emotion*. Appleton-Century-Crofts.
1560 Jackson, M. C., & Arlegui-Prieto, M. (2016). Variation in normal mood state influences sensitivity to dynamic changes in emotional expression. *Emotion, 16*(2), 145–149.
5726 Jacobs, R. H., Pine, D. S., Schoeny, M. E., Henry, D. B., Gollan, J. K., Moy, G., Cook, E. H., & Wakschlag, L. S. (2011). Maternal depressive history, teen 5HTTLPR genotype, and the processing of emotional faces: Exploring mechanisms of risk. *Behaviour Research and Therapy, 49*(1), 80–84
6377 Jankowiak-Siuda, K., Baron-Cohen, S., Białaszek, W., Dopierała, A., Kozłowska, A., & Rymarczyk, K. (2016). Psychometric evaluation of the 'Reading the Mind in the Eyes' Test with samples of different ages from a Polish population. *Studia Psychologica, 58*(1), 18–31.
1532 Jenkins, L. M., Kassel, M. T., Gabriel, L. B., Gowins, J. R., Hymen, E. A., Vergés, A., Calamia, M., Crane, N. A., Jacobs, R. H., Ajilore, O., Welsh, R. C., Drevets, W. C., Phillips, M. L., Zubieta, J.-K., & Langenecker, S. A. (2016). Amygdala and dorsomedial hyperactivity to emotional faces in youth with remitted major depression. *Social Cognitive and Affective Neuroscience, 11*(5), 736–745.
7424 Jenness, A. (1932). Differences in the recognition of facial expression of emotion. *Journal of General Psychology, 7,* 192–196.
654 Johansen, T., Jensen, C., Eriksen, H. R., Lyby, P. S., Dittrich, W. H., Holsen, I. N., Jakobsen, H., & Øyeflaten, I. (2019). Occupational rehabilitation is associated with improvements in cognitive functioning. *Frontiers in Psychology, 10,* Article 2233.
325 Johnson, C., Langbehn, K. E., Long, J. D., Moser, D., Cross, S., Gutmann, L., Nopoulos, P. C., & van der Plas, E. (2020). Encoding of facial expressions in individuals with adult-onset myotonic dystrophy type 1. *Journal of Clinical and Experimental Neuropsychology, 42*(9), 932–940.
6030 Johnson, D. A., Knight, D. N., & McHugh, K. (2021). Score reliability and validity evidence for the State-Interpersonal Reactivity Index: A multidimensional assessment of in-session counselor empathy. *Measurement and Evaluation in Counseling and Development, 54*(1), 24–41.
103 Jorna, L. S., Westerhof-Evers, H. J., Khosdelazad, S., Rakers, S. E., van der Naalt, J., Groen, R. J. M., Buunk, A. M., & Spikman, J. M. (2021). Behaviors of concern after acquired brain injury: The role of negative emotion recognition and anger misattribution. *Journal of the International Neuropsychological Society, 27*(10), 1015–1023.
397 Jung, M., Baik, S. Y., Kim, Y., Kim, S., Min, D., Kim, J.-Y., Won, S., & Lee, S.-H. (2020). Empathy and social attribution skills moderate the relationship between temporal lobe Vol. and facial expression recognition ability in schizophrenia. *Clinical Psychopharmacology and Neuroscience, 18*(3), 362–374.
880 Kafetsios, K., & Hess, U. (2019). Seeing mixed emotions: Alexithymia, emotion perception bias, and quality in dyadic interactions. *Personality and Individual Differences, 137,* 80–85.
56 Kafetsios, K., & Hess, U. (2022). Personality and the accurate perception of facial emotion expressions: What is accuracy and how does it matter? *Emotion, 22*(1), 100–114.
6046 Kallitsounaki, A., & Williams, D. (2020). Mentalising moderates the link between autism traits and current gender dysphoric features in primarily non-autistic, cisgender individuals. *Journal of Autism and Developmental Disorders, 50*(11), 4148–4157.
5993 Kallitsounaki, A., Williams, D. M., & Lind, S. E. (2021). Links between autistic traits, feelings of gender dysphoria, and mentalising ability: Replication and extension of previous findings from the general population. *Journal of Autism and Developmental Disorders, 51*(5), 1458–1465.
1315 Kaltwasser, L., Hildebrandt, A., Wilhelm, O., & Sommer, W. (2017). On the relationship of emotional abilities and prosocial behavior. *Evolution and Human Behavior, 38*(3), 298–308.
7425 Kanner, L. (1931). Judging emotions from facial expressions. *Psychological Monographs, 41*(3), i–91.
6297 Karthikeyan, S., & Ramachandra, V. (2017). Social eavesdropping: Can you hear the emotionality in a "hello" that is not meant for you? *i-Perception, 8*(2), Article 2041669517695816.
7130 Keeley-Dyreson, M., Burgoon, J. K., & Bailey, W. (1991). The effects of stress and gender on nonverbal decoding accuracy in kinesic and vocalic channels. *Human Communication Research, 17*(4), 584–605.
57 Kelly, M., McDonald, S., & Wallis, K. (2022). Empathy across the ages: "I may be older but I'm still feeling it". *Neuropsychology, 36*(2), 116–127.
187 Kessels, R. P. C., Elferink, M. W.-O., & van Tilborg, I. (2021). Social cognition and social functioning in patients with amnestic mild cognitive impairment or Alzheimer's dementia. *Journal of Neuropsychology, 15*(2), 186–203.

5044 Kessels, R. P. C., Montagne, B., Hendriks, A. W., Perrett, D. I., & de Haan, E. H. F. (2014). Assessment of perception of morphed facial expressions using the Emotion Recognition Task: Normative data from healthy participants aged 8–75. *Journal of Neuropsychology, 8*(1), 75–93.
7012 Kestly, T. A. (1991). Emotional processing, seating preference and hemispheric asymmetries. *Dissertation Abstracts International, 51*(9-B), 4624.
6006 Kgolo, T., Grainger, S. A., & Henry, J. D. (2021). Empathy and schizotypy following acquired brain damage. *British Journal of Clinical Psychology, 60*(1), 116–128.
6408 Khorashad, B. S., Baron-Cohen, S., Roshan, G. M., Kazemian, M., Khazai, L., Aghili, Z., Talaei, A., & Afkhamizadeh, M. (2015). The "Reading the Mind in the Eyes" test: Investigation of psychometric properties and test–retest reliability of the Persian version. *Journal of Autism and Developmental Disorders, 45*(9), 2651–2666.
6232 Khorashad, B. S., Khazai, B., Roshan, G. M., Hiradfar, M., Afkhamizadeh, M., & van de Grift, T. C. (2018). Prenatal testosterone and theory of mind development: Findings from disorders of sex development. *Psychoneuroendocrinology, 89,* 250–255.
337 Khosdelazad, S., Jorna, L. S., McDonald, S., Rakers, S. E., Huitema, R. B., Buunk, A. M., & Spikman, J. M. (2020). Comparing static and dynamic emotion recognition tests: Performance of healthy participants. *PLoS ONE, 15*(10), Article e0241297.
837 Khouja, J. N., Attwood, A. S., Penton-Voak, I. S., & Munafò, M. R. (2019). Effects of acute alcohol consumption on emotion recognition in social alcohol drinkers. *Journal of Psychopharmacology, 33*(3), 326–334.
6260 Kidd, D., & Castano, E. (2017). Different stories: How levels of familiarity with literary and genre fiction relate to mentalizing. *Psychology of Aesthetics, Creativity, and the Arts, 11*(4), 474–486.
6155 Kidd, D., & Castano, E. (2019). Reading literary fiction and theory of mind: Three preregistered replications and extensions of Kidd and Castano (2013). *Social Psychological and Personality Science, 10*(4), 522–531.
6830 Kim, S.-M., Kwon, Y.-J., Jung, S.-Y., Kim, M.-J., Cho, Y. S., Kim, H. T., Nam, K.-C., Kim, H., Choi, K.-H., & Choi, J.-S. (2017). Development of the Korean facial emotion stimuli: Korea University Facial Expression Collection 2nd Edition. *Frontiers in Psychology, 8,* Article 769.
104 King, S., Holleran, L., Mothersill, D., Patlola, S., Rokita, K., McManus, R., Kenyon, M., McDonald, C., Hallahan, B., Corvin, A., Morris, D., Kelly, J., McKernan, D., & Donohoe, G. (2021). Early life adversity, functional connectivity and cognitive performance in schizophrenia: The mediating role of IL-6. *Brain, Behavior, and Immunity, 98,* 388–396.
7429 Kirouac, G., Bouchard, M., & St-Pierre, A. (1986). Facial expressions of emotions and ethological behavioral categories. *Perceptual and Motor Skills, 62*(2), 419–423.
6970 Kirouac, G., & Doré, F. Y. (1983). Accuracy and latency of judgment of facial expressions of emotions. *Perceptual and Motor Skills, 57*(3, Pt 1), 683–686.
6968 Kirouac, G., & Doré, F. Y. (1985). Accuracy of the judgment of facial expression of emotions as a function of sex and level of education. *Journal of Nonverbal Behavior, 9*(1), 3–7.
233 Kjærstad, H. L., Eikeseth, F. F., Vinberg, M., Kessing, L. V., & Miskowiak, K. (2021). Neurocognitive heterogeneity in patients with bipolar disorder and their unaffected relatives: Associations with emotional cognition. *Psychological Medicine, 51*(4), 668–679.
169 Klein, H. S., Vanneste, S., & Pinkham, A. E. (2021). The limited effect of neural stimulation on visual attention and social cognition in individuals with schizophrenia. *Neuropsychologia, 157,* Article 107880.
7356 Klein, K. J. K., & Hodges, S. D. (2001). Gender differences, motivation, and empathic accuracy: When it pays to understand. *Personality and Social Psychology Bulletin, 27*(6), 720–730.
366 Kleine Deters, R., Naaijen, J., Rosa, M., Aggensteiner, P. M., Banaschewski, T., Saam, M. C., Schulze, U. M. E., Sethi, A., Craig, M. C., Sagar-Ouriaghli, I., Santosh, P., Castro-Fornieles, J., Penzol, M. J., Arango, C., Werhahn, J. E., Brandeis, D., Franke, B., Glennon, J., Buitelaar, J. K., . . . Dietrich, A. (2020). Executive functioning and emotion recognition in youth with oppositional defiant disorder and/or conduct disorder. *The World Journal of Biological Psychiatry, 21*(7), 539–551.
6429 Knowles, M. L., Lucas, G. M., Baumeister, R. F., & Gardner, W. L. (2015). Choking under social pressure: Social monitoring among the lonely. *Personality and Social Psychology Bulletin, 41*(6), 805–821.
7575 Knowlson, A. (2016). Theory of mind, affective empathy, and academic achievement: A correlative study of children in grades 4 to 6. Master's thesis, Trinity Western University, Langley, British Columbia, Canada.
533 Kohls, G., Baumann, S., Gundlach, M., Scharke, W., Bernhard, A., Martinelli, A., Ackermann, K., Kersten, L., Prätzlich, M., Oldenhof, H., Jansen, L., van den Boogaard, L., Smaragdi, A., Gonzalez-Madruga, K., Cornwell, H., Rogers, J. C., Pauli, R., Clanton, R., Baker, R., . . . Konrad, K. (2020). Investigating sex differences in

emotion recognition, learning, and regulation among youths with conduct disorder. *Journal of the American Academy of Child & Adolescent Psychiatry, 59*(2), 263–273.

1548 Kong, D. T. (2016). Ostracism perception as a multiplicative function of trait self-esteem, mindfulness, and facial emotion recognition ability. *Personality and Individual Differences, 93,* 68–73.

6040 Kopera, M., Zaorska, J., Trucco, E. M., Suszek, H., Kobyliński, P., Zucker, R. A., Nowakowska, M., Wojnar, M., & Jakubczyk, A. (2020). Childhood trauma, alexithymia, and mental states recognition among individuals with alcohol use disorder and healthy controls. *Drug and Alcohol Dependence, 217,* Article 108301.

863 Kosson, D. S., Chi, T., Riser, N. R. E., Walsh, Z., Beussink, C. N., Pera-Guardiola, V., & Briz, A. J. (2019). Facial affect recognition in college students with psychopathic traits: A comparison using tests matched in discriminating power. *Journal of Research in Personality, 78,* 52–60.

6309 Köther, U., Vetorazzi, E., Veckenstedt, R., Hottenrott, B., Bohn, F., Scheu, F., Pfueller, U., Roesch-Ely, D., & Moritz, S. (2017). Bayesian analyses of the effect of metacognitive training on social cognition deficits and overconfidence in errors. *Journal of Experimental Psychopathology, 8*(2), 158–174.

273 Kowallik, A. E., Pohl, M., & Schweinberger, S. R. (2021). Facial imitation improves emotion recognition in adults with different levels of sub-clinical autistic traits. *Journal of Intelligence, 9*(1), Article 4.

5659 Kranefeld, I., Nill, C., & Blickle, G. (2021). Emotion recognition ability for voices, auditory intelligence, general mental ability, and extrinsic career success. *Personality and Individual Differences, 172,* Article 110587.

1202 Kraus, M. W. (2017). Voice-only communication enhances empathic accuracy. *American Psychologist, 72*(7), 644–654.

7307 Kraus, M. W., Côté, S., & Keltner, D. (2010). Social class, contextualism, and empathic accuracy. *Psychological Science, 21*(11), 1716–1723.

7508 Krych-Appelbaum, M., Law, J. B., Jones, D., Barnacz, A., Johnson, A., & Keenan, J. P. (2007). "I think I know what you mean." The role of theory of mind in collaborative communication. *Interaction Studies: Social Behaviour and Communication in Biological and Artificial Systems, 8*(2), 267–280.

5847 Ku, Y.-L., & Lin, M.-T. (2020). Facial emotion recognition and its relationships with neurocognition and theory of mind in schizophrenia. *Chinese Journal of Psychology, 62*(1), 73–98.

2708 Kucharska-Pietura, K., David, A. S., Masiak, M., & Phillips, M. L. (2005). Perception of facial and vocal affect by people with schizophrenia in early and late stages of illness. *The British Journal of Psychiatry, 187*(6), 523–528.

6105 Kuczynski, A. M., Kanter, J. W., & Robinaugh, D. J. (2020). Differential associations between interpersonal variables and quality-of-life in a sample of college students. *Quality of Life Research: An International Journal of Quality of Life Aspects of Treatment, Care & Rehabilitation, 29*(1), 127–139.

1253 Kuhn, L. K., Wydell, T., Lavan, N., McGettigan, C., & Garrido, L. (2017). Similar representations of emotions across faces and voices. *Emotion, 17*(6), 912–937.

5899 Kumar, L., Zhou, A., Sanov, B., Beitler, S., Skrzynski, C. J., & Creswell, K. G. (2022). Indirect effects of theory of mind on alcohol use and problems in underage drinkers: The role of peer pressure to drink. *Addictive Behaviors Reports, 16,* 1–7.

6017 Kynast, J., Polyakova, M., Quinque, E. M., Hinz, A., Villringer, A., & Schroeter, M. L. (2021). Age- and sex-specific standard scores for the Reading the Mind in the Eyes Test. *Frontiers in Aging Neuroscience, 12,* Article 607107.

982 Labayru, G., Arenzana, I., Aliri, J., Zulaica, M., de Munain, A. L., & Sistiaga A., A. (2018). Social cognition in myotonic dystrophy type 1: Specific or secondary impairment? *PLoS ONE, 13*(9), Article e0204227.

6134 Laghi, F., Bianchi, D., Pompili, S., Lonigro, A., & Baiocco, R. (2019). Heavy episodic drinking in late adolescents: The role of theory of mind and conformity drinking motives. *Addictive Behaviors, 96,* 18–25.

6356 Laghi, F., Federico, F., Lonigro, A., Levanto, S., Ferraro, M., Baumgartner, E., & Baiocco, R. (2016). Peer and teacher-selected peer buddies for adolescents with autism spectrum disorders: The role of social, emotional, and mentalizing abilities. *The Journal of Psychology: Interdisciplinary and Applied, 150*(4), 469–484.

6343 Laghi, F., Lonigro, A., Levanto, S., Ferraro, M., Baumgartner, E., & Baiocco, R. (2016). The role of nice and nasty theory of mind in teacher-selected peer models for adolescents with autism spectrum disorders. *Measurement and Evaluation in Counseling and Development, 49*(3), 207–216.

6206 Laghi, F., Lonigro, A., Pallini, S., & Baiocco, R. (2018). Peer buddies in the classroom: The effects on spontaneous conversations in students with autism spectrum disorder. *Child & Youth Care Forum, 47*(4), 517–536.

6031 Laghi, F., Pompili, S., Bianchi, D., Lonigro, A., & Baiocco, R. (2021). Drunkorexia: An examination of the role of theory of mind and emotional awareness among adolescents. *Developmental Neuropsychology, 46*(1), 70–81.

6144 Lai, M.-C., Lombardo, M. V., Chakrabarti, B., Ruigrok, A. N. V., Bullmore, E. T., Suckling, J., Auyeung, B., Happé, F., Szatmari, P., Baron-Cohen, S., & MRC AIMS Consortium. (2019). Neural self-representation in autistic women and association with 'compensatory camouflaging'. *Autism, 23*(5), 1210–1223.
6646 Lai, M.-C., Lombardo, M. V., Pasco, G., Ruigrok, A. N. V., Wheelwright, S. J., Sadek, S. A., Chakrabarti, B., Baron-Cohen, S., & MRC AIMS Consortium. (2011). A behavioral comparison of male and female adults with high functioning autism spectrum conditions. *PLoS ONE, 6*(6), Article e20835.
6537 Lai, M.-C., Lombardo, M. V., Suckling, J., Ruigrok, A. N. V., Chakrabarti, B., Ecker, C., Deoni, S. C. L., Craig, M. C., Murphy, D. G. M., Bullmore, E. T., Baron-Cohen, S., & MRC AIMS Consortium. (2013). Biological sex affects the neurobiology of autism. *Brain: A Journal of Neurology, 136*(9), 2799–2815.
5023 Lambrecht, L., Kreifelts, B., & Wildgruber, D. (2014). Gender differences in emotion recognition: Impact of sensory modality and emotional category. *Cognition and Emotion, 28*(3), 452–469.
259 Lan, X., & Moscardino, U. (2021). Sensitivity to facial emotional expressions and peer relationship problems in Chinese rural-to-urban migrant early adolescents: An exploratory study. *Social Development, 30*(1), 205–224.
687 Lannoy, S., Benzerouk, F., Maurage, P., Barrière, S., Billieux, J., Naassila, M., Kaladjian, A., & Gierski, F. (2019). Disrupted fear and sadness recognition in binge drinking: A combined group and individual analysis. *Alcoholism: Clinical and Experimental Research, 43*(9), 1978–1985.
6942 Larkin, K. T., Martin, R. R., & McClain, S. E. (2002). Cynical hostility and the accuracy of decoding facial expressions of emotions. *Journal of Behavioral Medicine, 25*(3), 285–292.
1437 Laukka, P., Elfenbein, H. A., Thingujam, N. S., Rockstuhl, T., Iraki, F. K., Chui, W., & Althoff, J. (2016). The expression and recognition of emotions in the voice across five nations: A lens model analysis based on acoustic features. *Journal of Personality and Social Psychology, 111*(5), 686–705.
1549 Laurens, K. R., & Cullen, A. E. (2016). Toward earlier identification and preventative intervention in schizophrenia: Evidence from the London Child Health and Development Study. *Social Psychiatry and Psychiatric Epidemiology: The International Journal for Research in Social and Genetic Epidemiology and Mental Health Services, 51*(4), 475–491.
7320 Laurent, S. M., & Hodges, S. D. (2009). Gender roles and empathic accuracy: The role of communion in reading minds. *Sex Roles: A Journal of Research, 60*(5-6), 387–398.
6850 Lavrencic, L. M., Kurylowicz, L., Valenzuela, M. J., Churches, O. F., & Keage, H. A. D. (2016). Social cognition is not associated with cognitive reserve in older adults. *Aging, Neuropsychology, and Cognition, 23*(1), 61–77.
710 Laycock, R., Cutajar, E., & Crewther, S. G. (2019). High schizotypy traits associated with atypical processing of negative emotions with low spatial frequencies. *Schizophrenia Research, 210,* 294–295.
805 Lázaro, E., Amayra, I., López-Paz, J. F., Martínez, O., Pérez, M., Berrocoso, S., García, M., Jometón, A., Al-Rashaida, M., Oliva, M., Parada, P., & Hoffmann, H. (2019). Instrument for assessing the ability to identify emotional facial expressions in healthy children and in children with ADHD: The FEEL Test. *Journal of Attention Disorders, 23*(6), 563–569.
839 Lecomte, T., Théroux, L., Paquin, K., Potvin, S., & Achim, A. (2019). Can social anxiety impact facial emotion recognition in schizophrenia? *Journal of Nervous and Mental Disease, 207*(3), 140–144.
565 Lee, G., Moore, T. M., Basner, M., Nasrini, J., Roalf, D. R., Ruparel, K., Port, A. M., Dinges, D. F., & Gur, R. C. (2020). Age, sex, and repeated measures effects on NASA's "Cognition" Test Battery in STEM educated adults. *Aerospace Medicine and Human Performance, 91*(1), 18–25.
384 Lee, H.-R., Nam, G., & Hur, J.-W. (2020). Development and validation of the Korean version of the Reading the Mind in the Eyes Test. *PLoS ONE, 15*(8), Article e0238309.
688 Lee, J. J., Hardin, A. E., Parmar, B., & Gino, F. (2019). The interpersonal costs of dishonesty: How dishonest behavior reduces individuals' ability to read others' emotions. *Journal of Experimental Psychology: General, 148*(9), 1557–1574.
5980 Lee, S., Jacobsen, E. P., Jia, Y., Snitz, B. E., Chang, C.-C. H., & Ganguli, M. (2021). Reading the mind in the eyes: A population-based study of social cognition in older adults. *The American Journal of Geriatric Psychiatry, 29*(7), 634–642.
7310 Lee, S. A., Guajardo, N. R., Short, S. D., & King, W. (2010). Individual differences in ocular level empathic accuracy ability: The predictive power of fantasy empathy. *Personality and Individual Differences, 49*(1), 68–71.
1755 Lee, S. Y., Bang, M., Kim, K. R., Lee, M. K., Park, J. Y., Song, Y. Y., Kang, J. I., Lee, E., & An, S. K. (2015). Impaired facial emotion recognition in individuals at ultra-high risk for psychosis and with first-episode schizophrenia, and their associations with neurocognitive deficits and self-reported schizotypy. *Schizophrenia Research, 165*(1), 60–65.

6238 Lemmers-Jansen, I. L. J., Krabbendam, L., Amodio, D. M., Van Doesum, N. J., Veltman, D. J., & Van Lange, P. A. M. (2018). Giving others the option of choice: An fMRI study on low-cost cooperation. *Neuropsychologia, 109,* 1–9.

5896 Lenhart, J., & Richter, T. (2022). Does reading a single short story of literary fiction improve social-cognitive skills? Testing the priming hypothesis. *Psychology of Aesthetics, Creativity, and the Arts.* Advance online publication.

7433 Levy, N., & Schlosberg, H. (1960). Woodworth scale values of the Lightfoot pictures of facial expression. *Journal of Experimental Psychology, 60*(2), 121–125.

7287 Lewis, K. L., Hodges, S. D., Laurent, S. M., Srivastava, S., & Biancarosa, G. (2012). Reading between the minds: The use of stereotypes in empathic accuracy. *Psychological Science, 23*(9), 1040–1046.

6810 Lim, K., Yang, Z., Rashid, N. A. A., Tan, B. L., Dauwels, J., & Lee, J. (2021). Comparative study of emotion recognition and theory of mind between major depressive disorder and schizophrenia. *Journal of Affective Disorders, 295,* 1445–1448.

6451 Lindeman, M., Svedholm-Häkkinen, A. M., & Lipsanen, J. (2015). Ontological confusions but not mentalizing abilities predict religious belief, paranormal belief, and belief in supernatural purpose. *Cognition, 134,* 63–76.

6004 Lizcano-Cortés, F., Rasgado-Toledo, J., Giudicessi, A., & Giordano, M. (2021). Theory of mind and its elusive structural substrate. *Frontiers in Human Neuroscience, 15,* Article 618630.

569 López-Morales, H., Zabaletta, V., Vivas, L., & López, M. C. (2020). Reconocimiento de expresiones faciales emocionales. Diferencias en el desarrollo [Developmental differences in the recognition of facial expressions of emotions]. *Psicologia: Teoria e Pesquisa, 36,* Article e3626.

5936 Lord, K. A., Suvak, M. K., & Liverant, G. I. (2022). Social anxiety, rejection sensitivity, and theory of mind decoding ability. *Journal of Clinical Psychology, 78*(4), 656–670.

6069 Łowicki, P., Zajenkowski, M., & Van Cappellen, P. (2020). It's the heart that matters: The relationships among cognitive mentalizing ability, emotional empathy, and religiosity. *Personality and Individual Differences, 161,* Article 109976.

43 Ludyga, S., Gerber, M., Brand, S., Möhring, W., & Pühse, U. (2022). Do different cognitive domains mediate the association between moderate-to-vigorous physical activity and adolescents' off-task behaviour in the classroom? *British Journal of Educational Psychology, 92*(1), 194–211.

1468 Luther, L., Firmin, R. L., Vohs, J. L., Buck, K. D., Rand, K. L., & Lysaker, P. H. (2016). Intrinsic motivation as a mediator between metacognition deficits and impaired functioning in psychosis. *British Journal of Clinical Psychology, 55*(3), 332–347.

1417 Lyusin, D., & Ovsyannikova, V. (2016). Measuring two aspects of emotion recognition ability: Accuracy vs. Sensitivity. *Learning and Individual Differences, 52,* 129–136.

6145 Lyvers, M., Mayer, K., Needham, K., & Thorberg, F. A. (2019). Parental bonding, adult attachment, and theory of mind: A developmental model of alexithymia and alcohol-related risk. *Journal of Clinical Psychology, 75*(7), 1288–1304.

1121 Lyvers, M., McCann, K., Coundouris, S., Edwards, M. S., & Thorberg, F. A. (2018). Alexithymia in relation to alcohol use, emotion recognition, and empathy: The role of externally oriented thinking. *The American Journal of Psychology, 131*(1), 41–51.

6135 Lyvers, M., Scott, K., & Thorberg, F. A. (2019). Social anxiety and alexithymia in relation to problematic drinking and theory of mind. *The American Journal of Psychology, 132*(3), 325–341.

1583 Maat, A., van Haren, N. E. M., Bartholomeusz, C. F., Kahn, R. S., & Cahn, W. (2016). Emotion recognition and theory of mind are related to gray matter Vol. of the prefrontal cortex in schizophrenia. *European Neuropsychopharmacology, 26*(2), 255–264.

7581 MacCann, C., Joseph, D. L., Newman, D. A., & Roberts, R. D. (2014). Emotional intelligence is a second-stratum factor of intelligence: Evidence from hierarchical and bifactor models. *Emotion, 14*(2), 358–374.

5685 MacCann, C., Lievens, F., Libbrecht, N., & Roberts, R. D. (2016). Differences between multimedia and text-based assessments of emotion management: An exploration with the multimedia emotion management assessment (MEMA). *Cognition and Emotion, 30*(7), 1317–1331.

5468 MacCann, C., Pearce, N., & Roberts, R. D. (2011). Emotional intelligence as assessed by situational judgment and emotion recognition tests: Building the nomological net. *Psihologijske Teme, 20*(3), 393–412.

122 MacPherson, H. A., Kudinova, A. Y., Jenkins, G. A., Kim, K. L., Radoeva, P. D., Gilbert, A. C., Barthelemy, C., DeYoung, L., Yen, S., Hower, H., Hunt, J., Keller, M. B., & Dickstein, D. P. (2021). Facial emotion recognition and mood symptom course in young adults with childhood-onset bipolar disorder. *European Archives of Psychiatry and Clinical Neuroscience, 271*(7), 1393–1404.

5918 Maddaluno, O., Aiello, E. N., Roncoroni, C., Prunas, A., & Bolognini, N. (2022). The Reading the Mind in the Eyes Test, Iowa Gambling Task and Interpersonal Reactivity Index: Normative data in an Italian population sample. *Archives of Clinical Neuropsychology, 37*(5), 929–938.
6211 Maillefer, A. V., Udayar, S., & Fiori, M. (2018). Enhancing the prediction of emotionally intelligent behavior: The pat integrated framework involving trait EI, ability EI, and emotion information processing. *Frontiers in Psychology, 9,* Article 1078.
6326 Ma-Kellams, C., & Lerner, J. (2016). Trust your gut or think carefully? Examining whether an intuitive, versus a systematic, mode of thought produces greater empathic accuracy. *Journal of Personality and Social Psychology, 111*(5), 674–685.
897 Mallawaarachchi, S. R., Cotton, S. M., Anderson, J., Killackey, E., & Allott, K. A. (2019). Exploring the use of the Hinting Task in first-episode psychosis. *Cognitive Neuropsychiatry, 24*(1), 65–79.
5747 Mandal, M. K. (2008). Cultural in-group advantage in accuracy at recognizing vocal expressions of emotion. *Psychological Studies, 53*(2), 126–132.
124 Manierka, M. S., Rezaei, R., Palacios, S., Haigh, S. M., & Hutsler, J. J. (2021). In the mood to be social: Affective state influences facial emotion recognition in healthy adults. *Emotion, 21*(7), 1576–1581.
6714 Mar, R. A., Oatley, K., Hirsh, J., dela Paz, J., & Peterson, J. B. (2006). Bookworms versus nerds: Exposure to fiction versus non-fiction, divergent associations with social ability, and the simulation of fictional social worlds. *Journal of Research in Personality, 40*(5), 694–712.
6832 Martin, A. K., Ceslis, A., & Robinson, G. A. (2022). Social inference from middle to older adulthood. *Archives of Clinical Neuropsychology, 37*(8), 1653–1661.
5239 Martínez-Sánchez, F., Fernández-Abascal, E. G., & Martínez-Modia, J. C. (2013). Adaptación española de la versión reducida multicanal del Perfil de Sensibilidad No Verbal (MiniPONS) [The Spanish version of the short multichannel version of the Profile of Nonverbal Sensitivity (MiniPONS)]. *Anales de Psicología, 29*(2), 604–613.
1394 Martinez-Sanchez, F., Fernández-Abascal, E. G., & Sánchez-Pérez, N. (2017). Recognition of emotional facial expressions in alexithymia. *Studia Psychologica, 59*(3), 206–216.
750 Martins, A. T., Ros, A., Valério, L., & Faísca, L. (2019). Basic emotion recognition according to clinical personality traits. *Current Psychology: A Journal for Diverse Perspectives on Diverse Psychological Issues, 38*(3), 879–889.
234 Mattavelli, G., Barvas, E., Longo, C., Zappini, F., Ottaviani, D., Malaguti, M. C., Pellegrini, M., & Papagno, C. (2021). Facial expressions recognition and discrimination in Parkinson's disease. *Journal of Neuropsychology, 15*(1), 46–68.
272 Maurage, P., Pabst, A., Lannoy, S., D'Hondt, F., de Timary, P., Gaudelus, B., & Peyroux, E. (2021). Tackling heterogeneity: Individual variability of emotion decoding deficits in severe alcohol use disorder. *Journal of Affective Disorders, 279,* 299–307.
328 Mayukha, A., Andrade, I., & Cone, J. (2020). Opposing contributions of psychologically distinct components of empathy to empathic accuracy. *Journal of Experimental Psychology: General, 149*(11), 2169–2186.
7111 McClure, E. B., & Nowicki, S., Jr. (2001). Associations between social anxiety and nonverbal processing skill in preadolescent boys and girls. *Journal of Nonverbal Behavior, 25*(1), 3–19.
5480 McCubbin, J. A., Merritt, M. M., Sollers, J. J. III, Evans, M. K., Zonderman, A. B., Lane, R. D., & Thayer, J. F. (2011). Cardiovascular-emotional dampening: The relationship between blood pressure and recognition of emotion. *Psychosomatic Medicine, 73*(9), 743–750.
6851 McDonald, S., Fisher, A., Togher, L., Tate, R., Rushby, J., English, T., Kelly, M., Mathersul, D., Froreich, F., & Francis, H. (2015). Adolescent performance on The Awareness of Social Inference Test: TASIT. *Brain Impairment, 16*(1), 3–18.
6842 McDonald, S., Honan, C., Allen, S. K., El-Helou, R., Kelly, M., Kumfor, F., Piguet, O., Hazelton, J. L., Padgett, C., & Keage, H. A. D. (2018). Normal adult and adolescent performance on TASIT-S, a short version of The Assessment of Social Inference Test. *The Clinical Neuropsychologist, 32*(4), 700–719.
7509 McGlade, N., Behan, C., Hayden, J., O'Donoghue, T., Peel, R., Haq, F., Gill, M., Corvin, A., O'Callaghan, E., & Donohoe, G. Mental state decoding v. mental state reasoning as a mediator between cognitive and social function in psychosis. *British Journal of Psychiatry, 193*, 77-78.
7139 McKinney, J. A. (1986). The training and generalization of nonverbal decoding ability. *Dissertation Abstracts International, 47*(5-B), 2175.
737 McKown, C. (2019). Reliability, factor structure, and measurement invariance of a web-based assessment of children's social-emotional comprehension. *Journal of Psychoeducational Assessment, 37*(4), 435–449.

5702 Meadors, J. D. (2015). Rethinking empathic accuracy. *Dissertation Abstracts International: Section B: The Sciences and Engineering, 76*(3-B(E)).

527 Mehl, S., Hesse, K., Schmidt, A.-C., Landsberg, M. W., Soll, D., Bechdolf, A., Herrlich, J., Kircher, T., Klingberg, S., Müller, B. W., Wiedemann, G., Wittorf, A., Wölwer, W., & Wagner, M. (2020). Theory of mind, emotion recognition, delusions and the quality of the therapeutic relationship in patients with psychosis—A secondary analysis of a randomized-controlled therapy trial. *BMC Psychiatry, 20,* Article 59.

6082 Meinhardt-Injac, B., Daum, M. M., & Meinhardt, G. (2020). Theory of mind development from adolescence to adulthood: Testing the two-component model. *British Journal of Developmental Psychology, 38*(2), 289–303.

572 Meinhardt-Injac, B., Kurbel, D., & Meinhardt, G. (2020). The coupling between face and emotion recognition from early adolescence to young adulthood. *Cognitive Development, 53,* Article 100851.

310 Meléndez, J. C., Satorres, E., Reyes-Olmedo, M., Delhom, I., Real, E., & Lora, Y. (2020). Emotion recognition changes in a confinement situation due to COVID-19. *Journal of Environmental Psychology, 72,* Article 101518.

154 Melkonian, A., Ham, L., & Mobley, A. (2021). Gender moderates the association between acute alcohol intoxication and facial emotion recognition in a naturalistic field study setting. *Alcoholism: Clinical and Experimental Research, 45*(8), 1684–1692.

1232 Melkonian, A. J., Ham, L. S., Bridges, A. J., & Fugitt, J. L. (2017). Facial emotion identification and sexual assault risk detection among college student sexual assault victims and nonvictims. *Journal of American College Health, 65*(7), 466–473.

31 Menting-Henry, S., Hidalgo-Lopez, E., Aichhorn, M., Kronbichler, M., Kerschbaum, H., & Pletzer, B. (2022). Oral contraceptives modulate the relationship between resting brain activity, amygdala connectivity and emotion recognition—A resting state fMRI study. *Frontiers in Behavioral Neuroscience, 16,* Article 775796.

2709 Merten, J. (2005). Culture, gender and the recognition of the basic emotions. *Psychologia: An International Journal of Psychology in the Orient, 48*(4), 306–316.

5956 Meyebovsky, M. M., Tabullo, Á. J., & García, C. S. (2021). Associations between theory of mind and emotion regulation in Argentinean adults. *Current Psychology: A Journal for Diverse Perspectives on Diverse Psychological Issues, 40*(12), 6118–6131.

6717 Meyer, J., & Shean, G. (2006). Social-cognitive functioning and schizotypal characteristics. *The Journal of Psychology: Interdisciplinary and Applied, 140*(3), 199–207.

7510 Meyer, J. K. (2009). In the eye of the beholder: attachment style differences in emotion perception. *The Penn State McNair Journal, 16*, 74-87.

6121 Michaelian, J. C., Mowszowski, L., Guastella, A. J., Henry, J. D., Duffy, S., McCade, D., & Naismith, S. L. (2019). Theory of mind in mild cognitive impairment—Relationship with limbic structures and behavioural change. *Journal of the International Neuropsychological Society, 25*(10), 1023–1034.

1028 Misailidi, P. (2018). Individual differences in children's understanding of guilt: Links with theory of mind. *The Journal of Genetic Psychology: Research and Theory on Human Development, 179*(4), 219–229.

534 Modinos, G., Kempton, M. J., Tognin, S., Calem, M., Porffy, L., Antoniades, M., Mason, A., Azis, M., Allen, P., Nelson, B., McGorry, P., Pantelis, C., Riecher-Rössler, A., Borgwardt, S., Bressan, R., Barrantes-Vidal, N., Krebs, M.-O., Nordentoft, M., Glenthøj, B., . . . EU-GEI High Risk Study Group. (2020). Association of adverse outcomes with emotion processing and its neural substrate in individuals at clinical high risk for psychosis. *JAMA Psychiatry, 77*(2), 190–200.

1844 Molinero, C., Bonete, S., Gómez-Pérez, M. M., & Calero, M. D. (2015). Estudio normativo del "test de 60 caras de Ekman" para adolescentes españoles [A normative study of the Ekman 60-faces test in Spanish adolescents]. *Behavioral Psychology / Psicología Conductual: Revista Internacional Clínica y de la Salud, 23*(2), 361–371.

5703 Momm, T., Blickle, G., Liu, Y., Wihler, A., Kholin, M., & Menges, J. I. (2015). It pays to have an eye for emotions: Emotion recognition ability indirectly predicts annual income. *Journal of Organizational Behavior, 36*(1), 147–163.

15 Monroy, M., Cowen, A. S., & Keltner, D. (2022). Intersectionality in emotion signaling and recognition: The influence of gender, ethnicity, and social class. *Emotion, 22*(8), 1980-1988.

7463 Montagne, B., Kessels, R. P. C., Frigerio, E., de Haan, E. H. F., & Perrett, D. I. (2005). Sex differences in the perception of affective facial expressions: Do men really lack emotional sensitivity? *Cognitive Processing, 6*(2), 136–141.

7679 Moore, T. M., Gur, R. C., Thomas, M. L., Brown, G. G., Nock, M. K., Savitt, A. P., Keilp, J. G., Heeringa, S., Ursano, R. J., Stein, M. B., & Army STARRS Collaborators. (2019). Development, administration, and structural

validity of a brief, computerized neurocognitive battery: Results from the Army Study to Assess Risk and Resilience in Servicemembers. *Assessment, 26*(1), 125–143.
7565 Morand, D. A. (2001). The emotional intelligence of managers: Assessing the construct validity of a nonverbal measure of "people skills." *Journal of Business and Psychology, 16*(1), 21–33.
7465 Morgado, I. A., Cangemi, J. P., Miller, R., & O'Connor, J. (1993). Accuracy of decoding facial expressions in those engaged in people oriented activities vs. those engaged in non-people oriented activities. *Studia Psychologica, 35*(1), 73–80.
125 Morningstar, M., Gilbert, A. C., Burdo, J., Leis, M., & Dirks, M. A. (2021). Recognition of vocal socioemotional expressions at varying levels of emotional intensity. *Emotion, 21*(7), 1570–1575.
5923 Moseley, R. L., Liu, C. H., Gregory, N. J., Smith, P., Baron-Cohen, S., & Sui, J. (2022). Levels of self-representation and their sociocognitive correlates in late-diagnosed autistic adults. *Journal of Autism and Developmental Disorders, 52*(7), 3246–3259.
1150 Mossaheb, N., Kaufmann, R. M., Schlögelhofer, M., Aninilkumparambil, T., Himmelbauer, C., Gold, A., Zehetmayer, S., Hoffmann, H., Traue, H. C., & Aschauer, H. (2018). The impact of sex differences on odor identification and facial affect recognition in patients with schizophrenia spectrum disorders. *Frontiers in Psychiatry, 9,* Article 9.
6172 Mouw, J. M., Saab, N., Pat-El, R. J., & van den Broek, P. (2019). Student- and task-related predictors of primary-school students' perceptions of cooperative learning activities. *Pedagogische Studiën, 96*(2), 98–122.
1646 Mualem, O., & Lavidor, M. (2015). Music education intervention improves vocal emotion recognition. *International Journal of Music Education, 33*(4), 413–425.
1776 Mueller, D. R., Schmidt, S. J., & Roder, V. (2015). One-year randomized controlled trial and follow-up of integrated neurocognitive therapy for schizophrenia outpatients. *Schizophrenia Bulletin, 41*(3), 604–616.
7466 Mufson, L., & Nowicki, S. (1991). Factors affecting the accuracy of facial affect recognition. *The Journal of Social Psychology, 131*(6), 815–822.
6421 Muhtadie, L., Koslov, K., Akinola, M., & Mendes, W. B. (2015). Vagal flexibility: A physiological predictor of social sensitivity. *Journal of Personality and Social Psychology, 109*(1), 106–120.
6516 Müller, C. M., & Gmünder, L. (2014). An evaluation of the "Reading the Mind in the Eyes-Test" with seventh to ninth graders. *Journal of Mental Health Research in Intellectual Disabilities, 7*(1), 34–44.
2731 Mullins, D. T. (2005). Social and emotional correlates of nonverbal processing accuracy and response time in college students. *Dissertation Abstracts International: Section B: The Sciences and Engineering, 65*(8-B), 4297.
5754 Mullins-Nelson, J. L., Salekin, R. T., & Leistico, A.-M. R. (2006). Psychopathy, empathy, and perspective-taking ability in a community sample: Implications for the successful psychopathy concept. *The International Journal of Forensic Mental Health, 5*(2), 133–149.
7122 Mumley, D. L. (1996). Gender differences in depressive realism: The role of nonverbal decoding ability and relational self-concept. *Dissertation Abstracts International: Section B: The Sciences and Engineering, 57*(4-B), 2876.
5620 Murry, M. W. E., & Isaacowitz, D. M. (2018). Age similarities in interpersonal perception and conversation ability. *Journal of Nonverbal Behavior, 42*(1), 101–111.
214 Nahal, P., Hurd, P. L., Read, S., & Crespi, B. (2021). Cognitive empathy as imagination: Evidence from reading the mind in the eyes in autism and schizotypy. *Frontiers in Psychiatry, 12,* Article 665721.
6213 Nakajima, R., Yordanova, Y. N., Duffau, H., & Herbet, G. (2018). Neuropsychological evidence for the crucial role of the right arcuate fasciculus in the face-based mentalizing network: A disconnection analysis. *Neuropsychologia, 115,* 179–187.
1533 Narambuena, L., Vaiman, M., & Pereno, G. L. (2016). Reconocimiento de emociones faciales en adultos mayores de la ciudad de Córdoba [Facial emotion recognition in older adults from the city of Córdoba]. *Psykhe: Revista de la Escuela de Psicología, 25*(1), 1–13.
1009 Navarra-Ventura, G., Fernandez-Gonzalo, S., Turon, M., Pousa, E., Palao, D., Cardoner, N., & Jodar, M. (2018). Gender differences in social cognition: A cross-sectional pilot study of recently diagnosed patients with schizophrenia and healthy subjects. *The Canadian Journal of Psychiatry / La Revue canadienne de psychiatrie, 63*(8), 538–546.
6009 Navarro, E., Goring, S. A., & Conway, A. R. A. (2021). The relationship between theory of mind and intelligence: A formative g approach. *Journal of Intelligence, 9*(1), Article 11.
7511 Nettle, D., & Liddle, B. (2008). Agreeableness is related to social-cognitive, but not social-perceptual, theory of mind. *European Journal of Personality, 22*(4), 323–335.
7156 Nitschke, J. P., Pruessner, J. C., & Bartz, J. A. (2022). Stress and stress-induced glucocorticoids facilitate empathic accuracy in men but have no effects for women. *Psychological Science, 33*(10), 1783–1794.

6380 Noble, A. J., Robinson, A., & Marson, A. G. (2016). Are "Theory of Mind" skills in people with epilepsy related to how stigmatised they feel? An exploratory study. *Behavioural Neurology, 2016,* Article 5025174.
9002 Nowicki, S., Jr. (1998). Processing of emotional cues among emotionally disturbed high school students. Unpublished data. (from McClure 2000 meta-analysis)
9003 Nowicki, S., Jr., & Halpern, A. (1997). Mothers' ability to recognize emotional expressions and their children's recognition ability and locus of control. Unpublished data. (from McClure 2000 meta)
7468 Nowicki, S., & Hartigan, M. (1988). Accuracy of facial affect recognition as a function of locus of control orientation and anticipated interpersonal interaction. *The Journal of Social Psychology, 128*(3), 363–372.
7469 Nowicki, S., & Richman, D. (1985). The effect of standard, motivation, and strategy instructions on the facial processing accuracy of internal and external subjects. *Journal of Research in Personality, 19*(4), 354–364.
9000 Ogawa, K., & Hall, J. A. (2017). Unpublished data, Northeastern University.
781 Olderbak, S., Wilhelm, O., Hildebrandt, A., & Quoidbach, J. (2019). Sex differences in facial emotion perception ability across the lifespan. *Cognition and Emotion, 33*(3), 579–588.
6401 Olderbak, S., Wilhelm, O., Olaru, G., Geiger, M., Brenneman, M. W., & Roberts, R. D. (2015). A psychometric analysis of the Reading the Mind in the Eyes Test: Toward a brief form for research and applied settings. *Frontiers in Psychology, 6*, 1503.
782 Oliver, L. D., Haltigan, J. D., Gold, J. M., Foussias, G., DeRosse, P., Buchanan, R. W., Malhotra, A. K., Voineskos, A. N., & SPINS Group. (2019). Lower- and higher-level social cognitive factors across individuals with schizophrenia spectrum disorders and healthy controls: Relationship with neurocognition and functional outcome. *Schizophrenia Bulletin, 45*(3), 629–638.
6313 Orme, W. H. (2017). An exploration of humility as a facilitator of mentalization during attachment system activation. *Dissertation Abstracts International: Section B: The Sciences and Engineering, 78*(6-B(E)).
7628 Ortuño-Sierra, J., Aritio-Solana, R., del Casal, A. D. G., & Fonseca-Pedrero, E. (2021). Neurocognitive functioning in adolescents at risk for suicidal behaviors. *Archives of Suicide Research, 25*(3), 657–671.
6173 Ospina, L. H., Shanahan, M., Perez-Rodriguez, M. M., Chan, C. C., Clari, R., & Burdick, K. E. (2019). Alexithymia predicts poorer social and everyday functioning in schizophrenia and bipolar disorder. *Psychiatry Research, 273,* 218–226.
8004 Osiurak, F., Cretel, C., Duhau-Marmon, N., Fournier, I., Marignier, L., De Oliveira, E., Navarro, J., & Reynaud, E. (2020). The pedagogue, the engineer, and the friend: From whom do we learn? *Human Nature, 31*(4), 462–482.
6106 Osiurak, F., De Oliveira, E., Navarro, J., & Reynaud, E. (2020). The castaway island: Distinct roles of theory of mind and technical reasoning in cumulative technological culture. *Journal of Experimental Psychology: General, 149*(1), 58–66.
1328 Otsuka, S., Uono, S., Yoshimura, S., Zhao, S., & Toichi, M. (2017). Emotion perception mediates the predictive relationship between verbal ability and functional outcome in high-functioning adults with autism spectrum disorder. *Journal of Autism and Developmental Disorders, 47*(4), 1166–1182.
6055 Pagni, B. A., Walsh, M. J. M., Rogers, C., & Braden, B. B. (2020). Social cognition in autism spectrum disorder across the adult lifespan: Influence of age and sex on Reading the Mind in the Eyes task in a cross-sectional sample. *Frontiers in Integrative Neuroscience, 14,* Article 571408.
447 Pahnke, R., Mau-Moeller, A., Hamm, A. O., & Lischke, A. (2020). Reading the Mind in the Eyes of Children Test (RME-C-T): Development and validation of a complex emotion recognition test. *Frontiers in Psychiatry, 11,* Article 376.
1124 Pajevic, M., Vukosavljevic-Gvozden, T., Stevanovic, N., & Neumann, C. S. (2018). The relationship between the dark tetrad and a two-dimensional view of empathy. *Personality and Individual Differences, 123,* 125–130.
1096 Palermo, R., Jeffery, L., Lewandowsky, J., Fiorentini, C., Irons, J. L., Dawel, A., Burton, N., McKone, E., & Rhodes, G. (2018). Adaptive face coding contributes to individual differences in facial expression recognition independently of affective factors. *Journal of Experimental Psychology: Human Perception and Performance, 44*(4), 503–517.
7583 Palermo, R., O'Connor, K. B., Davis, J. M., Irons, J., & McKone, E. (2013). New tests to measure individual differences in matching and labelling facial expressions of emotion, and their association with ability to recognise vocal emotions and facial identity. *PloS ONE, 8*(6), e68126.
5919 Palumbo, F., Iazzolino, B., Peotta, L., Canosa, A., Manera, U., Grassano, M., Casale, F., Pellegrino, G., Rizzone, M. G., Vasta, R., Moglia, C., Chiò, A., & Calvo, A. (2022). Social cognition deficits in amyotrophic lateral sclerosis: A pilot cross-sectional population-based study. *European Journal of Neurology, 29*(8), 2211–2219.

6328 Panero, M. E., Weisberg, D. S., Black, J., Goldstein, T. R., Barnes, J. L., Brownell, H., & Winner, E. (2016). Does reading a single passage of literary fiction really improve theory of mind? An attempt at replication. *Journal of Personality and Social Psychology, 111*(5), e46–e54.
1457 Papini, C., White, T. P., Montagna, A., Brittain, P. J., Froudist-Walsh, S., Kroll, J., Karolis, V., Simonelli, A., Williams, S. C., Murray, R. M., & Nosarti, C. (2016). Altered resting-state functional connectivity in emotion-processing brain regions in adults who were born very preterm. *Psychological Medicine, 46*(14), 3025–3039.
72 Parada-Fernández, P., Herrero-Fernández, D., Jorge, R., & Comesaña, P. (2022). Wearing mask hinders emotion recognition, but enhances perception of attractiveness. *Personality and Individual Differences, 184,* Article 111195.
1206 Park, S., Kim, T., Shin, S. A, Kim, Y. K., Sohn, B. K., Park, H.-J., Youn, J.-H., & Lee, J.-Y. (2017). Behavioral and neuroimaging evidence for facial emotion recognition in elderly Korean adults with mild cognitive impairment, Alzheimer’s disease, and frontotemporal dementia. *Frontiers in Aging Neuroscience, 9,* Article 389.
236 Parsons, C. E., Nummenmaa, L., Sinerva, E., Korja, R., Kajanoja, J., Young, K. S., Karlsson, H., & Karlsson, L. (2021). Investigating the effects of perinatal status and gender on adults’ responses to infant and adult facial emotion. *Emotion, 21*(2), 337–349.
958 Passarelli, M., Masini, M., Bracco, F., Petrosino, M., & Chiorri, C. (2018). Development and validation of the Facial Expression Recognition Test (FERT). *Psychological Assessment, 30*(11), 1479–1490.
1441 Paulmann, S., Furnes, D., Bøkenes, A. M., & Cozzolino, P. J. (2016). How psychological stress affects emotional prosody. *PLoS ONE, 11*(11), Article e0165022.
73 Pearce, E., Barreto, M., Victor, C., Hammond, C., Eccles, A. M., Richins, M. T., O'Neil, A., Knowles, M. L., & Qualter, P. (2022). Choking under pressure: Does it get easier with age? How loneliness affects social monitoring across the life span. *International Journal of Behavioral Development, 46*(1), 50–62.
6151 Pearce, E., Wlodarski, R., Machin, A., & Dunbar, R. I. M. (2019). Exploring the links between dispositions, romantic relationships, support networks and community inclusion in men and women. *PLoS ONE, 14*(5), Article e0216210.
6061 Penner, F., McLaren, V., Leavitt, J., Akca, O. F., & Sharp, C. (2020). Implicit and explicit mentalizing deficits in adolescent inpatients: Specificity and incremental value of borderline pathology. *Journal of Personality Disorders, 34*(Suppl B), 64–83.
6201 Pestana, J., Menéres, S., Gouveia, M. J., & Oliveira, R. F. (2018). The Reading the Mind in the Eyes Test: A Portuguese version of the adults’ test. *Análise Psicológica, 36*(3), 369–381.
6034 Peters, M. (2021). Trauma and mentalization ability in older patients: An empirical contribution to the effect of trauma in old age. GeroPsych: *Journal of Gerontopsychology and Geriatric Psychiatry, 34*(4), 189-199.
5998 Petrocchi, S., Filipponi, C., Antonietti, C., Levante, A., & Lecciso, F. (2021). Theory of mind as a mediator between emotional trust beliefs and interpersonal communication competence in a group of young adults. *Psychological Reports, 124*(2), 555–576.
7522 Pfaff, P. L. (1954). An experimental study of the communication of feeling without contextual material. *Speech Monographs, 21*, 155-156.
5895 Pieslinger, J. F., Wiskerke, J., & Igelström, K. (2022). Contributions of face processing, social anhedonia and mentalizing to the expression of social autistic-like traits. *Frontiers in Behavioral Neuroscience, 16,* Article 1046097.
1566 Pietschnig, J., Aigner-Wöber, R., Reischenböck, N., Kryspin-Exner, I., Moser, D., Klug, S., Auff, E., Dal-Bianco, P., Pusswald, G., & Lehrner, J. (2016). Facial emotion recognition in patients with subjective cognitive decline and mild cognitive impairment. *International Psychogeriatrics, 28*(3), 477–485.
1513 Pietschnig, J., Schröder, L., Ratheiser, I., Kryspin-Exner, I., Pflüger, M., Moser, D., Auff, E., Pirker, W., Pusswald, G., & Lehrner, J. (2016). Facial emotion recognition and its relationship to cognition and depressive symptoms in patients with Parkinson's disease. *International Psychogeriatrics, 28*(7), 1165–1179.
6880 Pistoia, F., Conson, M., Carolei, A., Dema, M. G., Splendiani, A., Curcio, G., & Sacco, S. (2018). Post-earthquake distress and development of emotional expertise in young adults. *Frontiers in Behavioral Neuroscience, 12,* Article 91.
5763 Pitterman, H., & Nowicki, S., Jr. (2004). A Test of the Ability to Identify Emotion in Human Standing and Sitting Postures: The Diagnostic Analysis of Nonverbal Accuracy-2 Posture Test (DANVA2-POS). *Genetic, Social, and General Psychology Monographs, 130*(2), 146–162.
6007 Pluck, G., Córdova, M. A., Bock, C., Chalen, I., & Trueba, A. F. (2021). Socio-economic status, executive functions, and theory of mind ability in adolescents: Relationships with language ability and cortisol. *British Journal of Developmental Psychology, 39*(1), 19–38.

266 Poncet, F., Soussignan, R., Jaffiol, M., Gaudelus, B., Leleu, A., Demily, C., Franck, N., & Baudouin, J.-Y. (2021). The spatial distribution of eye movements predicts the (false) recognition of emotional facial expressions. *PLoS ONE, 16*(1), Article e0245777.

1208 Pozzoli, T., Gini, G., & Altoè, G. (2017). Associations between facial emotion recognition and young adolescents' behaviors in bullying. *PLoS ONE, 12*(11), Article e0188062.

919 Prada, M., Garrido, M. V., Camilo, C., & Rodrigues, D. L. (2018). Subjective ratings and emotional recognition of children's facial expressions from the CAFE set. *PLoS ONE, 13*(12), Article e0209644.

1988 Prado, C., Mellor, D., Byrne, L. K., Wilson, C., Xu, X., & Liu, H. (2014). Facial emotion recognition: A cross-cultural comparison of Chinese, Chinese living in Australia, and Anglo-Australians. *Motivation and Emotion, 38*(3), 420–428.

6288 Preti, A., Vellante, M., & Petretto, D. R. (2017). The psychometric properties of the "Reading the Mind in the Eyes" Test: An item response theory (IRT) analysis. *Cognitive Neuropsychiatry, 22*(3), 233–253.

7576 Puskar, A. (2011). Investigating the relationship between the cognitive and affective components of empathy and frontal lobe functioning in college students. Doctoral dissertation, Indiana University of Pennsylvania, Indiana, PA.

158 Puthillam, A., Karandikar, S., & Kapoor, H. (2021). I see how you feel: How the Dark Triad recognizes emotions. *Current Psychology: A Journal for Diverse Perspectives on Diverse Psychological Issues, 40*(8), 3966–3973.

6295 Quednow, B. B., Hulka, L. M., Preller, K. H., Baumgartner, M. R., Eisenegger, C., & Vonmoos, M. (2017). Stable self-serving personality traits in recreational and dependent cocaine users. *PLoS ONE, 12*(3), Article e0172853.

8006 Quesque, F., Coutrot, A., Cox, S., de Souza, L. C., Baez, S., Cardona, J. F., Mulet-Perreault, H., Flanagan, E., Neely-Prado, A., Clarens, M. F., Cassimiro, L., Musa, G., Kemp, J., Botzung, A., Philippi, N., Cosseddu, M., Trujillo-Llano, C., Grisales-Cardenas, J. S., Fittipaldi, S., . . . Bertoux, M. (2022). Does culture shape our understanding of others' thoughts and emotions? An investigation across 12 countries. *Neuropsychology, 36*(7), 664–682.

486 Quidé, Y., Wilhelmi, C., & Green, M. J. (2020). Structural brain morphometry associated with theory of mind in bipolar disorder and schizophrenia. *PsyCh Journal, 9*(2), 234–246.

7178 Quinn, S. K. (2021). Down, but not out: Rejection impairs social monitoring, but enhances performance on nonsocial tasks. *Dissertation Abstracts International: Section B: The Sciences and Engineering, 82*(9-B).

5351 Quintana, D. S., Guastella, A. J., Outhred, T., Hickie, I. B., & Kemp, A. H. (2012). Heart rate variability is associated with emotion recognition: Direct evidence for a relationship between the autonomic nervous system and social cognition. *International Journal of Psychophysiology, 86*(2), 168–172.

6879 Quintero, L. A. M., Muñoz-Delgado, J., Sánchez-Ferrer, J. C., Fresán, A., Brüne, M., & Montis, I. A. d. (2018). Facial emotion recognition and empathy in employees at a juvenile detention center. *International Journal of Offender Therapy and Comparative Criminology, 62*(8), 2430–2446.

26 Rabin, R. A., Parvaz, M. A., Alia-Klein, N., & Goldstein, R. Z. (2022). Emotion recognition in individuals with cocaine use disorder: The role of abstinence length and the social brain network. *Psychopharmacology, 239*(4), 1019–1033.

140 Radlak, B., Cooper, C., Summers, F., & Phillips, L. H. (2021). Multiple sclerosis, emotion perception and social functioning. *Journal of Neuropsychology, 15*(3), 500–515.

6641 Ragsdale, G., & Foley, R. A. (2011). A maternal influence on reading the mind in the eyes mediated by executive function: Differential parental influences on full and half-siblings. *PLoS ONE, 6*(8), Article e23236.

7475 Rahman, Q., Wilson, G. D., & Abrahams, S. (2004). Sex, sexual orientation, and identification of positive and negative facial affect. *Brain and Cognition, 54*(3), 179–185.

5943 Raine, A., Chen, F. R., & Waller, R. (2022). The Cognitive, Affective and Somatic Empathy Scales for adults. *Personality and Individual Differences, 185,* Article 111238.

5999 Rajab, A., Shafizadeh, M., Nakhjavani, M., Vahabie, A.-H., Salehi, M., Zarei, S., Memari, A., & Mirfazeli, F. S. (2021). Digit ratio (2D:4D) a possible biomarker for cognitive style: A study on Iranian engineering and mathematics university students. *Personality and Individual Differences, 172,* Article 110575.

930 Rappaport, L. M., Carney, D. M., Verhulst, B., Neale, M. C., Blair, J., Brotman, M. A., Pine, D. S., Leibenluft, E., Hettema, J. M., & Roberson-Nay, R. (2018). A developmental twin study of emotion recognition and its negative affective clinical correlates. *Journal of the American Academy of Child & Adolescent Psychiatry, 57*(12), 925–933.

6214 Redondo, I., & Herrero-Fernández, D. (2018). Validation of the Reading the Mind in the Eyes Test in a healthy Spanish sample and women with anorexia nervosa. *Cognitive Neuropsychiatry, 23*(4), 201–217.

5368 Regenbogen, C., Schneider, D. A., Finkelmeyer, A., Kohn, N., Derntl, B., Kellermann, T., Gur, R. E., Schneider, F., & Habel, U. (2012). The differential contribution of facial expressions, prosody, and speech content to empathy. *Cognition and Emotion, 26*(6), 995–1014.

9004 Reutemann, M. (2019). Unpublished data, University of Applied Sciences for Police and Public Administration in North Rhine-Westphalia (Germany).

2030 Ribeiro, L. A., & Fearon, P. (2010). Theory of mind and attentional bias to facial emotional expressions: A preliminary study. *Scandinavian Journal of Psychology, 51*(4), 285–289.

6388 Ridinger, G., & McBride, M. (2015). Money affects theory of mind differently by gender. *PLoS ONE, 10*(12), Article e0143973.

7566 Riggio, R. E., Tucker, J., & Coffaro, D. (1989). Social skills and empathy. *Personality and Individual Differences, 10*(1), 93–99.

6194 Rnic, K., Sabbagh, M. A., Washburn, D., Bagby, R. M., Ravindran, A., Kennedy, J. L., Strauss, J., & Harkness, K. L. (2018). Childhood emotional abuse, physical abuse, and neglect are associated with theory of mind decoding accuracy in young adults with depression. *Psychiatry Research, 268,* 501–507.

7770 Roalf, D. R., Gur, R. E., Ruparel, K., Calkins, M. E., Satterthwaite, T. D., Bilker, W. B., Hakonarson, H., Harris, L. J., & Gur, R. C. (2014). Within-individual variability in neurocognitive performance: Age- and sex-related differences in children and youths from ages 8 to 21. *Neuropsychology, 28*(4), 506–518.

1683 Robinson, L. J., Gray, J. M., Burt, M., Ferrier, I. N., & Gallagher, P. (2015). Processing of facial emotion in bipolar depression and euthymia. *Journal of the International Neuropsychological Society, 21*(9), 709–721.

6846 Rocca, P., Galderisi, S., Rossi, A., Bertolino, A., Rucci, P., Gibertoni, D., Montemagni, C., Sigaudo, M., Mucci, A., Bucci, P., Acciavatti, T., Aguglia, E., Amore, M., Bellomo, A., De Ronchi, D., Dell'Osso, L., Di Fabio, F., Girardi, P., Goracci, A., . . . Italian Network for Research on Psychoses. (2016). Social cognition in people with schizophrenia: A cluster-analytic approach. *Psychological Medicine, 46*(13), 2717–2729.

5602 Rojahn, J., Kroeger, T. L., & McElwain, D. C. (1995). Psychometric properties and preliminary norms of the Penn Facial Discrimination Task in adults with mental retardation. *Journal of Developmental and Physical Disabilities, 7*(4), 285–301.

6359 Rosen, J. B., Brand, M., & Kalbe, E. (2016). Empathy mediates the effects of age and sex on altruistic moral decision making. *Frontiers in Behavioral Neuroscience, 10,* Article 67.

8002 Rosenthal, R., & DePaulo, B. M. (1979). Sex differences in eavesdropping on nonverbal cues. *Journal of Personality and Social Psychology, 37*(2), 273–285.

5611 Rosenthal, R., Hall, J. A., DiMatteo, M. R., Rogers, P. L., & Archer, D. (1979). *Sensitivity to nonverbal communication: The PONS test*. Johns Hopkins University Press.

6116 Rosi, A., Nola, M., Lecce, S., & Cavallini, E. (2019). Prosocial behavior in aging: Which factors can explain age-related differences in social-economic decision making? *International Psychogeriatrics, 31*(12), 1747–1757.

5655 Rosip, J. C., & Hall, J. A. (2004). Knowledge of nonverbal cues, gender, and nonverbal decoding accuracy. *Journal of Nonverbal Behavior, 28*(4), 267–286.

7146 Ross, M. S. (1983). The effects of gender, stress, decoding training, and stress reduction training on nonverbal decoding performance. *Dissertation Abstracts International, 44*(5-B), 1642.

575 Ross, P., & Flack, T. (2020). Removing hand form information specifically impairs emotion recognition for fearful and angry body stimuli. *Perception, 49*(1), 98–112.

6101 Rossetto, F., Baglio, F., Massaro, D., Alberoni, M., Nemni, R., Marchetti, A., & Castelli, I. (2020). Social cognition in rehabilitation context: Different evolution of affective and cognitive theory of mind in mild cognitive impairment. *Behavioural Neurology, 2020,* Article 5204927.

6036 Rosso, A. M., & Riolfo, A. (2020). A further look at Reading the Mind in the Eyes - Child version: Association with fluid intelligence, receptive language, and intergenerational transmission in typically developing school-aged children. *Frontiers in Psychology, 11,* Article 586065.

7477 Rotter, N. G., & Rotter, G. S. (1988). Sex differences in the encoding and decoding of negative facial emotions. *Journal of Nonverbal Behavior, 12*(2), 139–148.

5153 Ruben, M. A., & Hall, J. A. (2013). "I know your pain": Proximal and distal predictors of pain detection accuracy. *Personality and Social Psychology Bulletin, 39*(10), 1346–1358.

5661 Ruben, M. A., Stosic, M. D., Correale, J., & Blanch-Hartigan, D. (2021). Is technology enhancing or hindering interpersonal communication? A framework and preliminary results to examine the relationship between technology use and nonverbal decoding skill. *Frontiers in Psychology, 11,* Article 611670.

7182 Ruffman, T., Halberstadt, J., Murray, J., Jack, F., & Vater, T. (2020). Empathic accuracy: Worse recognition by older adults and less transparency in older adult expressions compared with young adults. *The Journals of Gerontology: Series B: Psychological Sciences and Social Sciences, 75*(8), 1658–1667.

1195 Rupp, C. I., Derntl, B., Osthaus, F., Kemmler, G., & Fleischhacker, W. W. (2017). Impact of social cognition on alcohol dependence treatment outcome: Poorer facial emotion recognition predicts relapse/dropout. *Alcoholism: Clinical and Experimental Research, 41*(12), 2197–2206.
312 Russell, L. L., Greaves, C. V., Bocchetta, M., Nicholas, J., Convery, R. S., Moore, K., Cash, D. M., van Swieten, J., Jiskoot, L., Moreno, F., Sanchez-Valle, R., Borroni, B., Laforce, R., Jr., Masellis, M., Tartaglia, M. C., Graff, C., Rotondo, E., Galimberti, D., Rowe, J. B., . . . Genetic FTD Initiative (GENFI). (2020). Social cognition impairment in genetic frontotemporal dementia within the GENFI cohort. *Cortex: A Journal Devoted to the Study of the Nervous System and Behavior, 133,* 384–398.
6586 Rutherford, H. J. V., Wareham, J. D., Vrouva, I., Mayes, L. C., Fonagy, P., & Potenza, M. N. (2012). Sex differences moderate the relationship between adolescent language and mentalization. *Personality Disorders: Theory, Research, and Treatment, 3*(4), 393–405.
902 Rutter, L. A. (2019). Emotion recognition in depression and anxiety. *Dissertation Abstracts International: Section B: The Sciences and Engineering, 80*(3-B(E)).
7567 Sabatelli, R. M., Buck, R., & Dreyer, A. (1980). Communication via facial cues in intimate dyads. *Personality and Social Psychology Bulletin, 6*(2), 242–247.
7442 Sabatelli, R. M., Buck, R., & Dreyer, A. (1982). Nonverbal communication accuracy in married couples: Relationship with marital complaints. *Journal of Personality and Social Psychology, 43*(5), 1088–1097.
6117 Sahbaz, C., & Kurtulmus, A. (2019). Association between emotional functioning and biological rhythm disruptions in patients with schizophrenia. *Psychiatry and Clinical Psychopharmacology, 29*(4), 455–462.
644 Sánchez-Reales, S., Caballero-Peláez, C., Prado-Abril, J., Inchausti, F., Lado-Codesido, M., García-Caballero, A., & Lahera, G. (2019). Spanish validation of the "Reading the Mind in the Voice" task: A study of complex emotion recognition in adults with autism spectrum conditions. *Research in Autism Spectrum Disorders, 67,* Article 101421.
1618 Sapey-Triomphe, L.-A., Heckemann, R. A., Boublay, N., Dorey, J.-M., Hénaff, M.-A., Rouch, I., Padovan, C., Hammers, A., Krolak-Salmon, P., & Alzheimer's Disease Neuroimaging Initiative. (2015). Neuroanatomical correlates of recognizing face expressions in mild stages of Alzheimer's disease. *PLoS ONE, 10*(12), Article e0143586.
7512 Sapienza, P., Zingales, L., & Maestripieri, D. (2009). Gender differences in financial risk aversion and career choices are affected by testosterone. *PNAS Proceedings of the National Academy of Sciences of the United States of America, 106*(36), 15268–15273.
7478 Sasson, N. J., Pinkham, A. E., Richard, J., Hughett, P., Gur, R. E., & Gur, R. C. (2010). Controlling for response biases clarifies sex and age differences in facial affect recognition. *Journal of Nonverbal Behavior, 34*(4), 207–221.
1535 Sasson, N. J., Pinkham, A. E., Weittenhiller, L. P., Faso, D. J., & Simpson, C. (2016). Context effects on facial affect recognition in schizophrenia and autism: Behavioral and eye-tracking evidence. *Schizophrenia Bulletin, 42*(3), 675–683.
6360 Sato, W., Kochiyama, T., Uono, S., Sawada, R., Kubota, Y., Yoshimura, S., & Toichi, M. (2016). Structural neural substrates of reading the mind in the eyes. *Frontiers in Human Neuroscience, 10,* Article 151.
1035 Saylik, R., Raman, E., & Szameitat, A. J. (2018). Sex differences in emotion recognition and working memory tasks. *Frontiers in Psychology, 9,* Article 1072.
7593 Scherer, K. R., Banse, R., & Wallbott, H. (2001). Emotion inferences from vocal expression correlate across languages and cultures. *Journal of Cross-Cultural Psychology, 32*, 76–92.
1130 Scherer, K. R., Mortillaro, M., Rotondi, I., Sergi, I., & Trznadel, S. (2018). Appraisal-driven facial actions as building blocks for emotion inference. *Journal of Personality and Social Psychology, 114*(3), 358–379.
5470 Scherer, K. R., & Scherer, U. (2011). Assessing the ability to recognize facial and vocal expressions of emotion: Construction and validation of the Emotion Recognition Index. *Journal of Nonverbal Behavior, 35*(4), 305–326.
291 Schild, A.-K., Volk, J., Scharfenberg, D., Schuermann, K., Meiberth, D., Onur, O. A., Jessen, F., & Maier, F. (2021). Social cognition in patients with amnestic mild cognitive impairment and mild dementia of the Alzheimer type. *Journal of Alzheimer's Disease, 83*(3), 1173–1186.
6174 Schimmenti, A., Jonason, P. K., Passanisi, A., La Marca, L., Di Dio, N., & Gervasi, A. M. (2019). Exploring the dark side of personality: Emotional awareness, empathy, and the Dark Triad traits in an Italian sample. *Current Psychology: A Journal for Diverse Perspectives on Diverse Psychological Issues, 38*(1), 100–109.

906 Schlegel, K., Fontaine, J. R. J., & Scherer, K. R. (2019). The nomological network of emotion recognition ability: Evidence from the Geneva Emotion Recognition Test. *European Journal of Psychological Assessment, 35*(3), 352–363.

1989 Schlegel, K., Grandjean, D., & Scherer, K. R. (2014). Introducing the Geneva Emotion Recognition Test: An example of Rasch-based test development. *Psychological Assessment, 26*(2), 666–672.

1050 Schlegel, K., Mehu, M., van Peer, J. M., & Scherer, K. R. (2018). Sense and sensibility: The role of cognitive and emotional intelligence in negotiation. *Journal of Research in Personality, 74,* 6–15.

815 Schlegel, K., & Mortillaro, M. (2019). The Geneva Emotional Competence Test (GECo): An ability measure of workplace emotional intelligence. *Journal of Applied Psychology, 104*(4), 559–580.

1240 Schlegel, K., Vicaria, I. M., Isaacowitz, D. M., & Hall, J. A. (2017). Effectiveness of a short audiovisual emotion recognition training program in adults. *Motivation and Emotion, 41*(5), 646–660.

7584 Schlegel, K., Witmer, J. S., & Rammsayer, T. H. (2017). Intelligence and sensory sensitivity as predictors of emotion recognition ability. *Journal of Intelligence, 5*(4), 35.

2026 Schmid, P. C., & Mast, M. S. (2010). Mood effects on emotion recognition. *Motivation and Emotion, 34*(3), 288–292.

5641 Schmid, P. C., Mast, M. S., Bombari, D., & Mast, F. W. (2011). Gender effects in information processing on a nonverbal decoding task. *Sex Roles: A Journal of Research, 65*(1-2), 102–107.

5026 Schmid Mast, M., & Darioly, A. (2014). Emotion recognition accuracy in hierarchical relationships. *Swiss Journal of Psychology, 73*(2), 69–75.

5735 Schmid Mast, M., Jonas, K., & Hall, J. A. (2009). Give a person power and he or she will show interpersonal sensitivity: The phenomenon and its why and when. *Journal of Personality and Social Psychology, 97*(5), 835–850.

5989 Schmidt, I., Rutanen, T., Luciani, R. S., & Jola, C. (2021). Feeling for the other with ease: Prospective actors show high levels of emotion recognition and report above average empathic concern, but do not experience strong distress. *Frontiers in Psychology, 12,* Article 543846.

417 Schmitt, H. S., Sindermann, C., Li, M., Ma, Y., Kendrick, K. M., Becker, B., & Montag, C. (2020). The dark side of emotion recognition—Evidence from cross-cultural research in Germany and China. *Frontiers in Psychology, 11,* Article 1132.

6341 Schneider, A., Johnston, C., Tassone, F., Sansone, S., Hagerman, R. J., Ferrer, E., Rivera, S. M., & Hessl, D. (2016). Broad autism spectrum and obsessive–compulsive symptoms in adults with the fragile X premutation. *The Clinical Neuropsychologist, 30*(6), 929–943.

7140 Schnellmann, J. (1986). The relation of dominance and gender in the decoding of nonverbal cues of affect. *Dissertation Abstracts International, 47*(1-B), 433.

5699 Schofield, G., Biggart, L., Ward, E., & Larsson, B. (2015). Looked after children and offending: An exploration of risk, resilience and the role of social cognition. *Children and Youth Services Review, 51,* 125–133.

2598 Scholten, M. R. M., Aleman, A., & Kahn, R. S. (2008). The processing of emotional prosody and semantics in schizophrenia: Relationship to gender and IQ. *Psychological Medicine, 38*(6), 887–898.

7479 Scholten, M. R. M., Aleman, A., Montagne, B., & Kahn, R. S. (2005). Schizophrenia and processing of facial emotions: Sex matters. *Schizophrenia Research, 78*(1), 61–67.

5615 Schwarzbold, M. L., Kern, R. S., Novacek, D. M., McGovern, J. E., Catalano, L. T., & Green, M. F. (2021). Self-stigma in psychotic disorders: Clinical, cognitive, and functional correlates in a diverse sample. *Schizophrenia Research, 228,* 145–150.

143 Scott, J. C., Moore, T. M., Stein, D. J., Pretorius, A., Zingela, Z., Nagdee, M., Ngqengelele, L., Campbell, M., Sibeko, G., King, M. C., McClellan, J. M., Port, A. M., Jackson, C., Ruparel, K., Susser, E. S., & Gur, R. C. (2021). Adaptation and validation of a computerized neurocognitive battery in the Xhosa of South Africa. *Neuropsychology, 35*(6), 581–594.

5658 Sellbom, M., Anderson, J. L., Goodwin, B. E., Kastner, R. M., Rock, R. C., Johnson, A. K., Meier, B. P., & Salekin, R. T. (2022). Evaluation of the moderated-expression and differential configuration hypotheses in the context of “successful” or “noncriminal” psychopathy. *Personality Disorders: Theory, Research, and Treatment, 13*(5), 542–556.

871 Shakeel, M. K., Lu, L., Cannon, T. D., Cadenhead, K. S., Cornblatt, B. A., McGlashan, T. H., Perkins, D. O., Seidman, L. J., Tsuang, M. T., Woods, S. W., Walker, E. F., Mathalon, D. H., Bearden, C. E., & Addington, J. (2019). Longitudinal changes in social cognition in individuals at clinical high risk for psychosis: An outcome based analysis. *Schizophrenia Research, 204,* 334–336.

1761 Sharp, C., Vanwoerden, S., Van Baardewijk, Y., Tackett, J. L., & Stegge, H. (2015). Callous-unemotional traits are associated with deficits in recognizing complex emotions in preadolescent children. *Journal of Personality Disorders, 29*(3), 347–359.

6424 Sherman, G. D., Lerner, J. S., Renshon, J., Ma-Kellams, C., & Joel, S. (2015). Perceiving others' feelings: The importance of personality and social structure. *Social Psychological and Personality Science, 6*(5), 559–569.

645 Shiota, M. N., Simpson, M. L., Kirsch, H. E., & Levenson, R. W. (2019). Emotion recognition in objects in patients with neurological disease. *Neuropsychology, 33*(8), 1163–1173.

1052 Shukla, M., Pandey, R., Jain, D., & Lau, J. Y. F. (2018). Poor emotional responsiveness in clinical hypertension: Reduced accuracy in the labelling and matching of emotional faces amongst individuals with hypertension and prehypertension. *Psychology & Health, 33*(6), 765–782.

816 Shukla, M., Pandey, R., & Lau, J. Y. F. (2019). Assessing emotional processing difficulties in normotensive individuals with high and isolated blood pressure elevations. *International Journal of Psychology, 54*(2), 214–222.

46 Siew, S. K. H., Han, M. F. Y., Mahendran, R., & Yu, J. (2022). Regression-based norms and validation of the Cambridge Neuropsychological Test Automated Battery among community-living older adults in Singapore. *Archives of Clinical Neuropsychology, 37*(2), 457–472.

6020 Sijtsma, H., Lee, N. C., Hollarek, M., Walsh, R. J., van Buuren, M., Braams, B. R., & Krabbendam, L. (2021). Social cognition and friendships in adolescents with autistic-like experiences and psychotic-like experiences. *Frontiers in Psychiatry, 11,* Article 589824.

579 Silk, J. S., Pramana, G., Sequeira, S. L., Lindhiem, O., Kendall, P. C., Rosen, D., & Parmanto, B. (2020). Using a smartphone app and clinician portal to enhance brief cognitive behavioral therapy for childhood anxiety disorders. *Behavior Therapy, 51*(1), 69–84.

2618 Simão, C. P., Justo, M. G., & Martins, A. T. (2008). Recognizing facial expressions of social emotions: Do males and females differ? *Psicologia: Revista da Associação Portuguesa Psicologia, 22*(2), 71–85.

7569 Simon, L. J., Francis, P. L., & Lombardo, J. P. (1990). Sex, sex-role, and Machiavellianism as correlates of decoding ability. *Perceptual and Motor Skills, 71*(1), 243–247.

6699 Smeets, T., Dziobek, I., & Wolf, O. T. (2009). Social cognition under stress: Differential effects of stress-induced cortisol elevations in healthy young men and women. *Hormones and Behavior, 55*(4), 507–513.

6263 Smith, K. E, Norman, G. J, & Decety, J. (2017). The complexity of empathy during medical school training: Evidence for positive changes. *Medical Education, 51*(11), 1146–1159.

7029 Snodgrass, S. E., & Rosenthal, R. (1985). Interpersonal sensitivity and skills in decoding nonverbal channels: The value of face value. *Basic and Applied Social Psychology, 6*(3), 243–255.

377 Soker-Elimaliah, S., Jennings, C. A., Hashimi, M. M., Cassim, T. Z., Lehrfield, A., & Wagner, J. B. (2020). Autistic traits moderate relations between cardiac autonomic activity, interoceptive accuracy, and emotion processing in college students. *International Journal of Psychophysiology, 155,* 118–126.

5122 Spikman, J. M., Boelen, D. H. E., Pijnenborg, G. H. M., Timmerman, M. E., van der Naalt, J., & Fasotti, L. (2013). Who benefits from treatment for executive dysfunction after brain injury? Negative effects of emotion recognition deficits. *Neuropsychological Rehabilitation, 23*(6), 824–845.

7513 Spreng, R. N., McKinnon, M. C., Mar, R. A., & Levine, B. (2009). The Toronto Empathy Questionnaire: Scale development and initial validation of a factor-analytic solution to multiple empathy measures. *Journal of Personality Assessment, 91*(1), 62–71.

6136 Stagg, S. D., & Vincent, J. (2019). Autistic traits in individuals self-defining as transgender or nonbinary. *European Psychiatry, 61,* 17–22.

1241 Statucka, M., & Walder, D. J. (2017). Facial affect recognition and social functioning among individuals with varying degrees of schizotypy. *Psychiatry Research, 256,* 180–187.

6158 Stewart, S. L. K., Wright, C., & Atherton, C. (2019). Deception detection and truth detection are dependent on different cognitive and emotional traits: An investigation of emotional intelligence, theory of mind, and attention. *Personality and Social Psychology Bulletin, 45*(5), 794–807.

245 Stiekema, A. P. M., Nijsse, B., de Kort, P. L. M., Spikman, J. M., Visser-Meily, J. M. A., & van Heugten, C. M. (2021). The relationship between social cognition and participation in the long term after stroke. *Neuropsychological Rehabilitation, 31*(2), 278–292.

7037 Stokes, D. R. (1984). Nonverbal communication: Race, gender, social class, world view and the PONS test; Implications for the therapeutic dyad. *Dissertation Abstracts International, 44*(11-B), 3544.

9006 Stosic, M. D. (2021). Unpublished data, University of Maine.

8012 Stosic, M. D., Blanch-Hartigan, D., Aleksanyan, T., Duenas, J., & Ruben, M. A. (2021). Empathy, friend or foe? Untangling the relationship between empathy and burnout in helping professions. *The Journal of Social Psychology*, *162*(1), 89-108.

8011 Stosic, M. D., Fultz, A. A., Brown, J. & Bernieri, F. J. (2021). What is your empathy scale not measuring? The convergent, discriminant, and predictive validity of five empathy scales. *The Journal of Social Psychology, 162*(1), 7-25.

8010 Stosic, M. D., Scarpulla, E., Weaver A. E., & Ruben M. A. (2023). Should I post? The relationships among social media use, emotion recognition, and mental health. *Frontiers in Psychology*, *14*, 1161300.

673 Strnádelová, B., Halamová, J., & Kanovský, M. (2019). Identification of primary facial emotions in relation to level of self-criticism. *Perception, 48*(10), 948–967.

1242 Stubberud, J. (2017). Theory of mind in spina bifida: Relationship with intellectual and executive functioning. *Scandinavian Journal of Psychology, 58*(5), 379–388.

5319 Sucksmith, E., Allison, C., Baron-Cohen, S., Chakrabarti, B., & Hoekstra, R. A. (2013). Empathy and emotion recognition in people with autism, first-degree relatives, and controls. *Neuropsychologia, 51*(1), 98–105.

1075 Sun, M., & Lau, A. S. (2018). Exploring cultural differences in expressive suppression and emotion recognition. *Journal of Cross-Cultural Psychology, 49*(4), 664–672.

1198 Sutcliffe, R., Rendell, P. G., Henry, J. D., Bailey, P. E., & Ruffman, T. (2017). Music to my ears: Age-related decline in musical and facial emotion recognition. *Psychology and Aging, 32*(8), 698–709.

6269 Svedholm-Häkkinen, A. M., & Lindeman, M. (2017). Intuitive and deliberative empathizers and systemizers. *Journal of Personality, 85*(5), 593–602.

1555 Svetieva, E., & Frank, M. G. (2016). Empathy, emotion dysregulation, and enhanced microexpression recognition ability. *Motivation and Emotion, 40*(2), 309–320.

7740 Swagerman, S. C., de Geus, E. J. C., Kan, K.-J., van Bergen, E., Nieuwboer, H. A., Koenis, M. M. G., Hulshoff Pol, H. E., Gur, R. E., Gur, R. C., & Boomsma, D. I. (2016). The Computerized Neurocognitive Battery: Validation, aging effects, and heritability across cognitive domains. *Neuropsychology, 30*(1), 53–64.

5925 Swain, R. H., O'Hare, A. J., Brandley, K., & Gardner, A. T. (2022). Individual differences in social intelligence and perception of emotion expression of masked and unmasked faces. *Cognitive Research: Principles and Implications, 7,* Article 54.

7521 Sweeney, M. A., & Cottle, W. C. (1976). Nonverbal acuity: A comparison of counselors and noncounselors. *Journal of Counseling Psychology, 23*, 394- 397.

7487 Sweeney, M. A., Cottle, W. C., & Kobayashi, M. J. (1980). Nonverbal communication: A cross-cultural comparison of American and Japanese counseling students. *Journal of Counseling Psychology, 27*(2), 150–156.

7570 Swenson, J., & Casmir, F. L. (1998). The impact of culture-sameness, gender, foreign travel, and academic background on the ability to interpret facial expression of emotion in others. *Communication Quarterly, 46*, 214–230.

6697 Szily, E., & Kéri, S. (2009). Anomalous subjective experience and psychosis risk in young depressed patients. *Psychopathology, 42*(4), 229–235.

6195 Tabullo, A. J., Navas Jiménez, V. A., & García, C. S. (2018). Associations between fiction reading, trait empathy and theory of mind ability. *International Journal of Psychology & Psychological Therapy, 18*(3), 357–370.

6062 Tahazadeh, S., Barahmand, U., Yaghooti, F., & Nazari, M. A. (2020). Mind reading in films task to assess social cognitive deficits in autism spectrum conditions. *Journal of Evidence-Based Psychotherapies, 20*(2), 79–100.

6995 Tarver, M. L. (1996). Nonverbal social perception of community college students with and without learning disabilities. *Dissertation Abstracts International Section A: Humanities and Social Sciences, 56*(9-A), 3544.

5097 Teague, E. B. (2014). Emotional expression and perception in three ethnic groups: Is there an in-group advantage? *Dissertation Abstracts International: Section B: The Sciences and Engineering, 75*(2-B(E)).

7489 Terracciano, A., Merritt, M., Zonderman, A. B., & Evans, M. K. (2003). Personality traits and sex differences in emotion recognition among African Americans and Caucasians*. Annals of the New York Academy of Sciences, 1000*, 309–312.

5406 Thingujam, N. S., Laukka, P., & Elfenbein, H. A. (2012). Distinct emotional abilities converge: Evidence from emotional understanding and emotion recognition through the voice. *Journal of Research in Personality, 46*(3), 350–354.

6764 Thorstenson, C. A., Pazda, A. D., & Krumhuber, E. G. (2021). The influence of facial blushing and paling on emotion perception and memory. *Motivation and Emotion, 45*(6), 818–830.

7323 Tipsord, J. M. (2009). The effects of mindfulness training and individual differences in mindfulness on social perception and empathy. *Dissertation Abstracts International: Section B: The Sciences and Engineering, 70*(11-B), 7273.

5958 Trainin, N., & Yeshurun, Y. (2021). Reading the mind with a mask? Improvement in reading the mind in the eyes during the COVID-19 pandemic. *Emotion, 21*(8), 1801–1806.

6118 Turan, S., Özyurt, G., Çatlı, G., Öztürk, Y., Abacı, A., & Akay, A. P. (2019). Social cognition and emotion regulation may be impaired in adolescents with obesity independent of the presence of binge eating disorder: A two-center study. *Psychiatry and Clinical Psychopharmacology, 29*(4), 887–894.

2767 Turkstra, L. S., McDonald, S., & DePompei, R. (2001). Social information processing in adolescents: Data from normally developing adolescents and preliminary data from their peers with traumatic brain injury. *The Journal of Head Trauma Rehabilitation, 16*(5), 469–483.

7577 Turner, I. (2016). Development of a complex theory of mind task: Reading the Mind in film-American. Doctoral dissertation, University of South Alabama, Mobile, AL.

5875 Udayar, S., Fiori, M., & Bausseron, E. (2020). Emotional intelligence and performance in a stressful task: The mediating role of self-efficacy. *Personality and Individual Differences, 156,* Article 109790.

540 Ünal-Aydın, P., Balıkçı, K., Sönmez, İ., & Aydın, O. (2020). Associations between emotion recognition and social networking site addiction. *Psychiatry Research, 284,* Article 112673.

248 Ünal-Aydın, P., Obuća, F., Aydın, O., & Spada, M. M. (2021). The role of metacognitions emotion recognition in problematic SNS use among adolescents. *Journal of Affective Disorders, 282,* 1–8.

7585 Valadez-Sierra, M. D., Borges del Rosal, M. A., Ruvalcaba Romero, N., Villegas, K., & Lorenzo, M. (2013). Emotional intelligence and its relationship with gender, academic performance and intellectual abilities of undergraduates. *Electronic Journal of Research in Educational Psychology, 11*(2), 395–412.

96 Valera-Bermejo, J. M., De Marco, M., Mitolo, M., Cerami, C., Dodich, A., & Venneri, A. (2021). Large-scale functional networks, cognition and brain structures supporting social cognition and theory of mind performance in prodromal to mild Alzheimer's disease. *Frontiers in Aging Neuroscience, 13,* Article 766703.

6671 Valla, J. M., Ganzel, B. L., Yoder, K. J., Chen, G. M., Lyman, L. T., Sidari, A. P., Keller, A. E., Maendel, J. W., Perlman, J. E., Wong, S. K. L., & Belmonte, M. K. (2010). More than maths and mindreading: Sex differences in empathizing/systemizing covariance. *Autism Research, 3*(4), 174–184.

6860 van den Berg, N. S., Lammers, N. A., Smits, A. R., Lugtmeijer, S., Pinto, Y., & De Haan, E. H. F. (2022). Mid-range visual functions in relation to higher-order visual functions after stroke. *Journal of Clinical and Experimental Neuropsychology, 44*(8), 580–591.

6290 van der Meulen, A., Roerig, S., de Ruyter, D., van Lier, P., & Krabbendam, L. (2017). A comparison of children's ability to read children's and adults' mental states in an adaptation of the Reading the Mind in the Eyes task. *Frontiers in Psychology, 8,* Article 594.

7556 Van Rooijen, L. (1973). Talking about the bright side . . . . Pleasantness of the referent as a determinant of communication accuracy. *European Journal of Social Psychology, 3*(4), 473–478.

1137 van Rooijen, R., Junge, C. M. M., & Kemner, C. (2018). The interplay between gaze following, emotion recognition, and empathy across adolescence; a pubertal dip in performance? *Frontiers in Psychology, 9,* Article 127.

915 van Zonneveld, L., de Sonneville, L., van Goozen, S., & Swaab, H. (2019). Recognition of facial emotion and affective prosody in children at high risk of criminal behavior. *Journal of the International Neuropsychological Society, 25*(1), 57–64.

6544 van Zwieten, A., Meyer, J., Hermens, D. F., Hickie, I. B., Hawes, D. J., Glozier, N., Naismith, S. L., Scott, E. M., Lee, R. S. C., & Guastella, A. J. (2013). Social cognition deficits and psychopathic traits in young people seeking mental health treatment. *PLoS ONE, 8*(7), Article e67753.

5974 Varela, L. F., Wong, K. H. T., Shergill, S. S., & Fett, A.-K. J. (2021). Attachment styles moderate Theory of Mind differences between persons with schizophrenia, first-degree relatives and controls. *British Journal of Clinical Psychology, 60*(3), 339–356.

1259 Vasconcelos, M., Dias, M., Soares, A. P., & Pinheiro, A. P. (2017). What is the melody of that voice? Probing unbiased recognition accuracy with the Montreal affective voices. *Journal of Nonverbal Behavior, 41*(3), 239–267.

720 Vaskinn, A., Løvgren, A., Egeland, M. K., Feyer, F. K., Østefjells, T., Andreassen, O. A., Melle, I., & Sundet, K. (2019). A randomized controlled trial of training of affect recognition (TAR) in schizophrenia shows lasting effects for theory of mind. *European Archives of Psychiatry and Clinical Neuroscience, 269*(5), 611–620.

7495 Vassallo, S., Cooper, S. L., & Douglas, J. M. (2009). Visual scanning in the recognition of facial affect: Is there an observer sex difference? *Journal of Vision, 9*(3), 1–10.

6551 Vellante, M., Baron-Cohen, S., Melis, M., Marrone, M., Petretto, D. R., Masala, C., & Preti, A. (2013). The "Reading the Mind in the Eyes" test: Systematic review of psychometric properties and a validation study in Italy. *Cognitive Neuropsychiatry, 18*(4), 326–354.

6993 Vencil, M. J. (1997). The relationship of adolescents' actual and perceived nonverbal communication skills to social status. *Dissertation Abstracts International: Section B: The Sciences and Engineering, 57*(12-B), 7746.

610 Verpaalen, I. A. M., Bijsterbosch, G., Mobach, L., Bijlstra, G., Rinck, M., & Klein, A. M. (2019). Validating the Radboud faces database from a child's perspective. *Cognition and Emotion, 33*(8), 1531–1547.

1037 Vetter, N. C., Drauschke, M., Thieme, J., & Altgassen, M. (2018). Adolescent basic facial emotion recognition is not influenced by puberty or own-age bias. *Frontiers in Psychology, 9,* Article 956.

937 Vidal-Ribas, P., Brotman, M. A., Salum, G. A., Kaiser, A., Meffert, L., Pine, D. S., Leibenluft, E., & Stringaris, A. (2018). Deficits in emotion recognition are associated with depressive symptoms in youth with disruptive mood dysregulation disorder. *Depression and Anxiety, 35*(12), 1207–1217.

1427 Visser-Keizer, A. C., Westerhof-Evers, H. J., Gerritsen, M. J. J., van der Naalt, J., & Spikman, J. M. (2016). To fear is to gain? The role of fear recognition in risky decision making in TBI patients and healthy controls. *PLoS ONE, 11*(11), Article e0166995.

1038 Vizeli, P., & Liechti, M. E. (2018). Oxytocin receptor gene variations and socioemotional effects of MDMA: A pooled analysis of controlled studies in healthy subjects. *PLoS ONE, 13*(6), Article e0199384.

6392 Vonk, J., Zeigler-Hill, V., Ewing, D., Mercer, S., & Noser, A. E. (2015). Mindreading in the dark: Dark personality features and theory of mind. *Personality and Individual Differences, 87,* 50–54.

5291 Vonk, J., Zeigler-Hill, V., Mayhew, P., & Mercer, S. (2013). Mirror, mirror on the wall, which form of narcissist knows self and others best of all? *Personality and Individual Differences, 54*(3), 396–401.

6713 Voracek, M., & Dressler, S. G. (2006). Lack of correlation between digit ratio (2D:4D) and Baron-Cohen's "Reading the Mind in the Eyes" test, empathy, systemising, and autism-spectrum quotients in a general population sample. *Personality and Individual Differences, 41*(8), 1481–1491.

1443 Vrijen, C., Hartman, C. A., & Oldehinkel, A. J. (2016). Slow identification of facial happiness in early adolescence predicts onset of depression during 8 years of follow-up. *European Child & Adolescent Psychiatry, 25*(11), 1255–1266.

1269 Wagner, K. E., Kates, W. R., Fremont, W., & Antshel, K. M. (2017). Childhood predictors of young adult social functioning in 22q11.2 deletion syndrome. *Journal of Autism and Developmental Disorders, 47*(8), 2480–2501.

6752 Wagner, M. F., Milner, J. S., McCarthy, R. J., Crouch, J. L., McCanne, T. R., & Skowronski, J. J. (2015). Facial emotion recognition accuracy and child physical abuse: An experiment and a meta-analysis. *Psychology of Violence, 5*(2), 154–162.

5645 Wakabayashi, A., & Katsumata, A. (2011). The Motion Picture Mind-Reading Test: Measuring individual differences of social cognitive ability in a young adult population in Japan. *Journal of Individual Differences, 32*(2), 55–64.

1015 Waller, R., McCabe, H. K., Dotterer, H. L., Neumann, C. S., & Hyde, L. W. (2018). Unique and interactive associations between maltreatment and complex emotion recognition deficits and psychopathic traits in an undergraduate sample. *Journal of Personality Disorders, 32*(4), 543–561.

6127 Walsh-Messinger, J., Stepanek, C., Wiedemann, J., Goetz, D., Goetz, R. R, & Malaspina, D. (2019). Normal sexual dimorphism in theory of mind circuitry is reversed in schizophrenia. *Social Neuroscience, 14*(5), 583–593.

5361 Walter, F., Cole, M. S., van der Vegt, G. S., Rubin, R. S., & Bommer, W. H. (2012). Emotion recognition and emergent leadership: Unraveling mediating mechanisms and boundary conditions. *The Leadership Quarterly, 23*(5), 977–991.

5625 Walther, S., Stegmayer, K., Sulzbacher, J., Vanbellingen, T., Müri, R., Strik, W., & Bohlhalter, S. (2015). Nonverbal social communication and gesture control in schizophrenia. *Schizophrenia Bulletin, 41*(2), 338–345.

6196 Walzak, L. C., & Loken Thornton, W. (2018). The role of illness burden in theory of mind performance among older adults. *Experimental Aging Research, 44*(5), 427–442.

93 Wang, J., Shi, X., Zou, H., Pons, F., Xu, Q., Wang, Y., Tang, Y., & Jiang, S. (2021). Development and validation of the Emotional Intelligence Test for Adolescents in a Chinese sample. *Psychological Assessment, 33*(12), 1200–1214.

5872 Wang, M. Z., Chen, K., & Hall, J. A. (2021). Predictive validity of thin slices of verbal and nonverbal behaviors: Comparison of slice lengths and rating methodologies. *Journal of Nonverbal Behavior, 45*(1), 53–66.

9001 Wang, M. Z., & Hall, J. A. (2020). Unpublished data, Northeastern University.

6358 Wang, X., Song, Y., Zhen, Z., & Liu, J. (2016). Functional integration of the posterior superior temporal sulcus correlates with facial expression recognition. *Human Brain Mapping, 37*(5), 1930–1940.
6128 Warnell, K. R., & Redcay, E. (2019). Minimal coherence among varied theory of mind measures in childhood and adulthood. *Cognition, 191,* Article 103997.
6218 Warrier, V., Grasby, K. L., Uzefovsky, F., Toro, R., Smith, P., Chakrabarti, B., Khadake, J., Mawbey-Adamson, E., Litterman, N., Hottenga, J. -J., Lubke, G., Boomsma, D. I., Martin, N. G., Hatemi, P. K., Medland, S. E., Hinds, D. A., Bourgeron, T., & Baron-Cohen, S. (2018). Genome-wide meta-analysis of cognitive empathy: Heritability, and correlates with sex, neuropsychiatric conditions and cognition. *Molecular Psychiatry, 23*(6), 1402–1409.
6973 Weaver, M. A. (2011). Making decisions about genetic testing: Understanding the process in prenatal and cancer genetic contexts. *Dissertation Abstracts International: Section B: The Sciences and Engineering, 72*(3-B), 1448.
5054 Weisenbach, S. L., Rapport, L. J., Bricen, E. M., Haase, B. D., Vederman, A. C., Bieliauskas, L. A., Welsh, R. C., Starkman, M. N., McInnis, M. G., Zubieta, J.-K., & Langenecker, S. A. (2014). Reduced emotion processing efficiency in healthy males relative to females. *Social Cognitive and Affective Neuroscience, 9*(3), 316–325.
7518 Weisgerber, C. A. Accuracy in judging emotional expressions as related to college entrance test scores. Journal of Social Psychology, 1956, 44, 233-239.
7498 Weisgerber, C. A. (1957). Accuracy in judging emotional expressions as related to understanding of literature. *The Journal of Social Psychology, 46,* 253–258.
6417 Weisman, O., Pelphrey, K. A., Leckman, J. F., Feldman, R., Lu, Y., Chong, A., Chen, Y., Monakhov, M., Chew, S. H., & Ebstein, R. P. (2015). The association between 2D:4D ratio and cognitive empathy is contingent on a common polymorphism in the oxytocin receptor gene (*OXTR* rs53576). *Psychoneuroendocrinology, 58,* 23–32.
314 Wells, A. E., Hunnikin, L. M., Ash, D. P., & van Goozen, S. H. M. (2020). Low self-esteem and impairments in emotion recognition predict behavioural problems in children. *Journal of Psychopathology and Behavioral Assessment, 42*(4), 693–701.
7558 Westbrook, M. (1974). Judgement of emotion: Attention versus accuracy. *British Journal of Social & Clinical Psychology, 13*(4), 383–389.
8007 Wever, M. C. M., van Houtum, L., Janssen, L. H. C., Spruit, I. M., Tollenaar, M. S., aan het Rot, M., & Elzinga, B. M. (2022). Eyes on you: Ensuring empathic accuracy or signalling empathy? *International Journal of Psychology, 57*(6), 743-752.
5662 White, R., Russell, G., Qualter, P., Owens, M., & Psychogiou, L. (2021). Do peer relationships mediate the association between children's facial emotion recognition ability and their academic attainment? Findings from the ALSPAC study. *Contemporary Educational Psychology, 64,* Article 101942.
2551 Wickline, V. B., Bailey, W., & Nowicki, S. (2009). Cultural in-group advantage: Emotion recognition in African American and European American faces and voices. *The Journal of Genetic Psychology: Research and Theory on Human Development, 170*(1), 5–29.
265 Wieck, C., Kunzmann, U., & Scheibe, S. (2021). Empathy at work: The role of age and emotional job demands. *Psychology and Aging, 36*(1), 36–48.
5683 Willard, V. W., Allen, T. M., Hardy, K. K., & Bonner, M. J. (2017). Social functioning in survivors of pediatric brain tumors: Contribution of neurocognitive and social-cognitive skills. *Children's Health Care, 46*(2), 181–195.
5975 Wimmer, L., Currie, G., Friend, S., & Ferguson, H. J. (2021). Testing correlates of lifetime exposure to print fiction following a multi-method approach: Evidence from young and older readers. *Imagination, Cognition and Personality, 41*(1), 54–86.
1779 Wingenbach, T. S. H., Ashwin, C., & Brosnan, M. (2018). Sex differences in facial emotion recognition across varying expression intensity levels from videos. *PLoS ONE, 13*(1), Article e0190634.
6457 Wlodarski, R., & Dunbar, R. I. M. (2014). The effects of romantic love on mentalizing abilities. *Review of General Psychology, 18*(4), 313–321.
5976 Wójciak, P., Domowicz, K., Andrzejewska, M., & Rybakowski, J. K. (2021). Negative symptoms in schizophrenia, assessed by the Brief Negative Symptom Scale, Self-evaluation of Negative Symptom Scale, and social cognition: A gender effect. *International Journal of Psychiatry in Clinical Practice, 25*(3), 252–257.
701 Won, S., Lee, W. K., Kim, S.-W., Kim, J. J., Lee, B. J., Yu, J.-C., Lee, K. Y., Lee, S.-H., Kim, S.-H., Kang, S. H., Kim, E., & Chung, Y.-C. (2019). Distinct differences in emotional recognition according to severity of psychotic symptoms in early-stage schizophrenia. *Frontiers in Psychiatry, 10,* Article 564.

6000 Wood-Downie, H., Wong, B., Kovshoff, H., Mandy, W., Hull, L., & Hadwin, J. A. (2021). Sex/gender differences in camouflaging in children and adolescents with autism. *Journal of Autism and Developmental Disorders, 51*(4), 1353–1364.

5742 Woods, S., Wolke, D., Nowicki, S., & Hall, L. (2009). Emotion recognition abilities and empathy of victims of bullying. *Child Abuse & Neglect, 33*(5), 307–311.

1149 Wright, R., Riedel, R., Sechrest, L., Lane, R. D., & Smith, R. (2018). Sex differences in emotion recognition ability: The mediating role of trait emotional awareness. *Motivation and Emotion, 42*(1), 149–160.

7587 Wright, S. L., Langenecker, S. A., Deldin, P. J., Rapport, L. J., Nielson, K. A., Kade, A. M., Own, L. S., Akil, H., Young, E. A., & Zubieta, J.-K. (2009). Gender-specific disruptions in emotion processing in younger adults with depression. *Depression and Anxiety, 26*(2), 182–189.

963 Yap, V. M. Z., McLachlan, N. M., Scheffer, I. E., & Wilson, S. J. (2018). Enhanced sensitivity to angry voices in people with features of the broader autism phenotype. *Journal of Autism and Developmental Disorders, 48*(11), 3899–3911.

1057 Yıldırım, E., Yalınçetin, B., Sevilmiş, Ş., Kutay, Ö., & Alptekin, K. (2018). Is there any relation between impaired emotion perception and thought disorder in schizophrenia? *Nöropsikiyatri Arşivi, 55*(2), 118–122.

6761 Yitzhak, N., Pertzov, Y., Guy, N., & Aviezer, H. (2022). Many ways to see your feelings: Successful facial expression recognition occurs with diverse patterns of fixation distributions. *Emotion, 22*(5), 844–860.

495 Yoshie, M., & Sauter, D. A. (2020). Cultural norms influence nonverbal emotion communication: Japanese vocalizations of socially disengaging emotions. *Emotion, 20*(3), 513–517.

5832 Yu, E. H., Choi, E. J., Lee, S. Y., Im, S. J., Yune, S. J., & Baek, S. Y. (2016). Effects of micro- and subtle-expression reading skill training in medical students: A randomized trial. *Patient Education and Counseling, 99*(10), 1670–1675.

6078 Yu, S., & Kilduff, G. J. (2020). Knowing where others stand: Accuracy and performance effects of individuals' perceived status hierarchies. *Journal of Personality and Social Psychology, 119*(1), 159–184.

6237 Zainal, N. H., & Newman, M. G. (2018). Worry amplifies theory-of-mind reasoning for negatively valenced social stimuli in generalized anxiety disorder. *Journal of Affective Disorders, 227,* 824–833.

1102 Zarotti, N., Simpson, J., Fletcher, I., Squitieri, F., & Migliore, S. (2018). Exploring emotion regulation and emotion recognition in people with presymptomatic Huntington's disease: The role of emotional awareness. *Neuropsychologia, 112,* 1–9.

62 Zekelman, L. R., Zhang, F., Makris, N., He, J., Chen, Y., Xue, T., Liera, D., Drane, D. L., Rathi, Y., Golby, A. J., & O'Donnell, L. J. (2022). White matter association tracts underlying language and theory of mind: An investigation of 809 brains from the Human Connectome Project. *NeuroImage, 246,* Article 118739.

6222 Zhang, T., Cui, H., Wei, Y., Tang, Y., Xu, L., Tang, X., Zhu, Y., Jiang, L., Zhang, B., Qian, Z., Chow, A., Liu, X., Li, C., Xiao, Z., & Wang, J. (2018). Progressive decline of cognition during the conversion from prodrome to psychosis with a characteristic pattern of the theory of mind compensated by neurocognition. *Schizophrenia Research, 195,* 554–559.

1526 Zhang, L., Song, Y., Liu, L., & Liu, J. (2016). Dissociable roles of internal feelings and face recognition ability in facial expression decoding. *NeuroImage, 132,* 283–292.

6160 Zhao, J., Yang, Y., Li, X., Zheng, L., Xue, M., Zhang, M., Wang, C., Yu, R., & Gong, P. (2019). *OXTR* rs53576 polymorphism impacts interpersonal adaptability: Dispositional forgiveness as a mediator. *Psychoneuroendocrinology, 103,* 8–13.

5920 Zhao, Z., Thornton, M. A., & Tamir, D. I. (2022). Accurate emotion prediction in dyads and groups and its potential social benefits. *Emotion, 22*(5), 1030–1043.

5453 Zhu, B., Chen, C., Moyzis, R. K., Dong, Q., Chen, C., He, Q., Stern, H. S., Li, H., Jin, L., Li, J., Lessard, J., & Lin, C. (2012). Genetic variations in the dopamine system and facial expression recognition in healthy Chinese college students. *Neuropsychobiology, 65*(2), 83–89.

1587 Zimmerman, D. L., Ownsworth, T., O'Donovan, A., Roberts, J., & Gullo, M. J. (2016). Independence of hot and cold executive function deficits in high-functioning adults with autism spectrum disorder. *Frontiers in Human Neuroscience, 10,* Article 24.

7560 Zuckerman, M., Hall, J. A., DeFrank, R. S., & Rosenthal, R. (1976). Encoding and decoding of spontaneous and posed facial expressions. *Journal of Personality and Social Psychology, 34*(5), 966–977.

7559 Zuckerman, M., Larrance, D. T., Hall, J. A., DeFrank, R. S., & Rosenthal, R. (1979). Posed and spontaneous communication of emotion via facial and vocal cues. *Journal of Personality, 47*(4), 712–733.

1359 Zupan, B., Babbage, D., Neumann, D., & Willer, B. (2017). Sex differences in emotion recognition and emotional inferencing following severe traumatic brain injury. *Brain Impairment, 18*(1), 36–48.

Zwick, J. C., & Wolkenstein, L. (2017). Facial emotion recognition, theory of mind and the role of facial mimicry in depression. *Journal of Affective Disorders, 210,* 90–99.
