# Supplementary material for: Gender and Accuracy in Decoding Affect Cues: A Meta-Analysis
Source: J Intell. 2025 Mar 18;13(3):38. doi: 10.3390/jintelligence13030038 (PMC11943105; doi:10.3390/jintelligence13030038)
Supplement: Supplementary file 1 [file jintelligence-13-00038-s001.zip › S3 Codebook for studies in gender and affect meta-analysis.pdf]

S3 Codebook for “Gender and Accuracy in Decoding Affect Cues: A Meta-Analysis”

Note: “ES” means effect size. Some variable names have been changed in the final manuscript.

Independent_study
A given study might have several effect sizes (ES), each on a different line in SPSS. One line has the “independent study” code = 1, and the others have code = 0. This variable was created in order to count how many independent studies are in the database.

Source_ID
Every source (article, book, etc.) has a unique ID number.

Source_count
This variable was created in order to count how many unique sources are in the database. A given source may occupy several lines because of multiple studies and/or multiple ES, but the source count is entered only once.

Study_ID
Every study (independent group) has a unique ID number.

Effect_size_ID
Every effect size (Pearson r, rz) has a unique ID number.

Citation
First author and year

Search_method
1= PsycInfo
2= SPSP listserve unpublished, or serendipity unpublished
4= bibliographies of articles and meta-analyses (note, we searched systematically bibliographies of gender meta-analyses, but we didn’t do this for bibliographies of individual sources, or search their Introduction for references to gender differences)
5= books or other works we have or run across (for use with the 88 serendipity code)

Search_term_used
1= (gender OR sex OR men OR male) AND “emotion recognition” AND (test OR task); then filtered on Quantitative Study. For studies published 2002 and earlier, the term is ‘Empirical Study.’
2= PONS test OR Profile of Nonverbal Sensitivity, all forms (from search, not from PONS monograph [Rosenthal et al., 1979], as that did not come up in the search; results from PONS monograph are coded as “serendipity” with code 88)
3= DANVA OR Diagnostic Assessment of Nonverbal Accuracy
4=METT OR SETT
5= JACFEE OR Japanese and Caucasian Facial Expressions of Emotion OR JACBART
6= Geneva Emotion Recognition Test
7= Multimodal Emotion Recognition Test
8= Reading the Mind in the Eyes

9= Reading the Mind in the Voice
10= Reading the Mind in Films
11= Radboud Faces (Langner '10)
12= ADFES (Amsterdam Dynamic Facial Expression Set)
13= Karolinska Directed Emotional Faces
14= NimStim
15= BLERT (Bell-Lysaker Emotion Recognition Test)
16= Montreal Set of Facial Displays of Emotion
17= Chinese Facial Affective Picture System
18= Korea University Facial Expression Collection, second edition (KUFEC-II) and Korean Facial Expression (KUFEC-I) (Kim et al '11)
19= TASIT-EET (Part 1, Emotion Recognition) (The Awareness of Social Inference Test)
20= FEEST (these are Ekman POFA faces)
21= Mini-SEA (Part 1, Emotion Recognition) (these are Ekman faces)
22= Pictures of Facial Affect (POFA)
23= nonverbal sensitivity
24= decoding nonverbal cues OR nonverbal decoding OR decoding of nonverbal OR decoding affective
25= empathic accuracy
26= CARAT (Communication of Affect Receiving Ability Test)
27= ER-40 or ER40 or Penn Emotion Recognition Task
28= CNB or Computerized Neurocognitive Battery [Penn] [NOTE 27 and 28 are both ER40]
29= Cambridge Neuropsychological Test Automated Battery [CANTAB] + emotion
88 = published or published+ found by serendipity (not via a formal search; could be something we had, ran across, or was sent unrequested by somebody)
89= from a gender-accuracy meta-analysis bibliography
90= from an accuracy meta-analysis that isn't specifically on gender
91= Unpublished, but not if found in one of the numbered searches as a thesis or dissertation—those get the number of the search they were found in

NOTE: We did not search specifically for the MSCEIT (Mayer-Salovey-Caruso Emotional Intelligence Test) because so rarely is the Faces part reported separately. But we included the MSCEIT if it came up in an article from another search, serendipity, or unpublished.

Source_kind
1= results were in published article
2= published+ (this means we asked author for more information and they sent it); this code could be for an article, chapter, or dissertation)
4= dissertation or Master's thesis
6= unpublished (not code 4)
7= chapter or book
8= published+ from a gender-accuracy meta-analysis when we couldn't access the ES ourselves (sometimes ES sent by authors to the meta-analysis author on request)

Document date

First_author_gender
1= male
2= female

N_males

N_females

N_total

N_total_independent
This total is entered only once per independent study, so the total headcount for whole meta-analysis could be calculated.

Sample_age (mean of whole sample)

Sample_age_category
1= 8-12 'children'
2= 13-17 'teens'
3= 18-27 'college/university' (this is age, not being a college/university student per se)
4= adults over college age OR a mix of codes 3&4

Internatl_location
1= USA
2= Europe *not* English speaking (not England, Ireland, Scotland, Wales)
3= Australia, New Zealand
4= 2 or more countries
5= Europe *English* speaking (England, Ireland, Scotland, Wales)
6= East and Southeast Asia (China, Singapore, Korea, Thailand, Japan, Taiwan, Hong Kong)
7= sub-Saharan Africa
8= Canada
9= South or Central America including Mexico
10= Mideast (Iran, Israel, Egypt…)
11= South Asia (India, Pakistan)
12= Russia
13= New Guinea

Sample_type
1= children or teens
2= college/university (includes medical students and medical residents)
3= community (online as in Mturk, or recruited in public places, clinics, etc.)
4= mixture

Sample_health
1= nonclinical

Includes all nondiagnosed samples and those called ‘healthy controls’ in clinical studies. Includes nonclinical samples selected for a specific nonpathological characteristic: screened high on nonclinical personality disorders, includes trans/nonbinary but used birth assignment, nonclinical but with a genetic marker, college students high on alexithymia, recreational cocaine users, obese, social media addicted, siblings of schizophrenics, nonclinical but skewed to high psychopathy, at risk for psychosis due to genetics, gay and lesbian, high risk of child abuse (self-report), children of depressed mothers, imbibed alcohol in lab, born prematurely, relatives of schzophrenia patients.

2= cognitively impaired:

neurodegenerative disorder (dementia, Alzheimers), Alzheimer + frontotemporal dementia, Alzheimers, mild cognitive impairment, mental retardation, frontotemporal dementia, presymptomatic dementia, learning disabled, subjective cognitive decline

3= physical illness/disability/injury:

deaf+hard of hearing, spina bifida, post-surgery for brain tumor, ALS, Huntington, stroke, nonsymptomatic Huntington, epilepsy, MS, acquired brain injury, toxoplasmosis, mix of problems (in rehab), stroke, Parkinsons, myotonic dystrophy, post-stroke, temporal lobe epilepsy

4= psychological disorders:

(4-1) psychosis: schizophrenia spectrum disorder, schizophrenia, psychosis, schizoaffective, hi risk for psychosis, hi risk for schizophrenia

(4-2) affective disorders:

depression, depression+anxiety, suicide attempters, high suicide risk, affective disorder, anxiety disorder, major depression remitted, bipolar, panic disorder, intermittent explosive disorder

(4-3) personality disorders:

borderline, avoidant, personality disorder

(4-4) alcoholism

(4-5) miscellaneous mental illness and behavior problems:

high risk for criminality, had or say they need psychiatric therapy, in detention center for various crimes, history with social services and/or offending, serious mental illness, behavior problems, mental health care, emotionally disturbed, conduct disorder

8= neurodevelopmental disorders:

Autism, Autism spectrum disorder, Autism+ADHD, neurodevelopmental disorder, congenital cerebellar damage

Name_health_problem (typed in words)

Participant_ethnicity (60% or higher)

1= Caucasian

2= Chinese, other East or Southeast Asian (Korea, Japan, Thailand, Taiwan, etc.)

3= sub-Saharan African

4= African American in USA

5= South Asia (India, Pakistan)

6= mix of 2 or several (no group > 60%)

7= New Guinea

8= Black in S or Central America

Name_of_test (typed in words)

Test_code
This refers to the basic stimulus set and it includes all variations on it. For example, Ekman's Pictures of Facial Affect (POFA) are used many ways and under various names (e.g., FEEST)—different exposures, morphed or not, different numbers of faces, etc. If POFA faces were used, it has Test Code 17.

1 and 5= tests with no capitalized name, one-off, likely not used elsewhere; also some named tests that were rarely used
2= Ebner FACES
3= Reading the Mind in the Eyes, original stimuli (RMET)
4= Degraded Facial Affect Test (DFAR)
6= The Awareness of Social Inference Test (TASIT)
7= Diagnostic Analysis of Nonverbal Accuracy (DANVA) adult faces
8= DANVA child faces
10= DANVA adult voices
11= DANVA child voices
13= DANVA postures
14= ER40 (from Penn Computerized Neurocognitive Battery)
15= MSCEIT faces
16= CANTAB faces (Cambridge Neuropsychological Test Automated Battery)
17= Ekman faces (Pictures of Facial Affect, POFA) (includes BART)
18= Geneva Emotion Recognition Test (GERT)
19= Karolinska faces (KDEF)
20= Bell-Lysaker Emotion Recognition Test (BLERT)
21= Amsterdam Directed Facial Expressions Set (ADFES)
22= NimStim faces
24= RMET-style Asian eyes
25= Radboud faces
26= Empathic accuracy Zaki method (method of continuous negative-positive ratings)
27= Empathic accuracy (original method of Ickes—matching thought/feelings in words)
28= Empathic accuracy (ratings on emotion words made after whole interaction)
29= Korean Facial Expressions of Emotion (KOFFEE)
33= Digitally created faces (avatars)
34= Japanese and Caucasian Facial Expressions of Emotion (JACFEE)
38= Montreal Affective Voices
40= METT or SETT
41= VENEC vocal clips
42= UC Davis faces
43= Test of Accurate Perception of Patients' Affect (TAPPA)
45= PONS (or half)
47= PONS child audio
48= PONS male audio
50= Montagne faces

51= Communication of Affect Receiving Accuracy Test (CARAT)
52= Izard faces
54= Vienna Emotion Recognition Test
55= Emotion Recognition Test (Merton)

Test_code_with_10_or_fewer_ES_coded_as_60
60= original codes 1 or 5 and all tests with <10 usages; all tests with 10 or more usages have their original code

Test_code_10_or_more_ES
0= test has <10 usages
1= test has 10 or more usages

NOTE: there are several more of these recoded 'test code' variables, grouping in different ways.

Target_gender
1= M only
2= F only
3= M&F

Target_age
1= children/adolescents
2= adults
3= both

Target_ethnicity (80% or more of targets)
1=Caucasian
2=Black
3= Asian
4= Latinex
5= mixed
6= South Asia (India, Pakistan)
7= middle Eastern countries

Cue_channel
1= face only
2= voice only (unfiltered/unmasked)
3= voice only (filtered or standard content or no content)
4= body or hands only
5= words only (not used unless it is combined in a multichannel score)
6= full (face, body, unfiltered/unmasked voice with uncontrolled content)
7=eyes
8= face, body, and content masked speech (this includes standard content and electronically filtered; also 'semantically neutral' content as said about TASIT)
10= face&body together
11= face and content masked speech

12= multichannel--multiple cue modalities shown separately but combined in total score
13= mouth only

Cues_in_multichannel (listing of the cue channels from previous variable that are combined in the multichannel total; these lists may be incomplete)

Is_test_multichannel

0=no (the test has only one of the cues listed in Cue_channel)
1=yes (different cue modalities are separately shown and then combined—example: PONS Face and Body test does not show the whole person's face and body simultaneously, but rather each is shown separately and combined in total score)

Number_of_items

Cue_content
1= emotion (includes EA continuous rating method, as in the Zaki correlational method)
2= situational affect (this is always PONS)
6= "thought or feeling" (applies to original Ickes method)

Dynamic
1= static (photos, avatars; or static photos that are morphed to show different intensities of expression, sometimes assembled to show apparent motion but it was artificially produced)
2= yes--dynamic. Not coded for voice-only because that's dynamic by definition.

Spontaneous
1= no—posed
2= yes—spontaneous

Male_mean or percentage accuracy, Male_SD

Female_mean or percentage accuracy, Female_SD

ES_r (ES = effect size)
This is the point-biserial (Pearson) correlation between gender and accuracy scores. Mostly, we calculated this from means and SDs. Occasionally the author or the publication offered the d or r; very rarely, we used a standardized regression coefficient. Signage is - if men higher, + if women higher; 999 if the *only* result is 'not significant'; 888 for incomplete report of gender results. The 999 and 888 results are excluded from the main data file.

ES_rz
This is the Fisher-z normalization of the Pearson r. Signage is - if men higher, + if women higher; 999 if the *only* result is 'not significant'; 888 for incomplete report of gender results. The 999 and 888 results are excluded from the main data file.

Adjusted
1= unadjusted (ES based on means and SDs, or t-test)
2= adjusted (ES came out of multi-way ANOVA or another analysis that included covariates)
